# Supplementary material for: A Comprehensive Cheminformatics Analysis of Structural Features Affecting the Binding Activity of Fullerene Derivatives
Source: Nanomaterials (Basel). 2020 Jan 2;10(1):90. doi: 10.3390/nano10010090 (PMC7023229; doi:10.3390/nano10010090)
Supplement: Supplementary file 1 [file nanomaterials-10-00090-s001.zip › Supplementary material final.docx]

The comprehensive chemoinformatics analysis of structural features affected the binding activity of fullerene derivatives

Natalja Fjodorova ^1^*, Marjana Novic ^1^ Katja Venko ^1^ and Bakhtiyor Rasulev ^2,^

# Supplementary material

# Table of content:

**Table S1.** The list of 169 FDs with SMILE codes, molecular formula, indication of saturation (S) and binding activity (Av_Bscores values). S2-S61.

**Tables S2-S8.** The classification of FDs dependent on type of bond of substituent groups attached to the fullerene C60 core. S62-S87.

**Table S2.** **GROUP 1:** FDs with functional groups attached to the C60 core with single bond with indication the level of binding activity. S62-S63.

**Table S3.** **GROUP 2**: FDs with functional groups attached to the C60 core with cyclopropane 3-membered ring with indication the level of binding activity. S64-S69.

**Table S4.** **GROUP 3:** FDs contained the substituent groups attached to the C60 core with pyrrolidine (5-membered ring) or pyridine (6-membered) heterocycles containing nitrogen with indication the level of binding activity. S70-S74.

**Table S5.** **GROUP 4:** FDs contained the substituent groups attached to the C60 core with 6-membered (cyclohexane) ring) with indication the level of binding activity. S75-S77.

**Table S6.** **GROUP 5:** FDs contained the substituent groups attached to the C60 core with benzene (aromatic 6-membered) ring with indication the level of binding activity. S78-S85.

**Table S7.** **GROUP 6**: FDs contained the FDs substituent s attached to the C60 core with fused pair of 6-membered rings with indication the level of binding activity. S86.

**Table S8.** **GROUP 7:** FDs contained the FDs substituent groups attached to the C60 core with bridged bicycle rings with indication the level of binding activity. S87.

**Table S9.** Correlation between 27 descriptors (25 drug like descriptors plus (Qpolrz+TD)) and Bscores with correlation coefficients greater than 0.6. S113.

**Table S10.** The differences between saturated and non-saturated fullerene derivatives. S114-S116.

**Figure 1S.** The differences in binding activity (∆Bscores) between the most active FDs and pristine fullerene C_60_. S88.

**Figure 2S.** The architecture of CP ANN. S90.

# The list of considered proteins. S89.

# The detailed description of neural networks used in the study. S90-S91.

# Selection of the most significant proteins. S92.

**Table S1.** The list of 169 FDs with SMILE codes, molecular formula, indication of saturation (S) and binding activity (Av_Bscores values).

| **FD_ID** | **Molecule** | **SMILE notation** | **Molecular Formula** | **Saturated (S)** | **Av_Bscores** |
| --- | --- | --- | --- | --- | --- |
| 1 |  | c1cccc(c1)[C@@]1([C@]23c4c5c6c7c8c9[C@@H]%10[C@@H]%11c%12c%13c%14[C@H]%15[C@H]%16[C@H]%17[C@@H]%13[C@@H]%11[C@H]%11[C@H]%13[C@@H]%10[C@@H]8[C@H]8[C@H]%10[C@@H]7c5c5c7c4c4c3c(c%14c3c%12c9c6[C@]123)c%15c1c4[C@@H]2[C@H]7[C@H]3[C@@H]4[C@@H]6[C@H]([C@@H]%10[C@@H]53)[C@H]8[C@H]%13[C@H]3[C@@H]6[C@@H]5[C@H]([C@@H]%17[C@@H]%113)[C@H]%16[C@H]1[C@H]2[C@H]45)[C@H](c1ccccc1)O | C74H12O |  | 5598,96 |
| 2 |  | c12c3c4[C@H]5[C@H]6[C@H]7[C@@H]3[C@H]3[C@@H]2[C@H]2c8c9c1c1[C@]%10%11c9c9c%12c8[C@@H]8[C@H]2[C@@H]2[C@H]3[C@H]3[C@H]%13[C@@H]2[C@H]2[C@@H]8[C@H]8[C@@H]%12c%12c9c9[C@@]%10(c%10c(c41)c5c1c4c%10c9c5c%12[C@H]9[C@H]%10[C@@H]5[C@@H]4[C@H]4[C@@H]1[C@@H]6[C@@H]([C@@H]73)[C@H]1[C@@H]%13[C@H]([C@@H]%10[C@@H]41)[C@@H]2[C@@H]89)C%11(c1ccc(cc1)CCN)c1ccc(cc1)CCN | C77H20N2 |  | 6163,475 |
| 3 |  | c12[C@@H]3[C@H]4[C@H]5[C@H]6[C@H]7[C@@H]3c3c2c2c8c9c1[C@@H]1[C@@H]%10c9c9c%11c8[C@]8%12c2c2c3[C@H]3c%13c2c2[C@@]8(c8c%11c%11[C@@H]9[C@H]9[C@@H]%10[C@H]%10[C@@H]([C@H]41)[C@@H]5[C@@H]1[C@@H]4[C@H]%10[C@H]9[C@@H]5c%11c9c%10[C@@H]5[C@@H]4[C@H]4[C@@H]1[C@@H]6[C@@H]([C@@H]73)[C@H]1c%13c(c%10[C@@H]41)c2c89)[C@H](C[C@@H]([C@@H]%12C(C)C)O)C(C)C | C70H20O |  | 5258,403 |
| 4 |  | c12c3c4[C@H]5[C@H]6[C@H]7[C@@H]3[C@H]3[C@@H]2[C@H]2c8c9c1c1[C@]%10%11c9c9c%12c8[C@@H]8[C@H]2[C@@H]2[C@H]3[C@H]3[C@H]%13[C@@H]2[C@H]2[C@@H]8[C@H]8[C@@H]%12c%12c9c9[C@@]%10(c%10c(c41)c5c1c4c%10c9c5c%12[C@H]9[C@H]%10[C@@H]5[C@@H]4[C@H]4[C@@H]1[C@@H]6[C@@H]([C@@H]73)[C@H]1[C@@H]%13[C@H]([C@@H]%10[C@@H]41)[C@@H]2[C@@H]89)C%11(c1ccc(cc1)CCNC(=O)CCC(=O)O)c1ccc(cc1)CCNC(=O)CCC(=O)O | C85H28N2O6 |  | 7257,42 |
| 5 |  | [C@H]12[C@@H]3[C@H]4c5c6[C@H]7[C@@H]3[C@H]3[C@@H]2[C@H]2[C@H]8[C@H]9[C@@H]1c1c%10[C@H]9[C@@H]9[C@@H]%11[C@H]8[C@@H]8[C@H]2[C@@H]2[C@H]3[C@H]3[C@H]%12[C@@H]2[C@H]2[C@@H]8[C@H]8[C@@H]%11c%11c9c9c%10c%10c(c41)c5c1[C@@]4([C@H]%10c9c5c%11[C@H]9c%10c5c4c4c1c6c([C@@H]73)c1[C@@H]%12[C@H](c%10c41)[C@@H]2[C@@H]89)NCCCCCC(=O)O | C66H71NO2 | S | 5421,724 |
| 6 |  | [C@H]12[C@@H]3[C@H]4c5c6[C@H]7[C@@H]3[C@H]3[C@@H]2[C@H]2[C@H]8[C@H]9[C@@H]1c1c%10[C@H]9[C@@H]9[C@@H]%11[C@H]8[C@@H]8[C@H]2[C@@H]2[C@H]3[C@H]3[C@H]%12[C@@H]2[C@H]2[C@@H]8[C@H]8[C@@H]%11c%11c9c9c%10c%10c(c41)c5c1[C@@]4([C@H]%10c9c5c%11[C@H]9c%10c5c4c4c1c6[C@@H]([C@@H]73)[C@H]1[C@@H]%12[C@H](c%10c41)[C@@H]2[C@@H]89)N[C@@H](CCCC/C=C/CCCCCCCC)CCC(=O)O | C78H35NO2 |  | 6922,521 |
| 7 |  | [C@H]12[C@@H]3[C@H]4c5c6[C@H]7[C@@H]3[C@H]3[C@@H]2[C@H]2[C@H]8[C@H]9[C@@H]1c1c%10[C@H]9[C@@H]9[C@@H]%11[C@H]8[C@@H]8[C@H]2[C@@H]2[C@H]3[C@H]3[C@H]%12[C@@H]2[C@H]2[C@@H]8[C@H]8[C@@H]%11c%11c9c9c%10c%10c(c41)c5c1[C@@]4([C@H]%10c9c5c%11[C@H]9c%10c5c4c4c1c6c([C@@H]73)c1[C@@H]%12[C@H](c%10c41)[C@@H]2[C@@H]89)NCCCC[C@@H](C(=O)O)N | C66H14N2O2 |  | 5585,332 |
| 8 |  | C12=C3[C@@H]4[C@@H]5C6=C2[C@@H]2[C@@H]7[C@H]1[C@H]1c8c9[C@@H]7[C@@H]7c%10c%11[C@@H]%12[C@@H]%13c%14c%15c%16c%17c%18c%19c%20c(c4c4[C@@H]%21[C@H]5[C@@H]5[C@H]6[C@@H]6[C@H]2[C@H]7[C@H]%12[C@@H]6[C@H]2[C@@H]5[C@H]5[C@@H]%21c(c%194)c%17[C@@H]%15[C@H]5[C@@H]%132)c3c1c1c%20[C@H]%18[C@]2(c(c81)c(c(c%162)c%11%14)c9%10)NC(=N)NCCC[C@@H](C(=O)O)N | C66H14N4O2 |  | 5710,1 |
| 9 |  | OC(=O)CC(NC(=O)N[C@]12[C@H]3c4c5c6c7c8[C@@H]9[C@H]%10[C@@H]%11[C@@H]%12[C@H]%13c%14c%15[C@H]%16[C@@H]%12[C@H]%12[C@@H]%11[C@H]%11[C@@H]9[C@H]9[C@H]%17c8c5c5[C@H]%17[C@@H]8[C@@H]%17[C@H]9[C@H]%11[C@@H]9[C@H]%12[C@H]%11[C@H]%12[C@@H]9[C@@H]%17[C@H]9[C@@H]8[C@H]8c5c4c4c8c5[C@H]9[C@H]%12c8c([C@@H]%16%11)c%15c(c1c%14c(c%13c7%10)c36)c(c24)c58)CC(=O)O | C66H10N2O5 |  | 5591,372 |
| 10 |  | c12c3c4[C@H]5[C@H]6[C@H]7[C@@H]3[C@H]3[C@@H]2[C@H]2c8c9c1c1[C@]%10%11c9c9c%12c8[C@@H]8[C@H]2[C@@H]2[C@H]3[C@H]3[C@H]%13[C@@H]2[C@H]2[C@@H]8[C@H]8[C@@H]%12c%12c9c9[C@@]%10(c%10c(c41)c5c1c4c%10c9c5c%12[C@H]9[C@H]%10c5c4[C@H]4[C@@H]1[C@@H]6[C@@H]([C@@H]73)[C@H]1[C@@H]%13[C@H]([C@@H]%10[C@@H]41)[C@@H]2[C@@H]89)[C@]%11(c1ccccc1)c1ccc(cc1)CCCC(=O)NC(CO)(CO)CO | C81H25NO4 |  | 6779,54 |
| 11 |  | c12[C@@H]3[C@H]4[C@H]5[C@H]6[C@H]7[C@@H]3c3c2c2c8c9c1[C@@H]1[C@@H]%10c9c9c%11c8[C@]8%12c2c2c3[C@H]3c%13c2c2[C@@]8(c8c%11c%11[C@@H]9[C@H]9[C@@H]%10[C@H]%10[C@@H]([C@H]41)[C@@H]5[C@@H]1[C@@H]4[C@H]%10[C@H]9[C@@H]5c%11c9c%10[C@@H]5[C@@H]4[C@H]4[C@@H]1[C@@H]6[C@@H]([C@@H]73)[C@H]1c%13c(c%10[C@@H]41)c2c89)[C@@H](C/C(=N\OCC(=O)O)/C%12)OC | C67H13NO4 |  | 5487,927 |
| 12 |  | c12[C@@H]3[C@H]4[C@H]5[C@H]6[C@H]7[C@@H]3c3c2c2c8c9c1[C@@H]1[C@@H]%10c9c9c%11c8[C@]8%12c2c2c3[C@H]3c%13c2c2[C@@]8(c8c%11c%11[C@@H]9[C@H]9[C@@H]%10[C@H]%10[C@@H]([C@H]41)[C@@H]5[C@@H]1[C@@H]4[C@H]%10[C@H]9[C@@H]5c%11c9c%10[C@@H]5[C@@H]4[C@H]4[C@@H]1[C@@H]6[C@@H]([C@@H]73)[C@H]1c%13c(c%10[C@@H]41)c2c89)CC/C(=N\OCC(=O)O)/C%12 | C66H9NO3 |  | 5304,322 |
| 13 |  | c12[C@@H]3[C@H]4[C@H]5[C@H]6[C@H]7[C@@H]3c3c2c2c8c9c1[C@@H]1[C@@H]%10c9c9c%11c8[C@]8%12c2c2c3[C@H]3c%13c2c2[C@@]8(c8c%11c%11[C@@H]9[C@H]9[C@@H]%10[C@H]%10[C@@H]([C@H]41)[C@@H]5[C@@H]1[C@@H]4[C@H]%10[C@H]9[C@@H]5c%11c9c%10[C@@H]5[C@@H]4[C@H]4[C@@H]1[C@@H]6[C@@H]([C@@H]73)[C@H]1c%13c(c%10[C@@H]41)c2c89)[C@H](C=C[C@H]%12C)COC(=O)CCC(=O)O | C70H72O4 | S | 5704,231 |
| 14 |  | c12[C@@H]3[C@H]4[C@H]5[C@H]6[C@H]7[C@@H]3c3c2c2c8c9c1[C@@H]1[C@@H]%10c9c9c%11c8[C@]8%12c2c2c3[C@H]3c%13c2c2[C@@]8(c8c%11c%11[C@@H]9[C@H]9[C@@H]%10[C@H]%10[C@@H]([C@H]41)[C@@H]5[C@@H]1[C@@H]4[C@H]%10[C@H]9[C@@H]5c%11c9c%10[C@@H]5[C@@H]4[C@H]4[C@@H]1[C@@H]6[C@@H]([C@@H]73)[C@H]1c%13c(c%10[C@@H]41)c2c89)C[C@H](CC%12)OC(=O)CCC(=O)O | C68H12O4 |  | 5566,893 |
| 15 |  | c12[C@@H]3[C@H]4[C@H]5[C@H]6[C@H]7[C@@H]3c3c2c2c8c9c1[C@@H]1[C@@H]%10c9c9c%11c8[C@]8%12c2c2c3[C@H]3c%13c2c2[C@@]8(c8c%11c%11[C@@H]9[C@H]9[C@@H]%10[C@H]%10[C@@H]([C@H]41)[C@@H]5[C@@H]1[C@@H]4[C@H]%10[C@H]9[C@@H]5c%11c9c%10[C@@H]5[C@@H]4[C@H]4[C@@H]1[C@@H]6[C@@H]([C@@H]73)[C@H]1c%13c(c%10[C@@H]41)c2c89)C=C/C(=N\OCC(=O)O)/C%12 | C66H7NO3 |  | 5292,03 |
| 16 |  | N(=C\1/[C@H]2[C@]34[C@]5(c6c7c8c9c%10[C@@H]%11[C@H]%12[C@@H]%13[C@@H]%14[C@H]%15c%16c%17[C@H]%18[C@@H]%14[C@H]%14[C@@H]%13[C@H]%13[C@@H]%11[C@H]%11[C@H]%19c%10c7c7[C@H]%19[C@@H]%10[C@@H]%19[C@H]%11[C@H]%13[C@@H]%11[C@H]%14[C@H]%13[C@H]%14[C@@H]%11[C@@H]%19[C@H]%11[C@@H]%10[C@H]%10c7c6c6c%10c7[C@H]%11[C@H]%14[C@@H]%10[C@H]([C@@H]%18%13)c%17c(c3c%16c(c%15c9%12)c58)c(c46)c7%10)[C@]2(C)CCC1)\OCC(=O)O | C69H13NO3 |  | 5465,162 |
| 17 |  | c12[C@@H]3[C@H]4[C@H]5[C@H]6[C@H]7[C@@H]3c3c2c2c8c9c1[C@@H]1[C@@H]%10c9c9c%11c8[C@]8%12c2c2c3[C@H]3c%13c2c2[C@@]8(c8c%11c%11[C@@H]9[C@H]9[C@@H]%10[C@H]%10[C@@H]([C@H]41)[C@@H]5[C@@H]1[C@@H]4[C@H]%10[C@H]9[C@@H]5c%11c9c%10[C@@H]5[C@@H]4[C@H]4[C@@H]1[C@@H]6[C@@H]([C@@H]73)[C@H]1c%13c(c%10[C@@H]41)c2c89)[C@@]%12(c1ccccc1)c1ccc2c(c1)OCCOCCOCCOCCOCCO2 | C83H28O6 |  | 6766,045 |
| 18 |  | c12c3c4c5c6c7c3c3c2c2c8c9c1[C@]1%10c%11c9c9[C@@H]%12[C@H]8C8=C%13c%14c%12c%12c9c9c%11c%11c(c5c5[C@]%15%16[C@]%11(c9c9c%12c%11c%12c9c%15c9c5c6[C@H]5c7c6c3[C@@H]([C@@H]28)[C@@H]2C6=C3[C@H](c%12c9[C@@H]53)[C@H](c%14%11)[C@H]%132)C[C@@H](CC%16)O)[C@]41CC[C@@H](C%10)O | C68H**72**O2 | S | 4953,817 |
| 19 |  | c12c3c4c5c6c7c3[C@H]3c2c2c8c9c1[C@]1%10c%11c9c9[C@@H]%12[C@H]8[C@@H]8[C@H]2[C@@H]2[C@H]3[C@H]3[C@H]%13[C@@H]2[C@H]2[C@@H]8[C@H]8[C@@H]%12c%12c9c9c%11c%11c(c5c5[C@]%14%15[C@]%11(c9c9c%12[C@H]%11c%12c9c%14c9c5c6[C@@H]([C@@H]73)[C@H]3[C@@H]%13[C@H](c%12c93)[C@@H]2[C@@H]8%11)CN(C%15)C)[C@]41CN(C%10)C | C66H16N2 |  | 4934,548 |
| 20 |  | c12c3c4c5c6c7c3[C@H]3c2c2c8c9c1[C@]1%10c%11c9c9[C@@H]%12[C@H]8[C@@H]8[C@H]2[C@@H]2[C@H]3[C@H]3[C@H]%13[C@@H]2[C@H]2[C@@H]8[C@H]8[C@@H]%12c%12c9c9c%11c%11c(c5c5[C@]%14%15[C@]%11(c9c9c%12C%11=C8[C@H]2[C@H]2c8c%11c9c%14c9c5c6[C@@H]([C@@H]73)[C@@H]([C@@H]%132)c89)CC(=O)C[C@H]%15OC)[C@]41[C@@H](CC(=O)C%10)OC | C70H20O4 |  | 5179,604 |
| 21 |  | c12[C@@H]3[C@H]4[C@H]5[C@H]6[C@H]7[C@@H]3c3c2c2c8c9c1[C@@H]1[C@@H]%10c9c9c%11c8[C@]8%12c2c2c3[C@H]3c%13c2c2[C@@]8(c8c%11c%11[C@@H]9[C@H]9[C@@H]%10[C@H]%10[C@@H]([C@H]41)[C@@H]5[C@@H]1[C@@H]4[C@H]%10[C@H]9[C@@H]5c%11c9[C@H]%10[C@@H]5[C@@H]4[C@H]4[C@@H]1[C@@H]6[C@@H]([C@@H]73)[C@H]1c%13c([C@@H]%10[C@@H]41)c2c89)C(=C(C(=C%12C(=O)O)O)O)C(=O)O | C66H62O6 | S | 4962,804 |
| 22 |  | c12[C@@H]3[C@H]4C5=C6C7=C3c3c2c2c8c9c1[C@@H]1[C@@H]%10c9c9c%11c8[C@]8%12c2c2c3[C@H]3c%13c2c2[C@@]8(c8c%11c%11[C@@H]9[C@H]9[C@@H]%10[C@H]%10[C@@H]([C@H]41)[C@@H]5[C@@H]1[C@@H]4[C@H]%10[C@H]9[C@@H]5c%11c9c%10[C@@H]5[C@@H]4[C@H]4[C@@H]1[C@@H]6[C@@H]([C@@H]73)[C@H]1c%13c(c%10[C@@H]41)c2c89)C(=C(C(=C%12O)C(=C(O)O)O)O)O | C66H64O6 | S | 5149,697 |
| 23 |  | c12[C@@H]3[C@H]4[C@H]5[C@H]6[C@H]7[C@@H]3c3c2c2c8c9c1[C@@H]1[C@@H]%10c9c9c%11c8[C@]8%12c2c2c3[C@H]3c%13c2c2[C@@]8(c8c%11c%11[C@@H]9[C@H]9[C@@H]%10[C@H]%10[C@@H]([C@H]41)[C@@H]5[C@@H]1[C@@H]4[C@H]%10[C@H]9[C@@H]5c%11c9c%10[C@@H]5[C@@H]4[C@H]4[C@@H]1[C@@H]6[C@@H]([C@@H]73)[C@H]1c%13c(c%10[C@@H]41)c2c89)C(=C(C(=C%12C(=O)O)C(=O)O)C(=O)O)C(=O)O | C68H62O8 | S | 5274,947 |
| 24 |  | [C@H]12[C@@H]3[C@H]4[C@H]5[C@H]6[C@H]7[C@@H]3c3c2c2c8c9[C@@H]1[C@@H]1[C@@H]%10c9c9c%11c8[C@]8%12c2c2c3[C@H]3c%13c2c2[C@@]8(c8c%11c%11[C@@H]9[C@H]9[C@@H]%10[C@H]%10[C@@H]([C@H]41)[C@@H]5[C@@H]1[C@@H]4[C@H]%10[C@H]9[C@@H]5c%11c9[C@H]%10[C@@H]5[C@@H]4[C@H]4[C@@H]1[C@@H]6[C@@H]([C@@H]73)[C@H]1c%13c([C@@H]%10[C@@H]41)c2c89)C(=C(C(=C%12O)O)O)O | C64H62O4 | S | 4779,087 |
| 25 |  | c12c3c4c5c6c7c3c3c2c2c8c9c1c1c%10c9c9c%11c8[C@]8%12c2c2c3[C@H]3c%13c2c2[C@@]8(c8c%11c%11[C@@H]9[C@H]9c%10c%10c(c41)[C@@]15[C@@]4(c5c%10c9c9c%11c%10c%11c9c5c5c4c6c([C@@H]73)c3c%13c(c%11c53)c2c8%10)C=CC=C1)C=CC=C%12 | C68H64 | S | 4999,765 |
| 26 | 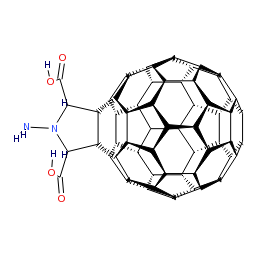 | c12[C@@H]3[C@H]4[C@H]5[C@H]6[C@H]7[C@@H]3c3c2c2c8c9c1[C@@H]1c%10c9c9[C@]%11%12[C@]8(c8c2c2c3[C@H]3[C@H]%13c2c2c8c8c%11c%11c9c9c%10[C@H]%10[C@@H]([C@H]41)[C@@H]5[C@@H]1[C@@H]4[C@H]%10c9c5c%11c9[C@H]%10[C@@H]5[C@@H]4[C@H]4[C@@H]1[C@@H]6[C@@H]([C@@H]73)[C@H]1[C@@H]%13[C@H]([C@@H]%10[C@@H]41)[C@@H]2c89)[C@@H](N([C@@H]%12C(=O)O)N)C(=O)O | C64H64N2O4 | S | 4880,39 |
| 27 | 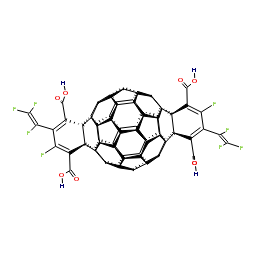 | c12c3c4c5c6c7c3c3c2c2c8c9c1c1c%10c9c9c%11c8[C@]8%12c2c2c3[C@H]3c%13c2c2[C@@]8(c8c%11c%11[C@@H]9[C@H]9c%10c%10c(c41)[C@@]15[C@@]4(c5c%10c9c9c%11c%10c%11c9c5c5c4c6c([C@@H]73)c3c%13c(c%11c53)c2c8%10)C(=C(C(=C1C(=O)O)F)C(=C(F)F)F)C(=O)O)C(=C(C(=C%12C(=O)O)C(=C(F)F)F)F)C(=O)O | C76H60O8F8 | S | 6089,026 |
| 28 | 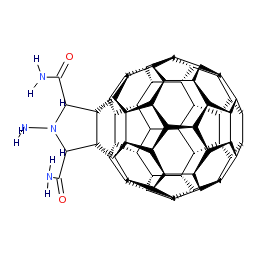 | c12[C@@H]3[C@H]4[C@H]5[C@H]6[C@H]7[C@@H]3c3c2c2c8c9c1[C@@H]1c%10c9c9[C@]%11%12[C@]8(c8c2c2c3[C@H]3[C@H]%13c2c2c8c8c%11c%11c9c9c%10[C@H]%10[C@@H]([C@H]41)[C@@H]5[C@@H]1[C@@H]4[C@H]%10c9c5c%11c9[C@H]%10[C@@H]5[C@@H]4[C@H]4[C@@H]1[C@@H]6[C@@H]([C@@H]73)[C@H]1[C@@H]%13[C@H]([C@@H]%10[C@@H]41)[C@@H]2c89)[C@@H](N([C@@H]%12C(=O)N)N)C(=O)N | C64H66N4O2 | S | 4936,039 |
| 29 | 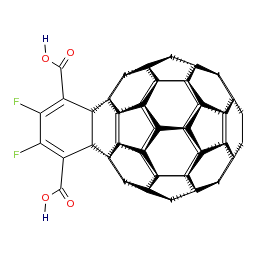 | c12[C@@H]3[C@H]4[C@H]5[C@H]6[C@H]7[C@@H]3c3c2c2c8c9c1[C@@H]1[C@@H]%10c9c9c%11c8[C@]8%12c2c2c3[C@H]3c%13c2c2[C@@]8(c8c%11c%11[C@@H]9[C@H]9[C@@H]%10[C@H]%10[C@@H]([C@H]41)[C@@H]5[C@@H]1[C@@H]4[C@H]%10[C@H]9[C@@H]5c%11c9c%10[C@@H]5[C@@H]4[C@H]4[C@@H]1[C@@H]6[C@@H]([C@@H]73)[C@H]1c%13c(c%10[C@@H]41)c2c89)C(=C(C(=C%12C(=O)O)F)F)C(=O)O | C66H60O4F2 | S | 5022,927 |
| 30 | 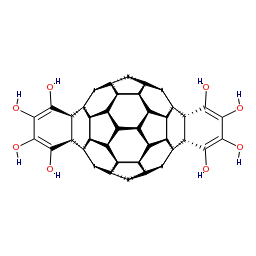 | c12c3c4c5c6c7c3c3c2c2c8c9c1c1c%10c9c9c%11c8[C@]8%12c2c2c3[C@H]3c%13c2c2[C@@]8(c8c%11c%11[C@@H]9[C@H]9c%10c%10c(c41)[C@@]15[C@@]4(c5c%10c9c9c%11c%10c%11c9c5c5c4c6c([C@@H]73)c3c%13c(c%11c53)c2c8%10)C(=C(C(=C1O)O)O)O)C(=C(C(=C%12O)O)O)O | C68H64O8 | S | 5188,208 |
| 31 | 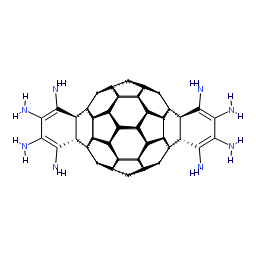 | c12c3c4c5c6c7c3c3c2c2c8c9c1c1c%10c9c9c%11c8[C@]8%12c2c2c3[C@H]3c%13c2c2[C@@]8(c8c%11c%11[C@@H]9[C@H]9c%10c%10c(c41)[C@@]15[C@@]4(c5c%10c9c9c%11c%10c%11c9c5c5c4c6c([C@@H]73)c3c%13c(c%11c53)c2c8%10)C(=C(C(=C1N)N)N)N)C(=C(C(=C%12N)N)N)N | C68H72N8 | S | 5324,978 |
| 32 | 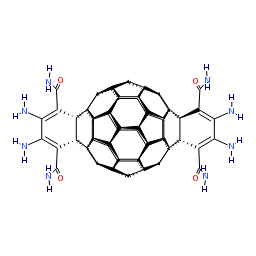 | c12c3c4c5c6c7c3c3c2c2c8c9c1c1c%10c9c9c%11c8[C@]8%12c2c2c3[C@H]3c%13c2c2[C@@]8(c8c%11c%11[C@@H]9[C@H]9c%10c%10c(c41)[C@@]15[C@@]4(c5c%10c9c9c%11c%10c%11c9c5c5c4c6c([C@@H]73)c3c%13c(c%11c53)c2c8%10)C(=C(C(=C1C(=O)N)N)N)C(=O)N)C(=C(C(=C%12C(=O)N)N)N)C(=O)N | C72H72N8O4 | S | 5676,525 |
| 33 | 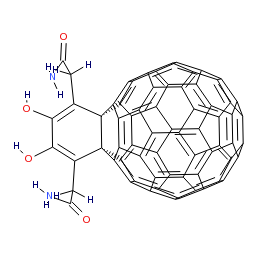 | c12[C@@H]3[C@H]4[C@H]5[C@H]6[C@H]7[C@@H]3c3c2c2c8c9c1[C@@H]1[C@@H]%10c9c9c%11c8[C@]8%12c2c2c3[C@H]3c%13c2c2[C@@]8(c8c%11c%11[C@@H]9[C@H]9[C@@H]%10[C@H]%10[C@@H]([C@H]41)[C@@H]5[C@@H]1[C@@H]4[C@H]%10[C@H]9[C@@H]5c%11c9c%10[C@@H]5[C@@H]4[C@H]4[C@@H]1[C@@H]6[C@@H]([C@@H]73)[C@H]1c%13c(c%10[C@@H]41)c2c89)C(=C(C(=C%12CC(=O)N)O)O)CC(=O)N | C68H10N2O4 | S | 5133,148 |
| 34 | 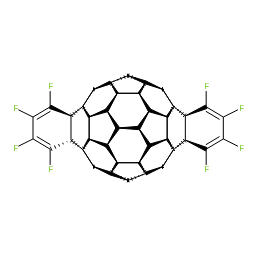 | c12c3c4c5c6c7c3c3c2c2c8c9c1c1c%10c9c9c%11c8[C@]8%12c2c2c3[C@H]3c%13c2c2[C@@]8(c8c%11c%11[C@@H]9[C@H]9c%10c%10c(c41)[C@@]15[C@@]4(c5c%10c9c9c%11c%10c%11c9c5c5c4c6c([C@@H]73)c3c%13c(c%11c53)c2c8%10)C(=C(C(=C1F)F)F)F)C(=C(C(=C%12F)F)F)F | C68H56F8 | S | 5334,435 |
| 35 | 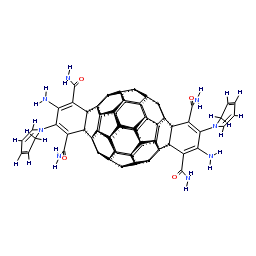 | c12c3c4c5c6c7c3c3c2c2c8c9c1c1c%10c9c9c%11c8[C@]8%12c2c2c3[C@H]3c%13c2c2[C@@]8(c8c%11c%11[C@@H]9[C@H]9c%10c%10c(c41)[C@@]15[C@@]4(c5c%10c9c9c%11c%10c%11c9c5c5c4c6c([C@@H]73)c3c%13c(c%11c53)c2c8%10)C(=C(C(=C1C(=O)N)N)N1CC=CC=C1)C(=O)N)C(=C(C(=C%12C(=O)N)N1C=CC=CC1)N)C(=O)N | C82H80N8O4 | S | 6356,594 |
| 36 | 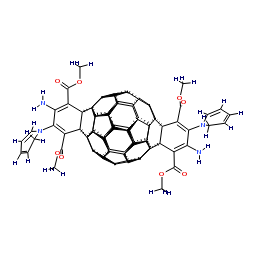 | c12c3c4c5c6c7c3c3c2c2c8c9c1c1c%10c9c9c%11c8[C@]8%12c2c2c3[C@H]3c%13c2c2[C@@]8(c8c%11c%11[C@@H]9[C@H]9c%10c%10c(c41)[C@@]15[C@@]4(c5c%10c9c9c%11c%10c%11c9c5c5c4c6c([C@@H]73)c3c%13c(c%11c53)c2c8%10)C(=C(C(=C1C(=O)OC)N)N1CC=CC=C1)C(=O)OC)C(=C(C(=C%12C(=O)OC)N1C=CC=CC1)N)C(=O)OC | C86H84N4O8 | S | 6709,443 |
| 37 | 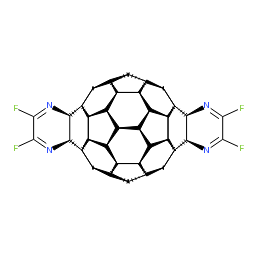 | c12c3c4c5c6c7c3c3c2c2c8c9c1c1c%10c9c9c%11c8[C@]8%12c2c2c3[C@H]3c%13c2c2[C@@]8(c8c%11c%11[C@@H]9[C@H]9c%10c%10c(c41)[C@@]15[C@@]4(c5c%10c9c9c%11c%10c%11c9c5c5c4c6c([C@@H]73)c3c%13c(c%11c53)c2c8%10)N=C(C(=N1)F)F)N=C(C(=N%12)F)F | C64H56N4F4 | S | 5079,321 |
| 38 | 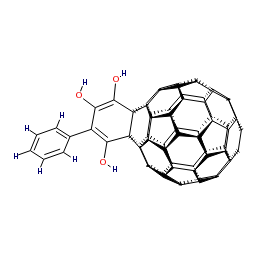 | c12[C@@H]3[C@H]4[C@H]5[C@H]6[C@H]7[C@@H]3c3c2c2c8c9c1[C@@H]1[C@@H]%10c9c9c%11c8[C@]8%12c2c2c3[C@H]3c%13c2c2[C@@]8(c8c%11c%11[C@@H]9[C@H]9[C@@H]%10[C@H]%10[C@@H]([C@H]41)[C@@H]5[C@@H]1C4=C%10C9=C5c%11c9c%10[C@@H]5[C@@H]4[C@H]4[C@@H]1[C@@H]6[C@@H]([C@@H]73)[C@H]1c%13c(c%10[C@@H]41)c2c89)C(=C(C(=C%12O)c1ccccc1)O)O | C70H66O3 | S | 5413,115 |
| 39 | 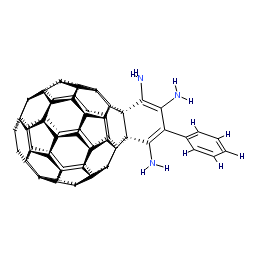 | c12[C@@H]3[C@H]4[C@H]5[C@H]6[C@H]7[C@@H]3c3c2c2c8c9c1[C@@H]1[C@@H]%10c9c9c%11c8[C@]8%12c2c2c3[C@H]3c%13c2c2[C@@]8(c8c%11c%11[C@@H]9[C@H]9[C@@H]%10[C@H]%10[C@@H]([C@H]41)[C@@H]5[C@@H]1[C@@H]4[C@H]%10[C@H]9[C@@H]5c%11c9c%10[C@@H]5[C@@H]4[C@H]4[C@@H]1[C@@H]6[C@@H]([C@@H]73)[C@H]1c%13c(c%10[C@@H]41)c2c89)C(=C(C(=C%12N)c1ccccc1)N)N | C70H69N3 | S | 5512,269 |
| 40 | 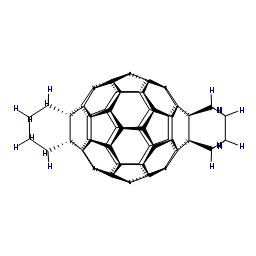 | c12c3c4c5c6c7c3c3c2c2c8c9c1c1c%10c9c9c%11c8[C@]8%12c2c2c3[C@H]3c%13c2c2[C@@]8(c8c%11c%11[C@@H]9[C@H]9c%10c%10c(c41)[C@@]15[C@@]4(c5c%10c9c9c%11c%10c%11c9c5c5c4c6c([C@@H]73)c3c%13c(c%11c53)c2c8%10)CCCC1)CCCC%12 | C68H72 | S | 4941,574 |
| 41 | 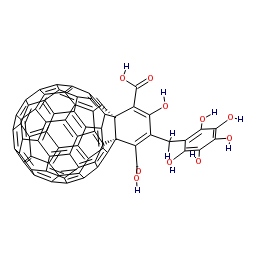 | [C@H]12[C@@H]3[C@H]4[C@H]5[C@H]6[C@H]7[C@@H]3c3c2c2c8c9[C@@H]1[C@@H]1[C@@H]%10c9c9c%11c8[C@]8%12c2c2c3[C@H]3c%13c2c2[C@@]8(c8c%11c%11[C@@H]9[C@H]9[C@@H]%10[C@H]%10[C@@H]([C@H]41)[C@@H]5[C@@H]1[C@@H]4[C@H]%10[C@H]9[C@@H]5c%11c9c%10[C@@H]5[C@@H]4[C@H]4[C@@H]1[C@@H]6[C@@H]([C@@H]73)[C@H]1c%13c(c%10[C@@H]41)c2c89)C(=C(C(=C%12C(=O)O)Cc1c(c(c(c(c1O)O)O)O)O)O)C(=O)O | C73H10O10 |  | 5887,495 |
| 42 | 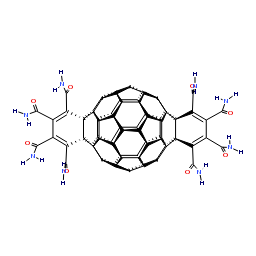 | C(=O)(C1=C(C(=C([C@]23[C@@]41c1c5c6c7c8c9c%10c%11c%12c%13c%14c%10c(c59)c5c1c1c9c%10c%15c%16c%17c%18c%19c%20[C@@H]%21c%22c(c7c7c(c46)c4c2c(c%15c31)c%17c%19c4[C@H]%217)c8c%11c1c%22c%20c2[C@@]3([C@]%121C(=C(C(=C3C(=O)N)C(=O)N)C(=O)N)C(=O)N)c1c(c%182)c%16c%10c([C@@H]%14[C@@H]59)c%131)C(=O)N)C(=O)N)C(=O)N)N | C76H72N8O8 | S | 6100,034 |
| 43 | 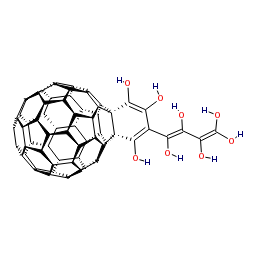 | c12[C@@H]3[C@H]4[C@H]5[C@H]6[C@H]7[C@@H]3c3c2c2c8c9c1[C@@H]1[C@@H]%10c9c9c%11c8[C@]8%12c2c2c3[C@H]3c%13c2c2[C@@]8(c8c%11c%11[C@@H]9[C@H]9[C@@H]%10[C@H]%10[C@@H]([C@H]41)[C@@H]5[C@@H]1[C@@H]4[C@H]%10[C@H]9[C@@H]5c%11c9c%10[C@@H]5[C@@H]4[C@H]4[C@@H]1[C@@H]6[C@@H]([C@@H]73)[C@H]1c%13c(c%10[C@@H]41)c2c89)C(=C(C(=C%12O)/C(=C(/C(=C(O)O)O)\O)/O)O)O | C68H66O8 | S | 5524,722 |
| 44 | 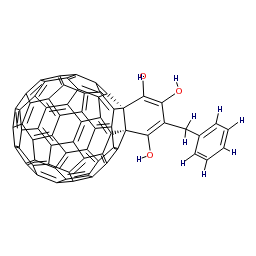 | c12[C@@H]3[C@H]4[C@H]5[C@H]6[C@H]7[C@@H]3c3c2c2c8c9c1[C@@H]1C%10=C%11[C@@H]%12[C@@H]%13c(c%14c8[C@]8%15c2c2c3[C@H]3c%16c2c2[C@@]8(c8c%14c%13c%13[C@H]%14[C@@H]%12[C@H]%12C%11=C([C@H]41)[C@@H]5[C@@H]1[C@H]%12[C@@H]4[C@H]%14c5c%11c(c2c8c%135)c%16[C@@H]2[C@H]([C@@H]73)[C@H]6[C@H]1[C@H]4[C@H]%112)C(=C(C(=C%15O)Cc1ccccc1)O)O)c9%10 | C71H10O3 |  | 5673,377 |
| 45 | 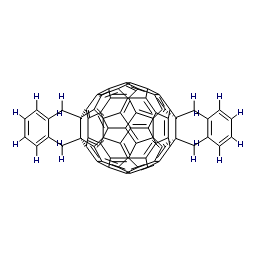 | c12c3c4c5c6c7c3c3c2c2c8c9c1c1c%10c9c9c%11c8[C@]8%12c2c2c3[C@H]3c%13c2c2[C@@]8(c8c%11c%11[C@@H]9[C@H]9c%10c%10c(c41)[C@@]15[C@@]4(c5c%10c9c9c%11c%10c%11c9c5c5c4c6c([C@@H]73)c3c%13c(c%11c53)c2c8%10)Cc2c(cccc2)C1)Cc1c(C%12)cccc1 | C76H16 |  | 5676,836 |
| 46 | 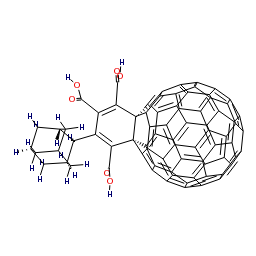 | [C@H]12[C@@H]3[C@H]4[C@H]5[C@H]6[C@H]7[C@@H]3c3c2c2c8c9[C@@H]1[C@@H]1[C@@H]%10c9c9c%11c8[C@]8%12c2c2c3[C@H]3c%13c2c2[C@@]8(c8c%11c%11[C@@H]9[C@H]9[C@@H]%10[C@H]%10[C@@H]([C@H]41)[C@@H]5[C@@H]1[C@@H]4[C@H]%10[C@H]9[C@@H]5c%11c9[C@H]%10[C@@H]5[C@@H]4[C@H]4[C@@H]1[C@@H]6[C@@H]([C@@H]73)[C@H]1c%13c([C@@H]%10[C@@H]41)c2c89)C(=C(C(=C%12C(=O)O)[C@H]1[C@H]2C[C@H]3C[C@H]1C[C@@H](C2)C3)C(=O)O)C(=O)O | C77H18O6 |  | 5801,678 |
| 47 | 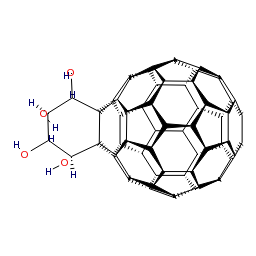 | c12[C@@H]3[C@H]4[C@H]5[C@H]6[C@H]7[C@@H]3c3c2c2c8c9c1[C@@H]1[C@@H]%10c9c9c%11c8[C@]8%12c2c2c3[C@H]3c%13c2c2[C@@]8(c8c%11c%11[C@@H]9[C@H]9[C@@H]%10[C@H]%10[C@@H]([C@H]41)[C@@H]5[C@@H]1[C@@H]4[C@H]%10[C@H]9[C@@H]5c%11c9c%10[C@@H]5[C@@H]4[C@H]4[C@@H]1[C@@H]6[C@@H]([C@@H]73)[C@H]1c%13c(c%10[C@@H]41)c2c89)[C@@H]([C@@H]([C@@H]([C@@H]%12O)O)O)O | C64H66O4 | S | 4787,074 |
| 48 | 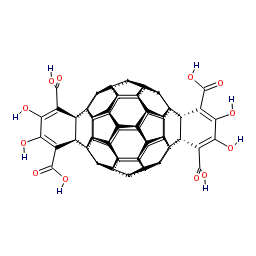 | c12c3c4c5c6c7c3c3c2c2c8c9c1c1c%10c9c9c%11c8[C@]8%12c2c2c3[C@H]3c%13c2c2[C@@]8(c8c%11c%11[C@@H]9[C@H]9c%10c%10c(c41)[C@@]15[C@@]4(c5c%10c9c9c%11c%10c%11c9c5c5c4c6c([C@@H]73)c3c%13c(c%11c53)c2c8%10)C(=C(C(=C1C(=O)O)O)O)C(=O)O)C(=C(C(=C%12C(=O)O)O)O)C(=O)O.[H].[H] | C72H64O12 | S | 5466,824 |
| 49 | 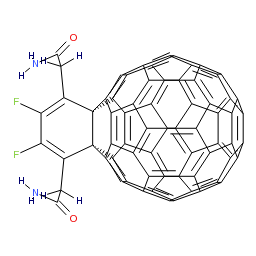 | c12[C@@H]3[C@H]4[C@H]5[C@H]6[C@H]7[C@@H]3c3c2c2c8c9c1[C@@H]1[C@@H]%10c9c9c%11c8[C@]8%12c2c2c3[C@H]3c%13c2c2[C@@]8(c8c%11c%11[C@@H]9[C@H]9[C@@H]%10[C@H]%10[C@@H]([C@H]41)[C@@H]5[C@@H]1[C@@H]4[C@H]%10[C@H]9[C@@H]5c%11c9c%10[C@@H]5[C@@H]4[C@H]4[C@@H]1[C@@H]6[C@@H]([C@@H]73)[C@H]1c%13c(c%10[C@@H]41)c2c89)C(=C(C(=C%12CC(=O)N)F)F)CC(=O)N | C68H10N2O2F2 |  | 5035,701 |
| 50 | 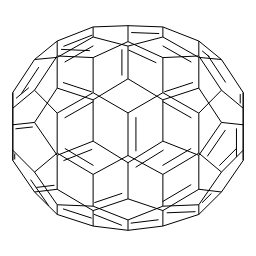 | [C@H]12[C@@H]3[C@H]4[C@H]5[C@H]6[C@H]7[C@@H]3[C@H]3[C@@H]2[C@H]2[C@H]8[C@H]9[C@@H]1[C@@H]1[C@@H]%10[C@H]9[C@@H]9[C@@H]%11[C@H]8[C@@H]8[C@H]2[C@@H]2[C@H]3[C@H]3[C@H]%12[C@@H]2[C@H]2[C@@H]8[C@H]8[C@@H]%11[C@H]%11[C@@H]9[C@H]9[C@@H]%10[C@H]%10[C@@H]([C@H]41)[C@@H]5[C@@H]1[C@@H]4[C@H]%10[C@H]9[C@@H]5[C@H]%11[C@H]9[C@H]%10[C@@H]5[C@@H]4[C@H]4[C@@H]1[C@@H]6[C@@H]([C@@H]73)[C@H]1[C@@H]%12[C@H]([C@@H]%10[C@@H]41)[C@@H]2[C@@H]89 | C70 |  | 4224,303 |
| 51 | 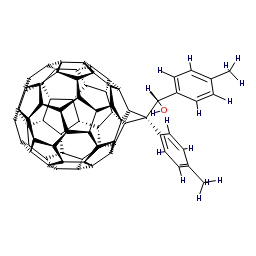 | c1c(ccc(c1)[C@@]1([C@]23c4c5c6c7c8c9c%10c%11c%12c%13c%14[C@H]%15[C@H]%16[C@H]%17[C@@H]%13[C@@H]%11[C@H]%11[C@H]%13[C@@H]%10[C@@H]8[C@H]8[C@H]%10[C@@H]7c5c5c7c4c4c3c(c%14c3c%12c9c6[C@]123)c%15c1c4c2c7[C@H]3[C@@H]4[C@@H]6[C@H]([C@@H]%10[C@@H]53)[C@H]8[C@H]%13[C@H]3[C@@H]6[C@@H]5[C@H]([C@@H]%17[C@@H]%113)[C@H]%16[C@H]1[C@H]2[C@H]45)[C@H](c1ccc(cc1)C)O)C | C76H74O | S | 5948,222 |
| 52 | 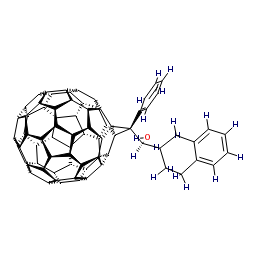 | c1cccc(c1)[C@@]1([C@]23c4c5c6c7c8c9c%10c%11c%12c%13c%14[C@H]%15[C@H]%16[C@H]%17[C@@H]%13[C@@H]%11[C@H]%11[C@H]%13[C@@H]%10[C@@H]8[C@H]8[C@H]%10[C@@H]7c5c5c7c4c4c3c(c%14c3c%12c9c6[C@]123)c%15c1c4[C@@H]2[C@H]7[C@H]3[C@@H]4[C@@H]6[C@H]([C@@H]%10[C@@H]53)[C@H]8[C@H]%13[C@H]3[C@@H]6[C@@H]5[C@H]([C@@H]%17[C@@H]%113)[C@H]%16[C@H]1[C@H]2[C@H]45)[C@H]([C@H]1CCc2c(C1)cccc2)O | C78H76O | S | 6047,871 |
| 53 | 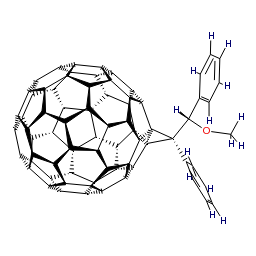 | c1cccc(c1)[C@@]1([C@]23c4c5c6c7c8c9c%10c%11c%12c%13c%14[C@H]%15[C@H]%16[C@H]%17[C@@H]%13[C@@H]%11[C@H]%11[C@H]%13[C@@H]%10[C@@H]8[C@H]8[C@H]%10[C@@H]7c5c5c7c4c4c3c(c%14c3c%12c9c6[C@]123)c%15c1c4c2c7[C@H]3[C@@H]4[C@@H]6[C@H]([C@@H]%10[C@@H]53)[C@H]8[C@H]%13[C@H]3[C@@H]6[C@@H]5[C@H]([C@@H]%17[C@@H]%113)[C@H]%16[C@H]1[C@H]2[C@H]45)[C@H](c1ccccc1)OC | C75H72O | S | 5630,842 |
| 54 | 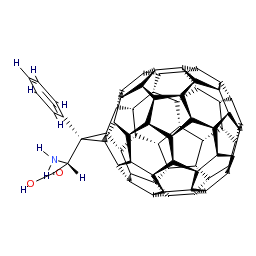 | c1cccc(c1)[C@@]1([C@]23c4c5c6c7c8c9[C@@H]%10[C@@H]%11c%12c%13c%14[C@H]%15[C@H]%16[C@H]%17[C@@H]%13[C@@H]%11[C@H]%11[C@H]%13[C@@H]%10[C@@H]8[C@H]8[C@H]%10[C@@H]7c5c5c7c4c4c3c(c%14c3c%12c9c6[C@]123)c%15c1c4[C@@H]2[C@H]7[C@H]3[C@@H]4[C@@H]6[C@H]([C@@H]%10[C@@H]53)[C@H]8[C@H]%13[C@H]3[C@@H]6[C@@H]5[C@H]([C@@H]%17[C@@H]%113)[C@H]%16[C@H]1[C@H]2[C@H]45)[C@@H](N)C(=O)O | C69H67NO2 | S | 5165,049 |
| 55 | 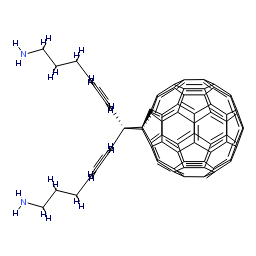 | c1c(ccc(c1)C1([C@]23c4c5c6c7c8c9[C@@H]%10[C@@H]%11c%12c%13c%14[C@H]%15[C@H]%16[C@H]%17[C@@H]%13[C@@H]%11[C@H]%11[C@H]%13[C@@H]%10[C@@H]8[C@H]8[C@H]%10[C@@H]7c5c5c7c4c4c3c(c%14c3c%12c9c6[C@]123)c%15c1c4[C@@H]2[C@H]7[C@H]3[C@@H]4[C@@H]6[C@H]([C@@H]%10[C@@H]53)[C@H]8[C@H]%13[C@H]3[C@@H]6[C@@H]5[C@H]([C@@H]%17[C@@H]%113)[C@H]%16[C@H]1[C@H]2[C@H]45)c1ccc(cc1)CCCN)CCCN | C79H24N2 |  | 6456,124 |
| 56 | 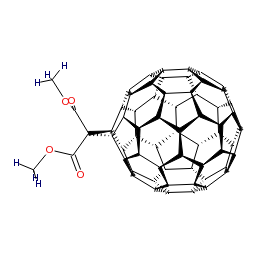 | C(=O)(C1([C@]23c4c5c6c7c8c9c%10c%11c%12c%13c%14C%15=C%16[C@H]%17[C@@H]%13[C@@H]%11[C@H]%11[C@H]%13[C@@H]%10[C@@H]8[C@H]8[C@H]%10[C@@H]7c5c5c7c4c4c3c(c%14c3c%12c9c6[C@]123)c%15c1c4c2c7[C@H]3[C@@H]4[C@@H]6[C@H]([C@@H]%10[C@@H]53)[C@H]8[C@H]%13[C@H]3[C@@H]6[C@@H]5[C@H]([C@@H]%17[C@@H]%113)[C@H]%16[C@H]1[C@H]2[C@H]45)C(=O)OC)OC | C65H64O4 | S | 5084,902 |
| 57 | 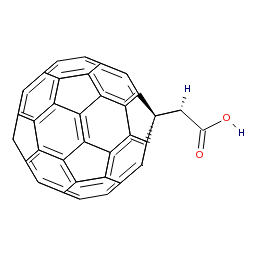 | c12c3c4[C@H]5[C@H]6[C@H]7[C@@H]3[C@H]3c2c2c8c9c1c1[C@]%10%11c9c9c%12c8[C@@H]8[C@H]2[C@@H]2[C@H]3[C@H]3[C@H]%13[C@@H]2[C@H]2[C@@H]8[C@H]8[C@@H]%12c%12c9c9[C@@]%10(c%10c(c41)c5c1c4c%10c9c5c%12[C@H]9[C@H]%10c5c4[C@H]4[C@@H]1[C@@H]6[C@@H]([C@@H]73)[C@H]1[C@@H]%13[C@H]([C@@H]%10[C@@H]41)[C@@H]2[C@@H]89)[C@H]%11C(=O)O | C62H2O2 | ~~S~~ | 4412,666 |
| 58 | 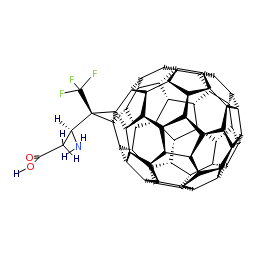 | c12c3c4C5=C6[C@H]7[C@@H]3[C@H]3c2c2c8c9c1c1[C@]%10%11c9c9c%12c8[C@@H]8[C@H]2[C@@H]2[C@H]3[C@H]3[C@H]%13[C@@H]2[C@H]2[C@@H]8C8=C%12c%12c9c9[C@@]%10(c%10c(c41)c5c1c4c%10c9c5c%12[C@H]9[C@H]%10c5c4[C@H]4[C@@H]1[C@@H]6[C@@H]([C@@H]73)[C@H]1[C@@H]%13[C@H]([C@@H]%10[C@@H]41)[C@@H]2[C@@H]89)[C@@]%11([C@@H](N)CC(=O)O)C(F)(F)F | C65H64NO2F3 | S | 5077,253 |
| 59 | 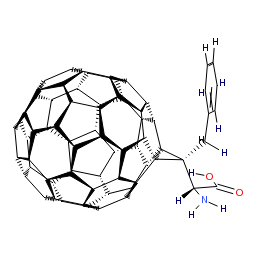 | c12c3c4[C@H]5[C@H]6[C@H]7[C@@H]3[C@H]3[C@@H]2[C@H]2c8c9c1c1[C@]%10%11c9c9c%12c8[C@@H]8[C@H]2[C@@H]2[C@H]3[C@H]3[C@H]%13[C@@H]2[C@H]2[C@@H]8[C@H]8[C@@H]%12c%12c9c9[C@@]%10(c%10c(c41)c5c1c4c%10c9c5c%12[C@H]9[C@H]%10c5c4[C@H]4[C@@H]1[C@@H]6[C@@H]([C@@H]73)[C@H]1[C@@H]%13[C@H]([C@@H]%10[C@@H]41)[C@@H]2[C@@H]89)[C@@]%11(Cc1ccccc1)[C@H](C(=O)O)N | C70H69NO2 | S | 5165,269 |
| 60 | 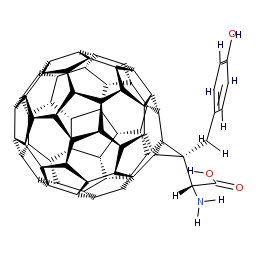 | [C@H]12[C@@H]3[C@H]4[C@H]5[C@H]6[C@H]7[C@@H]3[C@H]3[C@@H]2[C@H]2[C@H]8[C@H]9[C@@H]1C1=C4C4=C%10[C@@]%11%12C%13=C%14C(=C9[C@@]1%11[C@]%12(C[C@@H]1CC[C@H](CC1)O)[C@H](C(=O)O)N)[C@@H]1[C@H]8[C@@H]8[C@H]2[C@@H]2[C@H]3[C@H]3[C@H]9[C@@H]2[C@H]2[C@@H]8[C@H]8[C@@H]1[C@@H]%14[C@H]1[C@H]%11[C@@H]%13[C@@H]%10[C@H]%10[C@@H]([C@H]54)[C@@H]4[C@@H]5C%10=C%11[C@H]%10[C@@H]%11[C@H]([C@@H]2[C@@H]8[C@@H]1%10)[C@H]9[C@H]([C@H]([C@@H]73)[C@H]64)[C@H]5%11 | C70H69NO3 | S | 5248,05 |
| 61 | 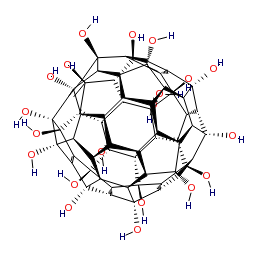 | [C@@]12(C3=C4[C@@]5([C@@]6(C7=C8C9=C([C@@]37O)[C@@]3(C1=C1[C@@]7(C%10=C2[C@]4(C2=C4C%11=C%12C%13=C%14[C@]%15(C%16=C%17C(=C7[C@@]7(C%10=C2[C@]%11(C%14=C%167)O)O)[C@]2(C1=C3[C@@]1(C3=C2[C@]%17(C2=C%15C7=C%10[C@@]2(C3=C([C@]91O)C1=C8[C@@]2(C(=C([C@@]%137O)[C@]%12(C(=C62)[C@@]54O)O)[C@@]%101O)O)O)O)O)O)O)O)O)O)O)O)O | C60H54O20 | S | 4725,108 |
| 62 | 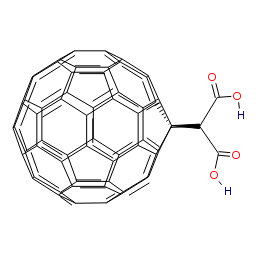 | c12c3c4[C@H]5[C@H]6[C@H]7[C@@H]3[C@H]3c2c2c8c9c1c1[C@]%10%11c9c9c%12c8[C@@H]8[C@H]2[C@@H]2[C@H]3[C@H]3[C@H]%13[C@@H]2[C@H]2[C@@H]8[C@H]8[C@@H]%12c%12c9c9[C@@]%10(c%10c(c41)c5c1c4c%10c9c5c%12[C@H]9[C@H]%10[C@@H]5[C@@H]4[C@H]4[C@@H]1[C@@H]6[C@@H]([C@@H]73)[C@H]1[C@@H]%13[C@H]([C@@H]%10[C@@H]41)[C@@H]2[C@@H]89)C%11(C(=O)O)C(=O)O | C63H2O4 |  | 4548,741 |
| 63 | 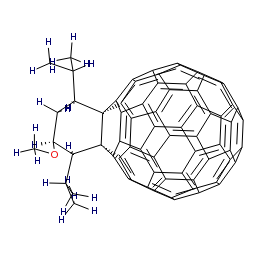 | c12c3c4[C@H]5[C@H]6[C@H]7[C@@H]3[C@H]3c2c2c8c9c1c1[C@H]%10c9c9c%11c8[C@@H]8[C@H]2[C@@H]2[C@H]3[C@H]3[C@H]%12[C@@H]2[C@H]2[C@@H]8[C@H]8[C@@H]%11c%11c9c9[C@@H](c%13c(c41)c5c1c4c%13c9c5c%11[C@H]9[C@H]%11c5c4[C@H]4[C@@H]1[C@@H]6[C@@H]([C@@H]73)[C@H]1[C@@H]%12[C@H]([C@@H]%11[C@@H]41)[C@@H]2[C@@H]89)[C@@H](C[C@@H]([C@@H]%10C(C)C)OC)C(C)C | C71H22O |  | 5334,263 |
| 64 | 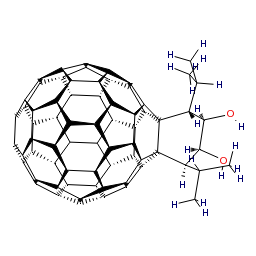 | c12c3c4[C@H]5[C@H]6[C@H]7[C@@H]3[C@H]3c2c2c8c9c1c1[C@]%10%11c9c9c%12c8[C@@H]8[C@H]2[C@@H]2[C@H]3[C@H]3[C@H]%13[C@@H]2[C@H]2[C@@H]8[C@H]8[C@@H]%12c%12c9c9[C@@]%10(c%10c(c41)c5c1c4c%10c9c5c%12[C@H]9[C@H]%10[C@@H]5[C@@H]4[C@H]4[C@@H]1[C@@H]6[C@@H]([C@@H]73)[C@H]1[C@@H]%13[C@H]([C@@H]%10[C@@H]41)[C@@H]2[C@@H]89)[C@@H]([C@@H]([C@@H]([C@H]%11C(C)C)O)O)C(C)C | C70H78O2 | S | 5280,274 |
| 65 | 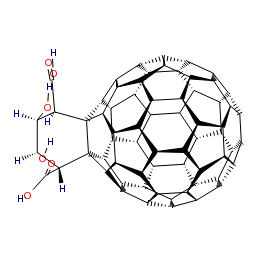 | c12c3c4[C@H]5[C@H]6[C@H]7[C@@H]3[C@H]3c2c2c8c9c1c1[C@]%10%11c9c9c%12c8C8=C%13[C@H]%14[C@@H]%12c%12c9c9[C@@]%10(c%10c(c41)c5c1c4c%10c9c5c%12[C@H]9[C@H]%10c5c4[C@H]4C1=C6C1=C5[C@@H]6[C@H]([C@@H]%10[C@@H]45)[C@H]([C@@H]%149)C%13=C4[C@H]([C@@H]28)[C@H]3[C@@H]([C@H]64)[C@H]71)[C@@H]([C@@H]([C@@H]([C@H]%11C(=O)O)O)O)C(=O)O | C66H66O6 | S | 4866,106 |
| 66 | 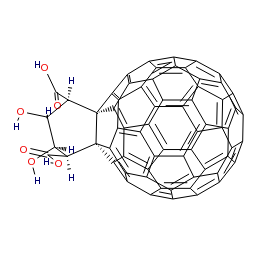 | c12c3c4[C@H]5[C@H]6[C@H]7[C@@H]3[C@H]3c2c2c8c9c1c1[C@]%10%11c9c9c%12c8[C@@H]8[C@H]2[C@@H]2[C@H]3[C@H]3[C@H]%13[C@@H]2[C@H]2[C@@H]8[C@H]8[C@@H]%12c%12c9c9[C@@]%10(c%10c(c41)c5c1c4c%10c9c5c%12[C@H]9[C@H]%10c5c4[C@H]4[C@@H]1[C@@H]6[C@@H]([C@@H]73)[C@H]1[C@@H]%13[C@H]([C@@H]%10[C@@H]41)[C@@H]2[C@@H]89)[C@H]([C@H]([C@H]([C@H]%11C(=O)O)O)O)C(=O)O | C66H10O6 |  | 4972,602 |
| 67 | 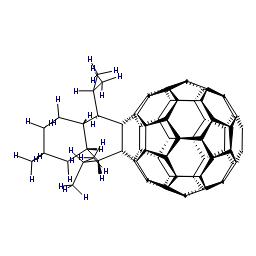 | c12c3c4[C@H]5[C@H]6[C@H]7[C@@H]3[C@H]3c2c2c8c9c1c1[C@]%10%11c9c9c%12c8[C@@H]8[C@H]2[C@@H]2[C@H]3[C@H]3[C@H]%13[C@@H]2[C@H]2[C@@H]8[C@H]8[C@@H]%12c%12c9c9[C@@]%10(c%10c(c41)c5c1c4c%10c9c5c%12[C@H]9[C@H]%10c5c4[C@H]4[C@@H]1[C@@H]6[C@@H]([C@@H]73)[C@H]1[C@@H]%13[C@H]([C@@H]%10[C@@H]41)[C@@H]2[C@@H]89)[C@@H]([C@@H]1[C@H]([C@H]%11C(C)C)C[C@@H](CC1)C)C(C)C | C75H86 | S | 5551,363 |
| 68 | 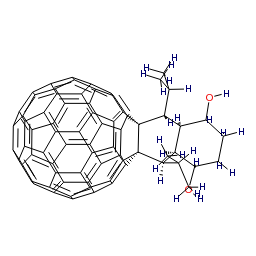 | c12c3c4[C@H]5[C@H]6[C@H]7[C@@H]3[C@H]3c2c2c8c9c1c1[C@]%10%11c9c9c%12c8[C@@H]8[C@H]2[C@@H]2[C@H]3[C@H]3[C@H]%13[C@@H]2[C@H]2[C@@H]8[C@H]8[C@@H]%12c%12c9c9[C@@]%10(c%10c(c41)c5c1c4c%10c9c5c%12[C@H]9[C@H]%10c5c4[C@H]4[C@@H]1[C@@H]6[C@@H]([C@@H]73)[C@H]1[C@@H]%13[C@H]([C@@H]%10[C@@H]41)[C@@H]2[C@@H]89)[C@@H]([C@@H]1[C@H]([C@H]%11C(C)C)[C@@H](CC[C@H]1O)O)C(C)C | C74H26O2 |  | 5476,709 |
| 69 | 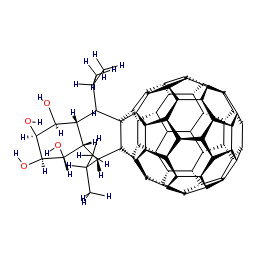 | c12c3c4[C@H]5[C@H]6[C@H]7[C@@H]3[C@H]3[C@@H]2[C@H]2c8c9c1c1[C@]%10%11c9c9c%12c8[C@@H]8[C@H]2[C@@H]2[C@H]3[C@H]3[C@H]%13[C@@H]2[C@H]2[C@@H]8[C@H]8[C@@H]%12c%12c9c9[C@@]%10(c%10c(c41)c5c1c4c%10c9c5c%12[C@H]9[C@H]%10c5c4[C@H]4[C@@H]1[C@@H]6[C@@H]([C@@H]73)[C@H]1[C@@H]%13[C@H]([C@@H]%10[C@@H]41)[C@@H]2[C@@H]89)[C@@H]([C@@H]1[C@H]([C@H]%11C(C)C)[C@H]([C@H]([C@H]([C@H]1O)O)O)O)C(C)C | C74H84O4 | S | 5560,537 |
| 70 | 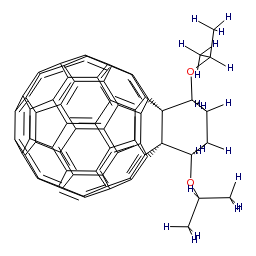 | c12c3c4[C@H]5[C@H]6[C@H]7[C@@H]3[C@H]3[C@@H]2[C@H]2c8c9c1c1[C@]%10%11c9c9c%12c8[C@@H]8[C@H]2[C@@H]2[C@H]3[C@H]3[C@H]%13[C@@H]2[C@H]2[C@@H]8[C@H]8[C@@H]%12c%12c9c9[C@@]%10(c%10c(c41)c5c1c4c%10c9c5c%12[C@H]9[C@H]%10[C@@H]5[C@@H]4[C@H]4[C@@H]1[C@@H]6[C@@H]([C@@H]73)[C@H]1[C@@H]%13[C@H]([C@@H]%10[C@@H]41)[C@@H]2[C@@H]89)[C@@H](CC[C@@H]%11OC(C)C)OC(C)C | C70H20O2 |  | 5431,246 |
| 71 | 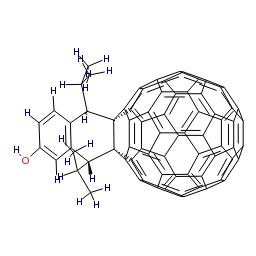 | c12c3c4[C@H]5[C@H]6[C@H]7[C@@H]3[C@H]3[C@@H]2[C@H]2c8c9c1c1[C@]%10%11c9c9c%12c8[C@@H]8[C@H]2[C@@H]2[C@H]3[C@H]3[C@H]%13[C@@H]2[C@H]2[C@@H]8[C@H]8[C@@H]%12c%12c9c9[C@@]%10(c%10c(c41)c5c1c4c%10c9c5c%12[C@H]9[C@H]%10c5c4[C@H]4[C@@H]1[C@@H]6[C@@H]([C@@H]73)[C@H]1[C@@H]%13[C@H]([C@@H]%10[C@@H]41)[C@@H]2[C@@H]89)[C@@H](c1c([C@H]%11C(C)C)cc(cc1)O)C(C)C | C74H20O |  | 5326,321 |
| 72 | 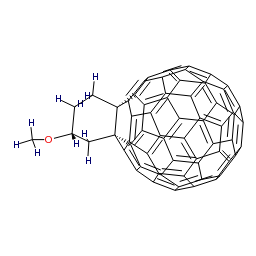 | c12c3c4[C@H]5[C@H]6[C@H]7[C@@H]3[C@H]3[C@@H]2[C@H]2c8c9c1c1[C@]%10%11c9c9c%12c8[C@@H]8[C@H]2[C@@H]2[C@H]3[C@H]3[C@H]%13[C@@H]2[C@H]2[C@@H]8[C@H]8[C@@H]%12c%12c9c9[C@@]%10(c%10c(c41)c5c1c4c%10c9c5c%12[C@H]9[C@H]%10[C@@H]5[C@@H]4[C@H]4[C@@H]1[C@@H]6[C@@H]([C@@H]73)[C@H]1[C@@H]%13[C@H]([C@@H]%10[C@@H]41)[C@@H]2[C@@H]89)CC[C@H](C%11)OC | C65H10O |  | 4900,983 |
| 73 | 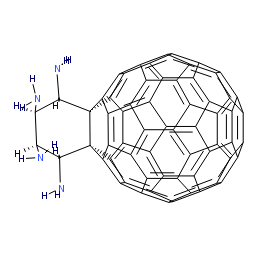 | c12c3c4[C@H]5[C@H]6[C@H]7[C@@H]3[C@H]3c2c2c8c9c1c1[C@]%10%11c9c9c%12c8[C@@H]8[C@H]2[C@@H]2[C@H]3[C@H]3[C@H]%13[C@@H]2[C@H]2[C@@H]8[C@H]8[C@@H]%12c%12c9c9[C@@]%10(c%10c(c41)c5c1c4c%10c9c5c%12[C@H]9[C@H]%10c5c4[C@H]4[C@@H]1[C@@H]6[C@@H]([C@@H]73)[C@H]1[C@@H]%13[C@H]([C@@H]%10[C@@H]41)[C@@H]2[C@@H]89)[C@@H]([C@@H]([C@@H]([C@@H]%11N)N)N)N | C64H12N4 |  | 4777,545 |
| 74 | 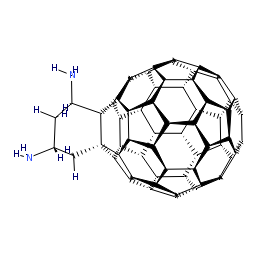 | c12c3c4[C@H]5[C@H]6[C@H]7[C@@H]3[C@H]3c2c2c8c9c1c1[C@]%10%11c9c9c%12c8[C@@H]8[C@H]2[C@@H]2[C@H]3[C@H]3[C@H]%13[C@@H]2[C@H]2[C@@H]8[C@H]8[C@@H]%12c%12c9c9[C@@]%10(c%10c(c41)c5c1c4c%10c9c5c%12[C@H]9[C@H]%10c5c4[C@H]4[C@@H]1[C@@H]6[C@@H]([C@@H]73)[C@H]1[C@@H]%13[C@H]([C@@H]%10[C@@H]41)[C@@H]2[C@@H]89)[C@@H](C[C@H](C%11)N)N | C64H68N2 | S | 4750,906 |
| 75 | 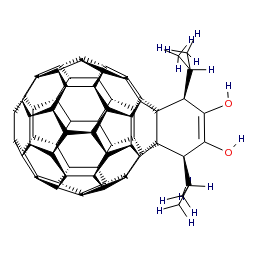 | c12c3c4[C@H]5[C@H]6[C@H]7[C@@H]3[C@H]3c2c2c8c9c1c1[C@H]%10c9c9c%11c8[C@@H]8[C@H]2[C@@H]2[C@H]3[C@H]3[C@H]%12[C@@H]2[C@H]2[C@@H]8[C@H]8[C@@H]%11c%11c9c9[C@@H](c%13c(c41)c5c1c4c%13c9c5c%11[C@H]9[C@H]%11c5c4[C@H]4[C@@H]1[C@@H]6[C@@H]([C@@H]73)[C@H]1[C@@H]%12[C@H]([C@@H]%11[C@@H]41)[C@@H]2[C@@H]89)/C(=C(\C(=C/%10\C(C)C)\O)/O)/C(C)C | C70H76O2 | S | 5241,764 |
| 76 | 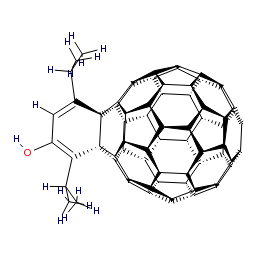 | c12c3c4[C@H]5[C@H]6[C@H]7[C@@H]3[C@H]3c2c2c8c9c1c1[C@H]%10c9c9c%11c8[C@@H]8[C@H]2[C@@H]2[C@H]3[C@H]3[C@H]%12[C@@H]2[C@H]2[C@@H]8[C@H]8[C@@H]%11c%11c9c9[C@@H](c%13c(c41)c5c1c4c%13c9c5c%11[C@H]9[C@H]%11c5c4[C@H]4[C@@H]1[C@@H]6[C@@H]([C@@H]73)[C@H]1[C@@H]%12[C@H]([C@@H]%11[C@@H]41)[C@@H]2[C@@H]89)/C(=C\C(=C/%10\C(C)C)\O)/C(C)C | C70H74O | S | 5087,586 |
| 77 | 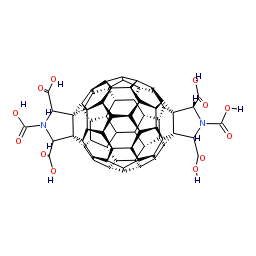 | c12c3c4[C@H]5[C@H]6c7c3c3c2c2c8c9c1c1[C@]%10%11c9c9c%12c8c8c2c2c3c3[C@@]%13%14c2c2c8[C@H]8[C@@H]%12c%12c9c9[C@@]%10(c%10c(c41)c5c1c4c%10c9c5c%12c9c%10c5c4c4c1c6c(c73)c1[C@@]%13(c(c%10c41)c2c89)[C@H](N([C@H]%14C(=O)O)C(=O)O)C(=O)O)[C@@H](N([C@H]%11C(=O)O)C(=O)O)C(=O)O | C70H66N2O12 | S | 5490,023 |
| 78 | 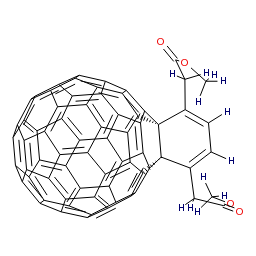 | c12c3c4[C@H]5[C@H]6[C@H]7[C@@H]3[C@H]3c2c2c8c9c1c1[C@]%10%11c9c9c%12c8[C@@H]8[C@H]2[C@@H]2[C@H]3[C@H]3[C@H]%13[C@@H]2[C@H]2[C@@H]8[C@H]8[C@@H]%12c%12c9c9[C@@]%10(c%10c(c41)c5c1c4c%10c9c5c%12[C@H]9[C@H]%10c5c4[C@H]4[C@@H]1[C@@H]6[C@@H]([C@@H]73)[C@H]1[C@@H]%13[C@H]([C@@H]%10[C@@H]41)[C@@H]2[C@@H]89)C(=CC=C%11CC(=O)OC)CC(=O)OC | C70H12O4 |  | 5196,602 |
| 79 | 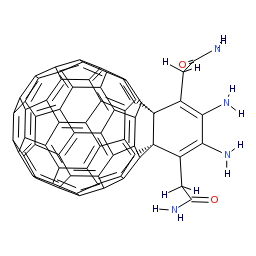 | c12c3c4[C@H]5[C@H]6[C@H]7[C@@H]3[C@H]3c2c2c8c9c1c1[C@]%10%11c9c9c%12c8[C@@H]8[C@H]2[C@@H]2[C@H]3[C@H]3[C@H]%13[C@@H]2[C@H]2[C@@H]8[C@H]8[C@@H]%12c%12c9c9[C@@]%10(c%10c(c41)c5c1c4c%10c9c5c%12[C@H]9[C@H]%10c5c4[C@H]4[C@@H]1[C@@H]6[C@@H]([C@@H]73)[C@H]1[C@@H]%13[C@H]([C@@H]%10[C@@H]41)[C@@H]2[C@@H]89)C(=C(C(=C%11CC(=O)N)N)N)CC(=O)N | C68H12N4O2 |  | 5213,619 |
| 80 | 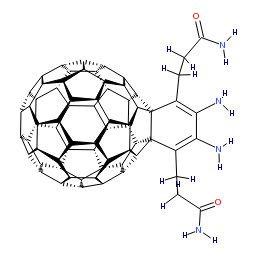 | c12c3c4[C@H]5[C@H]6[C@H]7[C@@H]3[C@H]3c2c2c8c9c1c1[C@]%10%11c9c9c%12c8[C@@H]8[C@H]2[C@@H]2[C@H]3[C@H]3[C@H]%13[C@@H]2[C@H]2[C@@H]8[C@H]8[C@@H]%12c%12c9c9[C@@]%10(c%10c(c41)c5c1c4c%10c9c5c%12[C@H]9[C@H]%10c5c4[C@H]4[C@@H]1[C@@H]6[C@@H]([C@@H]73)[C@H]1[C@@H]%13[C@H]([C@@H]%10[C@@H]41)[C@@H]2[C@@H]89)C(=C(C(=C%11CCC(=O)N)N)N)CCC(=O)N | C70H74N4O2 | S | 5634,405 |
| 81 | 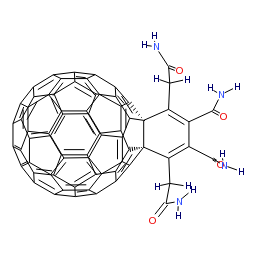 | c12c3c4[C@H]5[C@H]6[C@H]7[C@@H]3[C@H]3c2c2c8c9c1c1[C@]%10%11c9c9c%12c8[C@@H]8[C@H]2[C@@H]2[C@H]3[C@H]3[C@H]%13[C@@H]2[C@H]2[C@@H]8[C@H]8[C@@H]%12c%12c9c9[C@@]%10(c%10c(c41)c5c1c4c%10c9c5c%12[C@H]9[C@H]%10c5c4[C@H]4[C@@H]1[C@@H]6[C@@H]([C@@H]73)[C@H]1[C@@H]%13[C@H]([C@@H]%10[C@@H]41)[C@@H]2[C@@H]89)C(=C(C(=C%11CC(=O)N)C(=O)N)C(=O)N)CC(=O)N | C70H14N4O4 |  | 5470,886 |
| 82 | 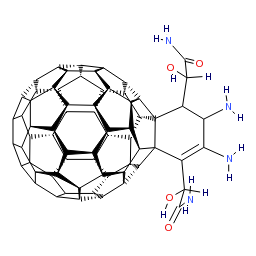 | [C@H]12[C@@H]3[C@H]4[C@H]5[C@H]6[C@H]7[C@@H]3[C@H]3C2=C2[C@H]8[C@H]9[C@@H]1C1=C4C4=C%10[C@@H]%11C%12=C%13C(=C9[C@H]1/C(=C(\C(=C/%11\[C@@H](C(=O)N)O)\N)/N)/[C@@H](C(=O)N)O)[C@@H]1[C@H]8[C@@H]8[C@H]2[C@@H]2[C@H]3[C@H]3[C@H]9[C@@H]2[C@H]2[C@@H]8[C@H]8[C@@H]1[C@@H]%13[C@H]1[C@H]%11[C@@H]%12[C@@H]%10[C@H]%10[C@@H]([C@H]54)[C@@H]4[C@@H]5C%10=C%11[C@H]%10[C@@H]%11[C@H]([C@@H]2[C@@H]8[C@@H]1%10)[C@H]9[C@H]([C@H]([C@@H]73)[C@H]64)[C@H]5%11 | C68H72N4O4 | S | 5284,038 |
| 83 | 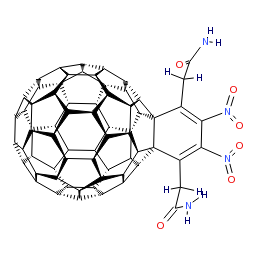 | c12c3c4[C@H]5[C@H]6[C@H]7[C@@H]3[C@H]3c2c2c8c9c1c1[C@]%10%11c9c9c%12c8[C@@H]8[C@H]2[C@@H]2[C@H]3[C@H]3[C@H]%13[C@@H]2[C@H]2[C@@H]8[C@H]8[C@@H]%12c%12c9c9[C@@]%10(c%10c(c41)c5c1c4c%10c9c5c%12[C@H]9[C@H]%10c5c4[C@H]4[C@@H]1[C@@H]6[C@@H]([C@@H]73)[C@H]1[C@@H]%13[C@H]([C@@H]%10[C@@H]41)[C@@H]2[C@@H]89)C(=C(C(=C%11CC(=O)N)N(=O)=O)N(=O)=O)CC(=O)N | C68H66N4O6 | S | 5478,564 |
| 84 | 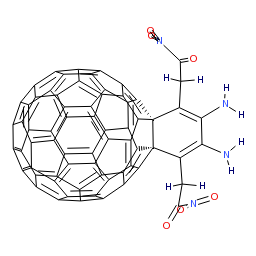 | c12c3c4[C@H]5[C@H]6[C@H]7[C@@H]3[C@H]3c2c2c8c9c1c1[C@]%10%11c9c9c%12c8[C@@H]8[C@H]2[C@@H]2[C@H]3[C@H]3[C@H]%13[C@@H]2[C@H]2[C@@H]8[C@H]8[C@@H]%12c%12c9c9[C@@]%10(c%10c(c41)c5c1c4c%10c9c5c%12[C@H]9[C@H]%10c5c4[C@H]4[C@@H]1[C@@H]6[C@@H]([C@@H]73)[C@H]1[C@@H]%13[C@H]([C@@H]%10[C@@H]41)[C@@H]2[C@@H]89)C(=C(C(=C%11CC=O)N)N)CC(=O)N(=O)=O.[N](=O)=O | C68H10N4O6 |  | 5328,48 |
| 85 | 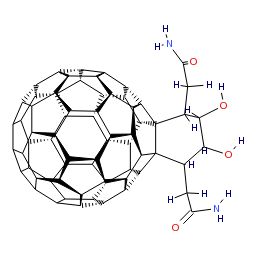 | c12c3c4[C@H]5[C@H]6[C@H]7[C@@H]3[C@H]3c2c2c8c9c1c1[C@]%10%11c9c9c%12c8[C@@H]8[C@H]2[C@@H]2[C@H]3[C@H]3[C@H]%13[C@@H]2[C@H]2[C@@H]8[C@H]8[C@@H]%12c%12c9c9[C@@]%10(c%10c(c41)c5c1c4c%10c9c5c%12[C@H]9[C@H]%10c5c4[C@H]4[C@@H]1[C@@H]6[C@@H]([C@@H]73)[C@H]1[C@@H]%13[C@H]([C@@H]%10[C@@H]41)[C@@H]2[C@@H]89)[C@H]([C@@H]([C@@H]([C@H]%11CC(=O)N)O)O)CC(=O)N | C68H72N2O4 | S | 5249,234 |
| 86 | 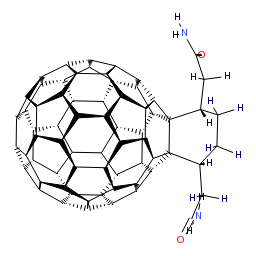 | c12c3c4[C@H]5[C@H]6[C@H]7[C@@H]3[C@H]3c2c2c8c9c1c1[C@]%10%11c9c9c%12c8[C@@H]8[C@H]2[C@@H]2[C@H]3[C@H]3[C@H]%13[C@@H]2[C@H]2[C@@H]8[C@H]8[C@@H]%12c%12c9c9[C@@]%10(c%10c(c41)c5c1c4c%10c9c5c%12[C@H]9[C@H]%10c5c4[C@H]4[C@@H]1[C@@H]6[C@@H]([C@@H]73)[C@H]1[C@@H]%13[C@H]([C@@H]%10[C@@H]41)[C@@H]2[C@@H]89)[C@H](CC[C@H]%11CC(=O)N)CC(=O)N | C68H72N2O2 | S | 4896,138 |
| 87 | 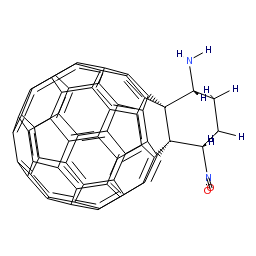 | c12c3c4[C@H]5[C@H]6[C@H]7[C@@H]3[C@H]3[C@@H]2[C@H]2c8c9c1c1[C@]%10%11c9c9c%12c8[C@@H]8[C@H]2[C@@H]2[C@H]3[C@H]3[C@H]%13[C@@H]2[C@H]2[C@@H]8[C@H]8[C@@H]%12c%12c9c9[C@@]%10(c%10c(c41)c5c1c4c%10c9c5c%12[C@H]9[C@H]%10[C@@H]5[C@@H]4[C@H]4[C@@H]1[C@@H]6[C@@H]([C@@H]73)[C@H]1[C@@H]%13[C@H]([C@@H]%10[C@@H]41)[C@@H]2[C@@H]89)[C@H](CC[C@H]%11N)N(=O)=O | C64H8N2O2 |  | 4670,893 |
| 88 | 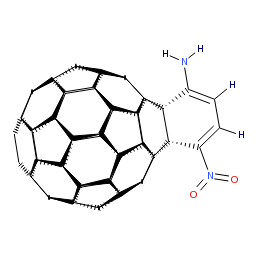 | c12c3c4[C@H]5[C@H]6[C@H]7[C@@H]3[C@H]3c2c2c8c9c1c1[C@H]%10c9c9c%11c8[C@@H]8[C@H]2[C@@H]2[C@H]3[C@H]3[C@H]%12[C@@H]2[C@H]2[C@@H]8[C@H]8[C@@H]%11c%11c9c9[C@@H](c%13c(c41)c5c1c4c%13c9c5c%11[C@H]9[C@H]%11c5c4[C@H]4[C@@H]1[C@@H]6[C@@H]([C@@H]73)[C@H]1[C@@H]%12[C@H]([C@@H]%11[C@@H]41)[C@@H]2[C@@H]89)/C(=C\C=C/%10\N)/N(=O)=O | C64H62N2O2 | S | 4764,881 |
| 89 | 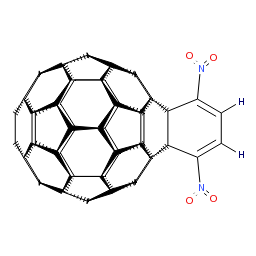 | c12c3c4[C@H]5[C@H]6[C@H]7[C@@H]3[C@H]3c2c2c8c9c1c1[C@]%10%11c9c9c%12c8[C@@H]8[C@H]2[C@@H]2[C@H]3[C@H]3[C@H]%13[C@@H]2[C@H]2[C@@H]8[C@H]8[C@@H]%12c%12c9c9[C@@]%10(c%10c(c41)c5c1c4c%10c9c5c%12[C@H]9[C@H]%10c5c4[C@H]4[C@@H]1[C@@H]6[C@@H]([C@@H]73)[C@H]1[C@@H]%13[C@H]([C@@H]%10[C@@H]41)[C@@H]2[C@@H]89)C(=CC=C%11N(=O)=O)N(=O)=O | C64H60N2O4 | S | 4879,466 |
| 90 | 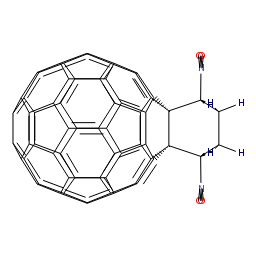 | c12c3c4[C@H]5[C@H]6[C@H]7[C@@H]3[C@H]3[C@@H]2[C@H]2c8c9c1c1[C@]%10%11c9c9c%12c8[C@@H]8[C@H]2[C@@H]2[C@H]3[C@H]3[C@H]%13[C@@H]2[C@H]2[C@@H]8[C@H]8[C@@H]%12c%12c9c9[C@@]%10(c%10c(c41)c5c1c4c%10c9c5c%12[C@H]9[C@H]%10[C@@H]5[C@@H]4[C@H]4[C@@H]1[C@@H]6[C@@H]([C@@H]73)[C@H]1[C@@H]%13[C@H]([C@@H]%10[C@@H]41)[C@@H]2[C@@H]89)[C@H](CC[C@H]%11N(=O)=O)N(=O)=O | C64H6N2O4 |  | 4703,679 |
| 91 | 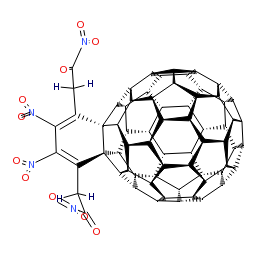 | c12c3c4[C@H]5[C@H]6[C@H]7[C@@H]3[C@H]3c2c2c8c9c1c1[C@]%10%11c9c9c%12c8[C@@H]8[C@H]2[C@@H]2[C@H]3[C@H]3[C@H]%13[C@@H]2[C@H]2[C@@H]8[C@H]8[C@@H]%12c%12c9c9[C@@]%10(c%10c(c41)c5c1c4c%10c9c5c%12[C@H]9[C@H]%10c5c4[C@H]4[C@@H]1[C@@H]6[C@@H]([C@@H]73)[C@H]1[C@@H]%13[C@H]([C@@H]%10[C@@H]41)[C@@H]2[C@@H]89)C(=C(C(=C%11CC=O)N(=O)=O)N(=O)=O)CC(=O)N(=O)=O.[N](=O)=O | C68H62N4O10 | S | 5437,01 |
| 92 | 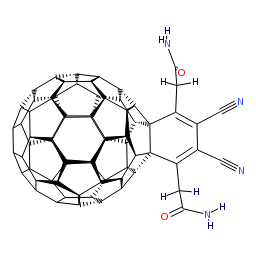 | c12c3c4[C@H]5[C@H]6[C@H]7[C@@H]3[C@H]3c2c2c8c9c1c1[C@]%10%11c9c9c%12c8[C@@H]8[C@H]2[C@@H]2[C@H]3[C@H]3[C@H]%13[C@@H]2[C@H]2[C@@H]8[C@H]8[C@@H]%12c%12c9c9[C@@]%10(c%10c(c41)c5c1c4c%10c9c5c%12[C@H]9[C@H]%10c5c4[C@H]4[C@@H]1[C@@H]6[C@@H]([C@@H]73)[C@H]1[C@@H]%13[C@H]([C@@H]%10[C@@H]41)[C@@H]2[C@@H]89)C(=C(C(=C%11CC(=O)N)C#N)C#N)CC(=O)N | C70H66N4O2 | S | 5328,009 |
| 93 | 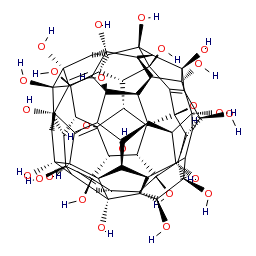 | C12=C3[C@]4([C@]5([C@]6(C7=C3[C@]3([C@@]2(C2=C8[C@@]9(C3=C3C%10=C%11C%12=C%13[C@]%14(C%15=C([C@@]8(C8=C%16[C@]%17(C%18=C%19[C@@]%20(C%21=C%17[C@]([C@@]%15%16O)(C%15=C%14C%14=C%16[C@@]%15(C%21=C%15[C@@]%20(C(=C4[C@@]%19(C1=C%18[C@]28O)O)C5=C1[C@]%15([C@]%16(C2=C([C@@]%12%14O)[C@]%11(C(=C6[C@]12O)[C@@]73O)O)O)O)O)O)O)O)O)O)[C@@]%13([C@]9%10O)O)O)O)O)O)O)O)O | C60H50O24 | S | 4735,289 |
| 94 | 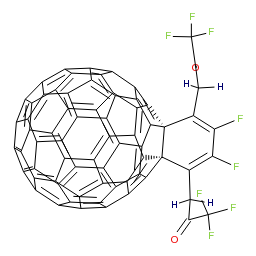 | c12c3c4[C@H]5[C@H]6[C@H]7[C@@H]3[C@H]3c2c2c8c9c1c1[C@]%10%11c9c9c%12c8[C@@H]8[C@H]2[C@@H]2[C@H]3[C@H]3[C@H]%13[C@@H]2[C@H]2[C@@H]8[C@H]8[C@@H]%12c%12c9c9[C@@]%10(c%10c(c41)c5c1c4c%10c9c5c%12[C@H]9[C@H]%10c5c4[C@H]4[C@@H]1[C@@H]6[C@@H]([C@@H]73)[C@H]1[C@@H]%13[C@H]([C@@H]%10[C@@H]41)[C@@H]2[C@@H]89)C(=C(C(=C%11CC(=O)C(F)(F)F)F)F)CC(=O)C(F)(F)F | C70H6O2F8 |  | 5572,082 |
| 95 | 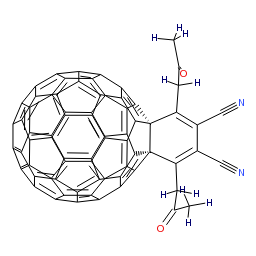 | c12c3c4[C@H]5[C@H]6[C@H]7[C@@H]3[C@H]3c2c2c8c9c1c1[C@]%10%11c9c9c%12c8[C@@H]8[C@H]2[C@@H]2[C@H]3[C@H]3[C@H]%13[C@@H]2[C@H]2[C@@H]8[C@H]8[C@@H]%12c%12c9c9[C@@]%10(c%10c(c41)c5c1c4c%10c9c5c%12[C@H]9[C@H]%10c5c4[C@H]4[C@@H]1[C@@H]6[C@@H]([C@@H]73)[C@H]1[C@@H]%13[C@H]([C@@H]%10[C@@H]41)[C@@H]2[C@@H]89)C(=C(C(=C%11CC(=O)C)C#N)C#N)CC(=O)C | C72H12N2O2 |  | 5554,704 |
| 96 | 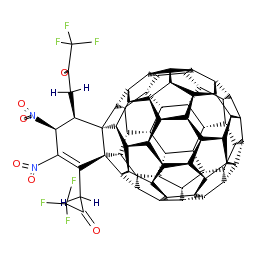 | c12c3c4[C@H]5[C@H]6[C@H]7[C@@H]3[C@H]3c2c2c8c9c1c1[C@]%10%11c9c9c%12c8[C@@H]8[C@H]2[C@@H]2[C@H]3[C@H]3[C@H]%13[C@@H]2[C@H]2[C@@H]8[C@H]8[C@@H]%12c%12c9c9[C@@]%10(c%10c(c41)c5c1c4c%10c9c5c%12[C@H]9[C@H]%10c5c4[C@H]4[C@@H]1[C@@H]6[C@@H]([C@@H]73)[C@H]1[C@@H]%13[C@H]([C@@H]%10[C@@H]41)[C@@H]2[C@@H]89)C(=C(C(=C%11CC(=O)C(F)(F)F)N(=O)=O)N(=O)=O)CC(=O)C(F)(F)F | C70H64N2O6F6 | S | 5718,981 |
| 97 | 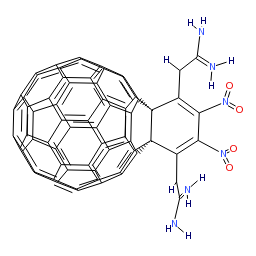 | c12c3c4[C@H]5[C@H]6[C@H]7[C@@H]3[C@H]3c2c2c8c9c1c1[C@]%10%11c9c9c%12c8[C@@H]8[C@H]2[C@@H]2[C@H]3[C@H]3[C@H]%13[C@@H]2[C@H]2[C@@H]8[C@H]8[C@@H]%12c%12c9c9[C@@]%10(c%10c(c41)c5c1c4c%10c9c5c%12[C@H]9[C@H]%10c5c4[C@H]4[C@@H]1[C@@H]6[C@@H]([C@@H]73)[C@H]1[C@@H]%13[C@H]([C@@H]%10[C@@H]41)[C@@H]2[C@@H]89)C(=C(C(=C%11C=C(N)N)[N](=O)O)N(=O)=O)C=C(N)N | C68H12N6O4 |  | 5478,684 |
| 98 | 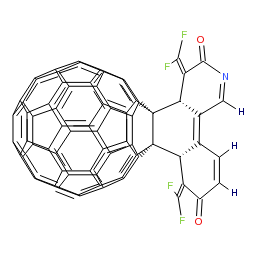 | c12c3c4[C@H]5[C@H]6[C@H]7[C@@H]3[C@H]3c2c2c8c9c1c1[C@]%10%11c9c9c%12c8[C@@H]8[C@H]2[C@@H]2[C@H]3[C@H]3[C@H]%13[C@@H]2[C@H]2[C@@H]8[C@H]8[C@@H]%12c%12c9c9[C@@]%10(c%10c(c41)c5c1c4c%10c9c5c%12[C@H]9[C@H]%10c5c4[C@H]4[C@@H]1[C@@H]6[C@@H]([C@@H]73)[C@H]1[C@@H]%13[C@H]([C@@H]%10[C@@H]41)[C@@H]2[C@@H]89)C1=C(C2=C%11C(=C(F)F)C(=O)N=C2)C=CC(=O)C1=C(F)F | C73H5NO2F4 |  | 5553,986 |
| 99 | 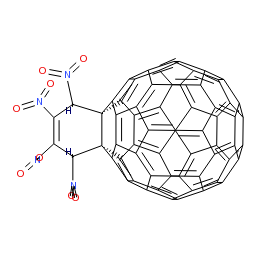 | c12c3c4[C@H]5[C@H]6[C@H]7[C@@H]3[C@H]3c2c2c8c9c1c1[C@]%10%11c9c9c%12c8[C@@H]8[C@H]2[C@@H]2[C@H]3[C@H]3[C@H]%13[C@@H]2[C@H]2[C@@H]8[C@H]8[C@@H]%12c%12c9c9[C@@]%10(c%10c(c41)c5c1c4c%10c9c5c%12[C@H]9[C@H]%10[C@@H]5[C@@H]4[C@H]4[C@@H]1[C@@H]6[C@@H]([C@@H]73)[C@H]1[C@@H]%13[C@H]([C@@H]%10[C@@H]41)[C@@H]2[C@@H]89)[C@H](C(=C([C@H]%11N(=O)=O)N(=O)=O)N(=O)=O)N(=O)=O | C64H2N4O8 |  | 5054,729 |
| 100 | 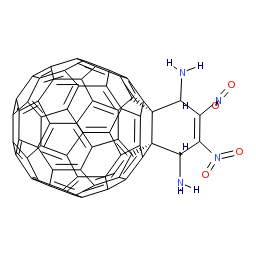 | c12c3c4[C@H]5[C@H]6[C@H]7[C@@H]3[C@H]3c2c2c8c9c1c1[C@]%10%11c9c9c%12c8[C@@H]8[C@H]2[C@@H]2[C@H]3[C@H]3[C@H]%13[C@@H]2[C@H]2[C@@H]8[C@H]8[C@@H]%12c%12c9c9[C@@]%10(c%10c(c41)c5c1c4c%10c9c5c%12[C@H]9[C@H]%10c5c4[C@H]4[C@@H]1[C@@H]6[C@@H]([C@@H]73)[C@H]1[C@@H]%13[C@H]([C@@H]%10[C@@H]41)[C@@H]2[C@@H]89)[C@H](C(=C([C@H]%11N)N(=O)=O)N(=O)=O)N | C64H6N4O4 |  | 4962,048 |
| 101 | 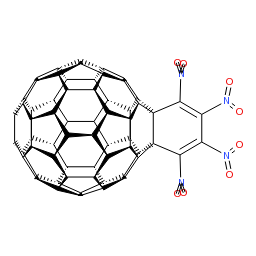 | c12c3c4[C@H]5[C@H]6[C@H]7[C@@H]3[C@H]3c2c2c8c9c1c1[C@]%10%11c9c9c%12c8[C@@H]8[C@H]2[C@@H]2[C@H]3[C@H]3[C@H]%13[C@@H]2[C@H]2[C@@H]8[C@H]8[C@@H]%12c%12c9c9[C@@]%10(c%10c(c41)c5c1c4c%10c9c5c%12[C@H]9[C@H]%10c5c4[C@H]4[C@@H]1[C@@H]6[C@@H]([C@@H]73)[C@H]1[C@@H]%13[C@H]([C@@H]%10[C@@H]41)[C@@H]2[C@@H]89)C(=C(C(=C%11N(=O)=O)N(=O)=O)N(=O)=O)N(=O)=O | C64H58N4O8 | S | 5202,75 |
| 102 | 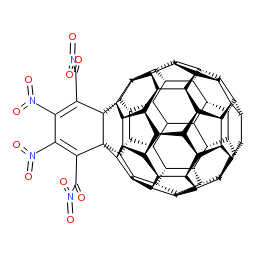 | c12c3c4[C@H]5[C@H]6[C@H]7[C@@H]3[C@H]3c2c2c8c9c1c1[C@]%10%11c9c9c%12c8[C@@H]8[C@H]2[C@@H]2[C@H]3[C@H]3[C@H]%13[C@@H]2[C@H]2[C@@H]8c8c%12c%12c9c9[C@@]%10(c%10c(c41)c5c1c4c%10c9c5c%12c9[C@H]%10c5c4[C@H]4[C@@H]1[C@@H]6[C@@H]([C@@H]73)[C@H]1[C@@H]%13C(=C2c89)[C@@H]%10[C@@H]41)C(=C(C(=C%11C=O)N(=O)=O)N(=O)=O)C(=O)N(=O)=O.[N](=O)=O | C66H58N4O10 | S | 5333,114 |
| 103 | 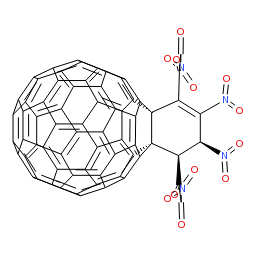 | c12c3c4[C@H]5[C@H]6[C@H]7[C@@H]3[C@H]3c2c2c8c9c1c1[C@]%10%11c9c9c%12c8[C@@H]8[C@H]2[C@@H]2[C@H]3[C@H]3[C@H]%13[C@@H]2[C@H]2[C@@H]8C8=C%12c%12c9c9[C@@]%10(c%10c(c41)c5c1c4c%10c9c5c%12[C@H]9[C@H]%10c5c4[C@H]4[C@@H]1[C@@H]6[C@@H]([C@@H]73)[C@H]1[C@@H]%13[C@H]([C@@H]%10[C@@H]41)[C@@H]2[C@@H]89)C(=C(C(=C%11C(=O)C(=O)N(=O)=O)N(=O)=O)N(=O)=O)C(=O)C(=O)N(=O)=O | C68H2N4O12 |  | 5367,563 |
| 104 | 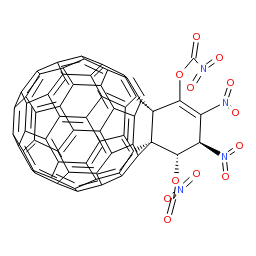 | c12c3c4[C@H]5[C@H]6[C@H]7[C@@H]3[C@H]3c2c2c8c9c1c1[C@]%10%11c9c9c%12c8[C@@H]8[C@H]2[C@@H]2[C@H]3[C@H]3[C@H]%13[C@@H]2[C@H]2[C@@H]8C8=C%12c%12c9c9[C@@]%10(c%10c(c41)c5c1c4c%10c9c5c%12[C@H]9[C@H]%10c5c4[C@H]4[C@@H]1[C@@H]6[C@@H]([C@@H]73)[C@H]1[C@@H]%13[C@H]([C@@H]%10[C@@H]41)[C@@H]2[C@@H]89)C(=C(C(=C%11OC(=O)N(=O)=O)N(=O)=O)N(=O)=O)OC(=O)N(=O)=O | C66H2N4O12 |  | 5387,092 |
| 105 | 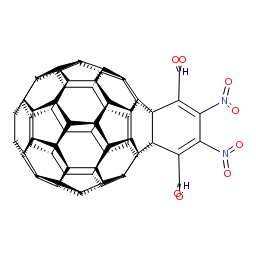 | c12c3c4[C@H]5[C@H]6[C@H]7[C@@H]3[C@H]3c2c2c8c9c1c1[C@]%10%11c9c9c%12c8[C@@H]8[C@H]2[C@@H]2[C@H]3[C@H]3[C@H]%13[C@@H]2[C@H]2[C@@H]8[C@H]8[C@@H]%12c%12c9c9[C@@]%10(c%10c(c41)c5c1c4c%10c9c5c%12[C@H]9[C@H]%10c5c4[C@H]4[C@@H]1[C@@H]6[C@@H]([C@@H]73)[C@H]1[C@@H]%13[C@H]([C@@H]%10[C@@H]41)[C@@H]2[C@@H]89)C(=C(C(=C%11C(=O)O)N(=O)=O)N(=O)=O)C(=O)O | C66H60N2O8 | S | 5200,893 |
| 106 | 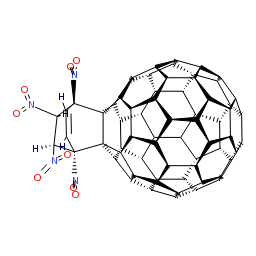 | c12c3c4[C@H]5[C@H]6[C@H]7[C@@H]3[C@H]3c2c2c8c9c1c1[C@]%10%11c9c9c%12c8[C@@H]8[C@H]2[C@@H]2[C@H]3[C@H]3[C@H]%13[C@@H]2[C@H]2[C@@H]8[C@H]8[C@@H]%12c%12c9c9[C@@]%10(c%10c(c41)c5c1c4c%10c9c5c%12[C@H]9[C@H]%10[C@@H]5[C@@H]4[C@H]4[C@@H]1[C@@H]6[C@@H]([C@@H]73)[C@H]1[C@@H]%13[C@H]([C@@H]%10[C@@H]41)[C@@H]2[C@@H]89)[C@@]1([C@H]([C@@H]([C@@]%11(N(=O)=O)C=C1)N(=O)=O)N(=O)=O)N(=O)=O | C66H62N4O8 | S | 5064,568 |
| 107 | 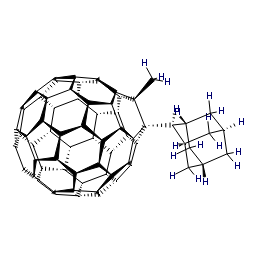 | c12c3c4[C@H]5[C@H]6[C@H]7[C@@H]3[C@H]3[C@@H]2[C@H]2c8c9c1c1[C@@]%10(c9c9c%11c8[C@@H]8[C@H]2[C@@H]2[C@H]3[C@H]3[C@H]%12[C@@H]2[C@H]2[C@@H]8[C@H]8[C@@H]%11c%11c9c9[C@@]%10(c%10c(c41)c5c1c4c%10c9c5c%11[C@H]9[C@H]%10c5c4[C@H]4[C@@H]1[C@@H]6[C@@H]([C@@H]73)[C@H]1[C@@H]%12[C@H]([C@@H]%10[C@@H]41)[C@@H]2[C@@H]89)[C@@H]1[C@@H]2C[C@@H]3C[C@H]1C[C@@H](C3)C2)C | C71H76 | S | 5241,619 |
| 108 | 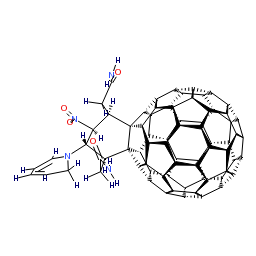 | c12c3c4[C@H]5[C@H]6[C@H]7[C@@H]3[C@H]3[C@@H]2[C@H]2c8c9c1c1[C@]%10%11c9c9c%12c8[C@@H]8[C@H]2[C@@H]2[C@H]3[C@H]3[C@H]%13[C@@H]2[C@H]2[C@@H]8[C@H]8[C@@H]%12c%12c9c9[C@@]%10(c%10c(c41)c5c1c4c%10c9c5c%12[C@H]9[C@H]%10c5c4[C@H]4[C@@H]1[C@@H]6[C@@H]([C@@H]73)[C@H]1[C@@H]%13[C@H]([C@@H]%10[C@@H]41)[C@@H]2[C@@H]89)[C@H]([C@@H]([C@H]([C@H]%11CC(=O)N)N(=O)=O)N1C=CC=CC1)CC(=O)N | C73H76N4O4 | S | 5704,02 |
| 109 | 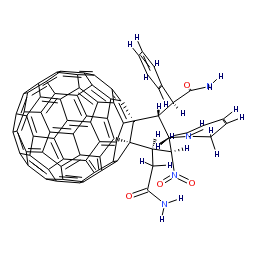 | c12c3c4[C@H]5[C@H]6[C@H]7[C@@H]3[C@H]3c2c2c8c9c1c1[C@]%10%11c9c9c%12c8[C@@H]8[C@H]2[C@@H]2[C@H]3[C@H]3[C@H]%13[C@@H]2[C@H]2[C@@H]8[C@H]8[C@@H]%12c%12c9c9[C@@]%10(c%10c(c41)c5c1c4c%10c9c5c%12[C@H]9[C@H]%10[C@@H]5[C@@H]4[C@H]4[C@@H]1[C@@H]6[C@@H]([C@@H]73)[C@H]1[C@@H]%13[C@H]([C@@H]%10[C@@H]41)[C@@H]2[C@@H]89)[C@@H]([C@@H]([C@H]([C@H]%11CC(=O)N)N(=O)=O)N1C=CC=CC1)[C@H](C(=O)N)c1ccccc1 | C79H22N4O4 |  | 5930,537 |
| 110 | 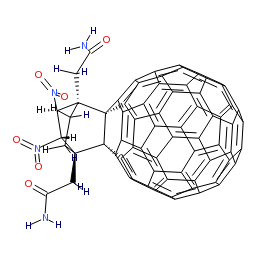 | c12c3c4[C@H]5[C@H]6[C@H]7[C@@H]3[C@H]3c2c2c8c9c1c1[C@]%10%11c9c9c%12c8[C@@H]8[C@H]2[C@@H]2[C@H]3[C@H]3[C@H]%13[C@@H]2[C@H]2[C@@H]8[C@H]8[C@@H]%12c%12c9c9[C@@]%10(c%10c(c41)c5c1c4c%10c9c5c%12[C@H]9[C@H]%10c5c4[C@H]4[C@@H]1[C@@H]6[C@@H]([C@@H]73)[C@H]1[C@@H]%13[C@H]([C@@H]%10[C@@H]41)[C@@H]2[C@@H]89)[C@]1([C@@H]([C@H]([C@]%11(CC(=O)N)CC1)N(=O)=O)N(=O)=O)CC(=O)N | C70H14N4O6 |  | 5439,441 |
| 111 | 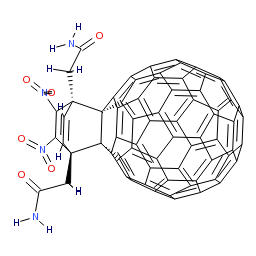 | c12c3c4[C@H]5[C@H]6[C@H]7[C@@H]3[C@H]3c2c2c8c9c1c1[C@]%10%11c9c9c%12c8[C@@H]8[C@H]2[C@@H]2[C@H]3[C@H]3[C@H]%13[C@@H]2[C@H]2[C@@H]8[C@H]8[C@@H]%12c%12c9c9[C@@]%10(c%10c(c41)c5c1c4c%10c9c5c%12[C@H]9[C@H]%10[C@@H]5[C@@H]4[C@H]4[C@@H]1[C@@H]6[C@@H]([C@@H]73)[C@H]1[C@@H]%13[C@H]([C@@H]%10[C@@H]41)[C@@H]2[C@@H]89)[C@]1(C(=C([C@]%11(CC(=O)N)C=C1)N(=O)=O)N(=O)=O)CC(=O)N | C70H10N4O6 |  | 5364,992 |
| 112 |  | c1(nc(nc(n1)N)N)N/N=C/c1ccc([C@H]2[C@@]34c5c6c7c8c9c%10[C@H]%11[C@H]%12[C@H]%13[C@@H]9[C@H]9c8c8c6c6c%14c5c5c%15[C@@]3(c3c(c%10c47)c%11c4c7c3c%15c3c%10c5[C@H]%14[C@@H]5[C@@H]%11[C@H]6[C@H]8[C@@H]6[C@H]9[C@H]8[C@H]9[C@@H]6[C@@H]%11[C@H]6[C@@H]5[C@@H]%10[C@H]5[C@@H]3[C@@H]7[C@H]3[C@@H]4[C@@H]%12[C@@H]([C@@H]%138)[C@H]4[C@@H]9[C@@H]6[C@@H]5[C@@H]34)CN2C)cc1 | C73H76N8 | S | 6311,092 |
| 113 |  | c1(nc(nc(n1)NC)N)N/N=C/c1ccc([C@H]2[C@@]34c5c6c7c8c9c%10[C@H]%11[C@H]%12[C@H]%13[C@@H]9[C@H]9c8c8c6c6c%14c5c5c%15[C@@]3(c3c(c%10c47)c%11c4c7c3c%15c3c%10c5[C@H]%14[C@@H]5[C@@H]%11[C@H]6[C@H]8[C@@H]6[C@H]9[C@H]8[C@H]9[C@@H]6[C@@H]%11[C@H]6[C@@H]5[C@@H]%10[C@H]5[C@@H]3[C@@H]7[C@H]3[C@@H]4[C@@H]%12[C@@H]([C@@H]%138)[C@H]4[C@@H]9[C@@H]6[C@@H]5[C@@H]34)CN2C)cc1 | C74H78N8 | S | 6414,5 |
| 114 | 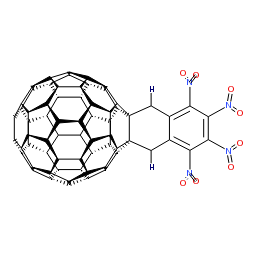 | c12c3c4c5c6c7c3[C@H]3c2c2c8c9c1c1[C@]%10%11c9c9c%12c8[C@@H]8[C@H]2[C@@H]2[C@H]3C3=C7[C@H]7[C@H]%13[C@@H]%14C%15=C%16c%17c(c%12c%12c9c9[C@@]%10(c%10c(c41)c5c1c4c%10c9c5c%12c%17[C@H]9c5c4[C@@H]([C@@H]1[C@@H]67)[C@@H]%13[C@@H]%159)C=c1c(=C%11)c(c(c(c1N(=O)=O)N(=O)=O)N(=O)=O)N(=O)=O)[C@H]8[C@@H]%16[C@H]2[C@H]3%14 | C68H62N4O8 | S | 5753,991 |
| 115 | 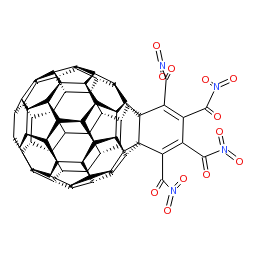 | c12c3c4[C@H]5[C@H]6[C@H]7[C@@H]3[C@H]3c2c2c8c9c1c1[C@]%10%11c9c9c%12c8[C@@H]8[C@H]2[C@@H]2[C@H]3[C@H]3[C@H]%13[C@@H]2[C@H]2[C@@H]8[C@H]8[C@@H]%12c%12c9c9[C@@]%10(c%10c(c41)c5c1c4c%10c9c5c%12[C@H]9[C@H]%10c5c4[C@H]4[C@@H]1[C@@H]6[C@@H]([C@@H]73)[C@H]1[C@@H]%13[C@H]([C@@H]%10[C@@H]41)[C@@H]2[C@@H]89)C(=C(C(=C%11C(=O)N(=O)=O)C(=O)N(=O)=O)C(=O)N(=O)=O)C=O.[N](=O)=O | C68H58N4O12 | S | 5580,421 |
| 116 | 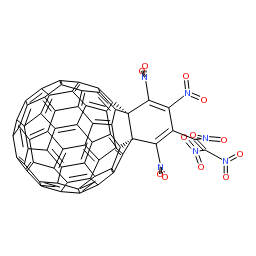 | c12c3c4[C@H]5[C@H]6[C@H]7[C@@H]3[C@H]3c2c2c8c9c1c1[C@]%10%11c9c9c%12c8[C@@H]8[C@H]2[C@@H]2[C@H]3[C@H]3[C@H]%13[C@@H]2[C@H]2[C@@H]8[C@H]8[C@@H]%12c%12c9c9[C@@]%10(c%10c(c41)c5c1c4c%10c9c5c%12[C@H]9[C@H]%10c5c4[C@H]4[C@@H]1[C@@H]6[C@@H]([C@@H]73)[C@H]1[C@@H]%13[C@H]([C@@H]%10[C@@H]41)[C@@H]2[C@@H]89)C(=C(C(=C%11N(=O)=O)N(=O)=O)C(=C(N(=O)=O)N(=O)=O)N(=O)=O)N(=O)=O | C66N6O12 |  | 5564,405 |
| 117 | 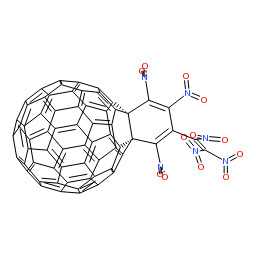 | c12c3c4[C@H]5[C@H]6[C@H]7[C@@H]3[C@H]3c2c2c8c9c1c1[C@]%10%11c9c9c%12c8[C@@H]8[C@H]2[C@@H]2[C@H]3[C@H]3[C@H]%13[C@@H]2[C@H]2[C@@H]8[C@H]8[C@@H]%12c%12c9c9[C@@]%10(c%10c(c41)c5c1c4c%10c9c5c%12[C@H]9[C@H]%10c5c4[C@H]4[C@@H]1[C@@H]6[C@@H]([C@@H]73)[C@H]1[C@@H]%13[C@H]([C@@H]%10[C@@H]41)[C@@H]2[C@@H]89)C(=C(C(=C%11N(=O)=O)C(=C(N(=O)=O)N(=O)=O)N(=O)=O)N(=O)=O)N(=O)=O | C66N6O12 |  | 5546,46 |
| 118 | 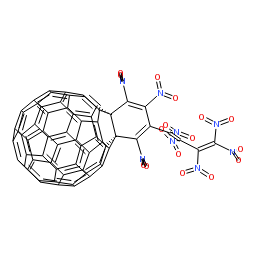 | c12c3c4[C@H]5[C@H]6[C@H]7[C@@H]3[C@H]3[C@@H]2[C@H]2c8c9c1c1[C@]%10%11c9c9c%12c8[C@@H]8[C@H]2[C@@H]2[C@H]3[C@H]3[C@H]%13[C@@H]2[C@H]2[C@@H]8[C@H]8[C@@H]%12c%12c9c9[C@@]%10(c%10c(c41)c5c1c4c%10c9c5c%12[C@H]9[C@H]%10c5c4[C@H]4[C@@H]1[C@@H]6[C@@H]([C@@H]73)[C@H]1[C@@H]%13[C@H]([C@@H]%10[C@@H]41)[C@@H]2[C@@H]89)C(=C(C(=C%11N(=O)=O)/C(=C(/C(=C(N(=O)=O)N(=O)=O)N(=O)=O)\N(=O)=O)/N(=O)=O)N(=O)=O)N(=O)=O | C68N8O16 |  | 6008,435 |
| 119 | 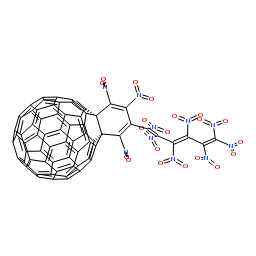 | c12c3c4C5=C6[C@H]7[C@@H]3[C@H]3c2c2c8c9c1c1[C@]%10%11c9c9c%12c8[C@@H]8[C@H]2[C@@H]2[C@H]3[C@H]3[C@H]%13[C@@H]2[C@H]2[C@@H]8[C@H]8[C@@H]%12c%12c9c9[C@@]%10(c%10c(c41)c5c1c4c%10c9c5c%12[C@H]9[C@H]%10c5c4[C@H]4[C@@H]1[C@@H]6[C@@H]([C@@H]73)[C@H]1[C@@H]%13[C@H]([C@@H]%10[C@@H]41)[C@@H]2[C@@H]89)C(=C(C(=C%11N(=O)=O)/C(=C(/C(=C(\N(=O)=O)/C(=C(N(=O)=O)N(=O)=O)N(=O)=O)/N(=O)=O)\N(=O)=O)/N(=O)=O)N(=O)=O)N(=O)=O | C70H2N10O20 |  | 6492,231 |
| 120 | 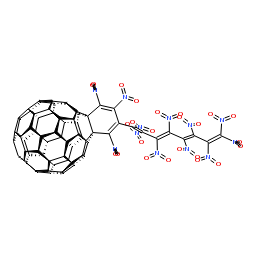 | c12c3c4[C@H]5[C@H]6[C@H]7[C@@H]3[C@H]3[C@@H]2[C@H]2c8c9c1c1[C@]%10%11c9c9c%12c8[C@@H]8[C@H]2[C@@H]2[C@H]3[C@H]3[C@H]%13[C@@H]2[C@H]2[C@@H]8[C@H]8[C@@H]%12c%12c9c9[C@@]%10(c%10c(c41)c5c1c4c%10c9c5c%12[C@H]9[C@H]%10c5c4[C@H]4[C@@H]1[C@@H]6[C@@H]([C@@H]73)[C@H]1[C@@H]%13[C@H]([C@@H]%10[C@@H]41)[C@@H]2[C@@H]89)C(=C(C(=C%11N(=O)=O)/C(=C(/C(=C(\N(=O)=O)/C(=C(/C(=C(N(=O)=O)N(=O)=O)N(=O)=O)\N(=O)=O)/N(=O)=O)/N(=O)=O)\N(=O)=O)/N(=O)=O)N(=O)=O)N(=O)=O | C72H58N12O24 | S | 6828,544 |
| 121 | 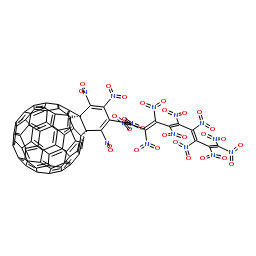 | c12c3c4[C@H]5[C@H]6C7=C3[C@H]3c2c2c8c9c1c1[C@]%10%11c9c9c%12c8[C@@H]8[C@H]2[C@@H]2[C@H]3C3=C7[C@H]7[C@H]%13[C@@H]%14[C@@H]%15[C@H]%16[C@@H]%17c%18c%19c%20c([C@@]%10(c%10c(c41)c5c1c(c%20%10)c%18[C@@H]([C@@H]1[C@@H]67)[C@@H]%13%16)C(=C(C(=C%11N(=O)=O)/C(=C(/C(=C(/C(=C(/C(=C(/C(=C(N(=O)=O)N(=O)=O)N(=O)=O)\N(=O)=O)/N(=O)=O)\N(=O)=O)/N(=O)=O)\N(=O)=O)/N(=O)=O)\N(=O)=O)/N(=O)=O)N(=O)=O)N(=O)=O)c9c1[C@H]%12[C@@H]4[C@H]8[C@@H]([C@H]%15[C@@H]4[C@H]%17c%191)[C@H]2[C@H]3%14 | C74N14O28 |  | 7120,258 |
| 122 | 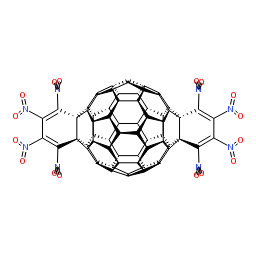 | c12c3c4[C@H]5[C@H]6c7c3c3c2c2c8c9c1c1[C@]%10%11c9c9c%12c8c8c2c2c3c3[C@@]%13%14c2c2c8[C@H]8[C@@H]%12c%12c9c9[C@@]%10(c%10c(c41)c5c1c4c%10c9c5c%12c9c%10c5c4c4c1c6c(c73)c1[C@@]%13(c(c%10c41)c2c89)C(=C(C(=C%14N(=O)=O)N(=O)=O)N(=O)=O)N(=O)=O)C(=C(C(=C%11N(=O)=O)N(=O)=O)N(=O)=O)N(=O)=O | C68H56N8O16 | S | 5885,513 |
| 123 | 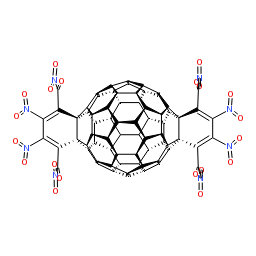 | c12c3c4c5c6c7c3c3c2c2c8c9c1c1[C@]%10%11c9c9c%12c8c8c2c2c3c3[C@@]%13%14c2c2c8c8c%12c%12c9c9[C@@]%10(c%10c(c41)c5c1c4c%10c9c5c%12c9c%10c5c4c4c1c6c(c73)c1[C@@]%13(c(c%10c41)c2c89)C(=C(C(=C%14C(=O)N(=O)=O)N(=O)=O)N(=O)=O)C(=O)N(=O)=O)C(=C(C(=C%11C(=O)N(=O)=O)N(=O)=O)N(=O)=O)C=O.[N](=O)=O | C72H56N8O20 | S | 6171,418 |
| 124 | 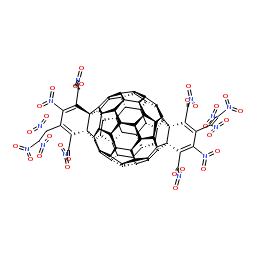 | c12c3c4[C@H]5[C@H]6c7c3c3c2c2c8c9c1c1[C@]%10%11c9c9c%12c8c8c2c2c3c3[C@@]%13%14c2c2c8c8c%12c%12c9c9[C@@]%10(c%10c(c41)c5c1c4c%10c9c5c%12c9c%10c5c4c4c1c6c(c73)c1[C@@]%13(c(c%10c41)c2c89)C(=C(C(=C%14C(=O)N(=O)=O)N(=O)=O)C(=C(N(=O)=O)N(=O)=O)N(=O)=O)C(=O)N(=O)=O)C(=C(C(=C%11C(=O)N(=O)=O)C(=C(N(=O)=O)N(=O)=O)N(=O)=O)N(=O)=O)C(=O)N(=O)=O | C76H58N12O28 | S | 6650,551 |
| 125 | 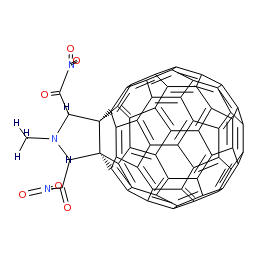 | c12c3c4[C@H]5[C@H]6[C@H]7[C@@H]3[C@H]3c2c2c8c9c1c1[C@]%10%11c9c9c%12c8[C@@H]8[C@H]2[C@@H]2[C@H]3[C@H]3[C@H]%13[C@@H]2[C@H]2[C@@H]8[C@H]8[C@@H]%12c%12c9c9[C@@]%10(c%10c(c41)c5c1c4c%10c9c5c%12[C@H]9[C@H]%10c5c4[C@H]4[C@@H]1[C@@H]6[C@@H]([C@@H]73)[C@H]1[C@@H]%13[C@H]([C@@H]%10[C@@H]41)[C@@H]2[C@@H]89)[C@H](N([C@H]%11C(=O)N(=O)=O)C)C(=O)N(=O)=O | C65H5N3O6 |  | 5075,167 |
| 126 | 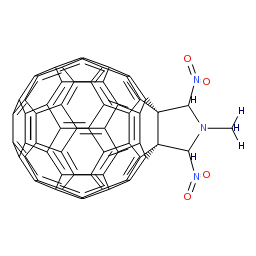 | c12c3c4[C@H]5[C@H]6[C@H]7[C@@H]3[C@H]3c2c2c8c9c1c1[C@]%10%11c9c9c%12c8[C@@H]8[C@H]2[C@@H]2[C@H]3[C@H]3[C@H]%13[C@@H]2[C@H]2[C@@H]8[C@H]8[C@@H]%12c%12c9c9[C@@]%10(c%10c(c41)c5c1c4c%10c9c5c%12[C@H]9[C@H]%10c5c4[C@H]4[C@@H]1[C@@H]6[C@@H]([C@@H]73)[C@H]1[C@@H]%13[C@H]([C@@H]%10[C@@H]41)[C@@H]2[C@@H]89)[C@H](N([C@H]%11N(=O)=O)C)N(=O)=O | C63H5N3O4 |  | 4782,199 |
| 127 | 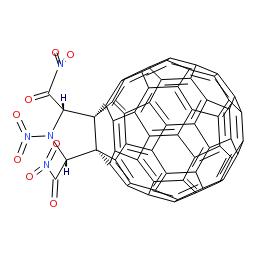 | c12c3c4[C@H]5[C@H]6[C@H]7[C@@H]3[C@H]3c2c2c8c9c1c1[C@]%10%11c9c9c%12c8[C@@H]8[C@H]2[C@@H]2[C@H]3[C@H]3[C@H]%13[C@@H]2[C@H]2[C@@H]8C8=C%12c%12c9c9[C@@]%10(c%10c(c41)c5c1c4c%10c9c5c%12[C@H]9[C@H]%10c5c4[C@H]4[C@@H]1[C@@H]6[C@@H]([C@@H]73)[C@H]1[C@@H]%13[C@H]([C@@H]%10[C@@H]41)[C@@H]2[C@@H]89)[C@H](N([C@H]%11C(=O)N(=O)=O)N(=O)=O)C(=O)N(=O)=O | C64H2N4O8 |  | 5079,182 |
| 128 |  | c12c3c4[C@H]5[C@H]6[C@H]7[C@@H]3[C@H]3[C@@H]2[C@H]2c8c9c1c1[C@]%10%11c9c9c%12c8[C@@H]8[C@H]2[C@@H]2[C@H]3[C@H]3[C@H]%13[C@@H]2[C@H]2[C@@H]8[C@H]8[C@@H]%12c%12c9c9[C@@]%10(c%10c(c41)c5c1c4c%10c9c5c%12[C@H]9[C@H]%10[C@@H]5[C@@H]4[C@H]4[C@@H]1[C@@H]6[C@@H]([C@@H]73)[C@H]1[C@@H]%13[C@H]([C@@H]%10[C@@H]41)[C@@H]2[C@@H]89)C(=O)N(C%11=O)C | C63H61NO2 | S | 4807,826 |
| 129 |  | c12c3c4[C@H]5[C@H]6[C@H]7[C@@H]3[C@H]3c2c2c8c9c1c1[C@]%10%11c9c9c%12c8[C@@H]8[C@H]2[C@@H]2[C@H]3[C@H]3[C@H]%13[C@@H]2[C@H]2[C@@H]8C8=C%12c%12c9c9[C@@]%10(c%10c(c41)c5c1c4c%10c9c5c%12[C@H]9[C@H]%10c5c4[C@H]4[C@@H]1[C@@H]6[C@@H]([C@@H]73)[C@H]1[C@@H]%13[C@H]([C@@H]%10[C@@H]41)[C@@H]2[C@@H]89)[C@H](N([C@H]%11C(=O)N(=O)=O)C(=O)N(=O)=O)C(=O)N(=O)=O | C65H2N4O9 |  | 5221,905 |
| 130 |  | c12c3c4[C@H]5[C@H]6[C@H]7[C@@H]3[C@H]3c2c2c8c9c1c1[C@]%10%11c9c9c%12c8[C@@H]8[C@H]2[C@@H]2[C@H]3[C@H]3[C@H]%13[C@@H]2[C@H]2[C@@H]8[C@H]8[C@@H]%12c%12c9c9[C@@]%10(c%10c(c41)c5c1c4c%10c9c5c%12[C@H]9[C@H]%10c5c4[C@H]4[C@@H]1[C@@H]6[C@@H]([C@@H]73)[C@H]1[C@@H]%13[C@H]([C@@H]%10[C@@H]41)[C@@H]2[C@@H]89)C(=C(C(=C%11N(=O)=O)c1ccccc1)N(=O)=O)N(=O)=O | C70H63N3O6 | S | 5744,514 |
| 131 |  | c12c3c4[C@H]5[C@H]6[C@H]7[C@@H]3[C@H]3c2c2c8c9c1c1[C@]%10%11c9c9c%12c8[C@@H]8[C@H]2[C@@H]2[C@H]3[C@H]3[C@H]%13[C@@H]2[C@H]2[C@@H]8[C@H]8[C@@H]%12c%12c9c9[C@@]%10(c%10c(c41)c5c1c4c%10c9c5c%12[C@H]9[C@H]%10c5c4[C@H]4C1=C6C(=C1[C@@H]%13[C@H]([C@@H]%10[C@@H]41)[C@@H]2[C@@H]89)[C@@H]73)C(=C(C(=C%11N(=O)=O)CC1CCCCC1)N(=O)=O)N(=O)=O | C71H15N3O6 |  | 5924,623 |
| 132 |  | c12c3c4[C@H]5[C@H]6[C@H]7[C@@H]3[C@H]3c2c2c8c9c1c1[C@]%10%11c9c9c%12c8[C@@H]8[C@H]2[C@@H]2[C@H]3[C@H]3[C@H]%13[C@@H]2[C@H]2[C@@H]8[C@H]8[C@@H]%12c%12c9c9[C@@]%10(c%10c(c41)c5c1c4c%10c9c5c%12[C@H]9[C@H]%10c5c4[C@H]4[C@@H]1[C@@H]6[C@@H]([C@@H]73)[C@H]1[C@@H]%13[C@H]([C@@H]%10[C@@H]41)[C@@H]2[C@@H]89)C(=C(C(=C%11C)c1ccccc1)N(=O)=O)C | C72H69NO2 | S | 5673,948 |
| 133 |  | c12c3c4[C@H]5[C@H]6[C@H]7[C@@H]3[C@H]3c2c2c8c9c1c1[C@]%10%11c9c9c%12c8[C@@H]8[C@H]2[C@@H]2[C@H]3[C@H]3[C@H]%13[C@@H]2[C@H]2[C@@H]8[C@H]8[C@@H]%12c%12c9c9[C@@]%10(c%10c(c41)c5c1c4c%10c9c5c%12C9=C8C2=C2[C@H]8[C@@H]9c5c4[C@H]4[C@@H]1[C@@H]6[C@@H]([C@@H]73)[C@@H]([C@@H]%132)[C@@H]84)C(=C(C(=C%11F)c1ccccc1)F)F | C70H63F3 | S | 5473,031 |
| 134 |  | c12c3c4[C@H]5[C@H]6[C@H]7[C@@H]3[C@H]3c2c2c8c9c1c1[C@]%10%11c9c9c%12c8[C@@H]8[C@H]2[C@@H]2[C@H]3[C@H]3[C@H]%13[C@@H]2[C@H]2[C@@H]8[C@H]8[C@@H]%12c%12c9c9[C@@]%10(c%10c(c41)c5c1c4c%10c9c5c%12[C@H]9[C@H]%10c5c4[C@H]4[C@@H]1[C@@H]6[C@@H]([C@@H]73)[C@H]1[C@@H]%13[C@H]([C@@H]%10[C@@H]41)[C@@H]2[C@@H]89)C(=C(C(=C%11C(=O)O)c1ccccc1)N(=O)=O)C(=O)O | C72H65NO6 | S | 5681,984 |
| 135 |  | c12c3c4[C@H]5[C@H]6[C@H]7[C@@H]3[C@H]3c2c2c8c9c1c1[C@]%10%11c9c9c%12c8[C@@H]8[C@H]2[C@@H]2[C@H]3[C@H]3[C@H]%13[C@@H]2[C@H]2[C@@H]8C8=C%12c%12c9c9[C@@]%10(c%10c(c41)c5c1c4c%10c9c5c%12[C@H]9[C@H]%10c5c4[C@H]4[C@@H]1[C@@H]6[C@@H]([C@@H]73)[C@H]1[C@@H]%13[C@H]([C@@H]%10[C@@H]41)[C@@H]2[C@@H]89)C(=C(C(=C%11C=O)c1ccccc1)N(=O)=O)C(=O)N(=O)=O.[N](=O)=O | C72H5N3O8 |  | 5732,695 |
| 136 |  | c12c3c4[C@H]5[C@H]6[C@H]7[C@@H]3[C@H]3c2c2c8c9c1c1[C@]%10%11c9c9c%12c8[C@@H]8[C@H]2[C@@H]2[C@H]3[C@H]3[C@H]%13[C@@H]2[C@H]2[C@@H]8[C@H]8[C@@H]%12c%12c9c9[C@@]%10(c%10c(c41)c5c1c4c%10c9c5c%12[C@H]9[C@H]%10c5c4[C@H]4[C@@H]1[C@@H]6[C@@H]([C@@H]73)[C@H]1[C@@H]%13[C@H]([C@@H]%10[C@@H]41)[C@@H]2[C@@H]89)C(=C(C(=C%11C(=O)C)c1ccccc1)N(=O)=O)C(=O)C | C74H11NO4 |  | 5809,037 |
| 137 |  | c12c3c4[C@H]5[C@H]6[C@H]7[C@@H]3[C@H]3c2c2c8c9c1c1[C@]%10%11c9c9c%12c8[C@@H]8[C@H]2[C@@H]2[C@H]3[C@H]3[C@H]%13[C@@H]2[C@H]2[C@@H]8[C@H]8[C@@H]%12c%12c9c9[C@@]%10(c%10c(c41)c5c1c4c%10c9c5c%12[C@H]9[C@H]%10c5c4[C@H]4C1=C6C(=C1[C@@H]%13[C@H]([C@@H]%10[C@@H]41)[C@@H]2[C@@H]89)[C@@H]73)C(=C(C(=C%11N(=O)=O)c1ccccc1)[N](=O)O)N | C70H67N3O4 | S | 5657,758 |
| 138 |  | c12c3c4[C@H]5[C@H]6[C@H]7[C@@H]3[C@H]3c2c2c8c9c1c1[C@]%10%11c9c9c%12c8[C@@H]8[C@H]2[C@@H]2[C@H]3[C@H]3[C@H]%13[C@@H]2[C@H]2[C@@H]8[C@H]8[C@@H]%12c%12c9c9[C@@]%10(c%10c(c41)c5c1c4c%10c9c5c%12[C@H]9[C@H]%10c5c4[C@H]4[C@@H]1[C@@H]6[C@@H]([C@@H]73)[C@H]1[C@@H]%13[C@H]([C@@H]%10[C@@H]41)[C@@H]2[C@@H]89)C(=C(C(=C%11N)c1ccccc1)N(=O)=O)N(=O)=O | C70H65N3O4 | S | 5584,371 |
| 139 |  | c12c3c4[C@H]5[C@H]6[C@H]7[C@@H]3[C@H]3c2c2c8c9c1c1[C@]%10%11c9c9c%12c8[C@@H]8[C@H]2[C@@H]2[C@H]3[C@H]3[C@H]%13[C@@H]2[C@H]2[C@@H]8[C@H]8[C@@H]%12c%12c9c9[C@@]%10(c%10c(c41)c5c1c4c%10c9c5c%12[C@H]9[C@H]%10c5c4[C@H]4[C@@H]1[C@@H]6[C@@H]([C@@H]73)[C@H]1[C@@H]%13[C@H]([C@@H]%10[C@@H]41)[C@@H]2[C@@H]89)C(=C(C(=C%11N(C)C)c1ccccc1)N(=O)=O)N(=O)=O | C72H69N3O4 | S | 5780,394 |
| 140 |  | c12c3c4[C@H]5[C@H]6[C@H]7[C@@H]3[C@H]3c2c2c8c9c1c1[C@]%10%11c9c9c%12c8[C@@H]8[C@H]2[C@@H]2[C@H]3[C@H]3[C@H]%13[C@@H]2[C@H]2[C@@H]8[C@H]8[C@@H]%12c%12c9c9[C@@]%10(c%10c(c41)c5c1c4c%10c9c5c%12[C@H]9[C@H]%10c5c4[C@H]4[C@@H]1[C@@H]6[C@@H]([C@@H]73)[C@H]1[C@@H]%13[C@H]([C@@H]%10[C@@H]41)[C@@H]2[C@@H]89)C(=C(C(=C%11N(=O)=O)[C@H]1[C@@H]2C[C@@H]3C[C@H]1C[C@H](C2)C3)N(=O)=O)N(=O)=O | C74H73N3O6 | S | 5958,677 |
| 141 |  | c12c3c4[C@H]5[C@H]6[C@H]7[C@@H]3[C@H]3c2c2c8c9c1c1[C@]%10%11c9c9c%12c8[C@@H]8[C@H]2[C@@H]2[C@H]3[C@H]3[C@H]%13[C@@H]2[C@H]2[C@@H]8[C@H]8[C@@H]%12c%12c9c9[C@@]%10(c%10c(c41)c5c1c4c%10c9c5c%12[C@H]9[C@H]%10c5c4[C@H]4[C@@H]1[C@@H]6[C@@H]([C@@H]73)[C@H]1[C@@H]%13[C@H]([C@@H]%10[C@@H]41)[C@@H]2[C@@H]89)C(=C(C(=C%11N(=O)=O)C1=CC=CC=CC=C1)N(=O)=O)N(=O)=O | C72H65N3O6 | S | 5905,191 |
| 142 |  | c12c3c4[C@H]5[C@H]6[C@H]7[C@@H]3[C@H]3c2c2c8c9c1c1[C@]%10%11c9c9c%12c8[C@@H]8[C@H]2[C@@H]2[C@H]3[C@H]3[C@H]%13[C@@H]2[C@H]2[C@@H]8[C@H]8[C@@H]%12c%12c9c9[C@@]%10(c%10c(c41)c5c1c4c%10c9c5c%12[C@H]9[C@H]%10c5c4[C@H]4[C@@H]1[C@@H]6[C@@H]([C@@H]73)[C@H]1[C@@H]%13[C@H]([C@@H]%10[C@@H]41)[C@@H]2[C@@H]89)C(=C(C(=C%11N(=O)=O)c1cc(cc(c1)C(F)(F)F)C(F)(F)F)N(=O)=O)N(=O)=O | C72H3N3O6F6 |  | 6154,838 |
| 143 |  | c12c3c4[C@H]5[C@H]6[C@H]7[C@@H]3[C@H]3c2c2c8c9c1c1[C@]%10%11c9c9c%12c8C8=C%13[C@H]%14[C@@H]%12c%12c9c9[C@@]%10(c%10c(c41)c5c1c4c%10c9c5c%12[C@H]9[C@H]%10c5c4[C@H]4[C@@H]1[C@@H]6[C@H]1[C@@H]7[C@H]5[C@@H]3[C@@H]([C@@H]28)C2=C%13[C@@H]([C@@H]%149)[C@H]3[C@@H]%10[C@@H]4[C@@H]1[C@H]3[C@@H]52)C(=C(C(=C%11N(=O)=O)c1cc(cc(c1)C)C)N(=O)=O)N(=O)=O | C72H67N3O6 | S | 5937,964 |
| 144 |  | c12c3c4[C@H]5[C@H]6[C@H]7[C@@H]3[C@H]3[C@@H]2[C@H]2c8c9c1c1[C@]%10%11c9c9c%12c8[C@@H]8[C@H]2[C@@H]2[C@H]3[C@H]3[C@H]%13[C@@H]2[C@H]2[C@@H]8[C@H]8[C@@H]%12c%12c9c9[C@@]%10(c%10c(c41)c5c1c4c%10c9c5c%12[C@H]9[C@H]%10c5c4[C@H]4[C@@H]1[C@@H]6[C@@H]([C@@H]73)[C@H]1[C@@H]%13[C@H]([C@@H]%10[C@@H]41)[C@@H]2[C@@H]89)C(=C(C(=C%11N)c1ccccc1)[N](=O)O)N | C70H69N3O2 | S | 5536,756 |
| 145 |  | c12c3c4[C@H]5[C@H]6[C@H]7[C@@H]3[C@H]3[C@@H]2[C@H]2c8c9c1c1[C@]%10%11c9c9c%12c8[C@@H]8[C@H]2[C@@H]2[C@H]3[C@H]3[C@H]%13[C@@H]2[C@H]2[C@@H]8[C@H]8[C@@H]%12[C@H]%12c9c9[C@@]%10(c%10c(c41)c5c1c4c%10c9c5[C@H]%12[C@H]9[C@H]%10c5c4[C@H]4[C@@H]1[C@@H]6[C@@H]([C@@H]73)[C@H]1[C@@H]%13[C@H]([C@@H]%10[C@@H]41)[C@@H]2[C@@H]89)C(=C(C(=C%11N(=O)=O)C1CCCCC1)N)[N](=O)O | C70H65N3O4 | S | 5660,19 |
| 146 |  | c12c3c4[C@H]5[C@H]6c7c3c3c2c2c8c9c1c1[C@]%10%11c9c9c%12c8c8c2c2c3c3[C@@]%13%14c2c2c8[C@H]8[C@@H]%12c%12c9c9[C@@]%10(c%10c(c41)c5c1c4c%10c9c5c%12c9c%10c5c4c4c1c6c(c73)c1[C@@]%13(c(c%10c41)c2c89)C(=C[C@H](N%14C(=O)O)C(=O)N[C@@H](C)C(=O)N)C(=O)O)N([C@H](C=C%11C(=O)O)C(=O)N[C@H](C(=O)N)C)C(=O)O | C78H80N6O12 | S | 6555,683 |
| 147 |  | c12c3c4[C@H]5[C@H]6[C@H]7[C@@H]3[C@H]3c2c2c8c9c1c1[C@]%10%11c9c9c%12c8[C@@H]8[C@H]2[C@@H]2[C@H]3[C@H]3[C@H]%13[C@@H]2[C@H]2[C@@H]8[C@H]8[C@@H]%12c%12c9c9[C@@]%10(c%10c(c41)c5c1c4c%10c9c5c%12[C@H]9[C@H]%10c5c4[C@H]4[C@@H]1[C@@H]6[C@@H]([C@@H]73)[C@H]1[C@@H]%13[C@H]([C@@H]%10[C@@H]41)[C@@H]2[C@@H]89)C%11(C(=O)OC(=O)O)C(=O)OC(=O)O | C65H60O8 | S | 4954,095 |
| 148 |  | c12c3c4[C@H]5[C@H]6[C@H]7[C@@H]3[C@H]3c2c2c8c9c1c1[C@]%10%11c9c9c%12c8[C@@H]8[C@H]2[C@@H]2[C@H]3[C@H]3[C@H]%13[C@@H]2[C@H]2[C@@H]8C8=C%12c%12c9c9[C@@]%10(c%10c(c41)c5c1c4c%10c9c5c%12[C@H]9[C@H]%10c5c4[C@H]4[C@@H]1[C@@H]6[C@@H]([C@@H]73)[C@H]1[C@@H]%13[C@H]([C@@H]%10[C@@H]41)[C@@H]2[C@@H]89)C%11(C(=O)OCN)C(=O)OCN | C65H66N2O4 | S | 5113,078 |
| 149 |  | c12c3c4[C@H]5[C@H]6[C@H]7[C@@H]3[C@H]3c2c2c8c9c1c1[C@]%10%11c9c9c%12c8[C@@H]8[C@H]2[C@@H]2[C@H]3[C@H]3[C@H]%13[C@@H]2[C@H]2[C@@H]8C8=C%12c%12c9c9[C@@]%10(c%10c(c41)c5c1c4c%10c9c5c%12[C@H]9[C@H]%10c5c4[C@H]4[C@@H]1[C@@H]6[C@@H]([C@@H]73)[C@H]1[C@@H]%13[C@H]([C@@H]%10[C@@H]41)[C@@H]2[C@@H]89)C%11(C(=O)OC(=O)N)C(=O)OC(=O)N | C65H6N2O6 |  | 5160,863 |
| 150 |  | c12c3c4[C@H]5[C@H]6c7c3c3c2c2c8c9c1c1[C@H]%10c9c9c%11c8c8c2c2c3c3[C@@]%12%13c2c2c8[C@H]8[C@@H]%11c%11c9c9[C@@H](c%14c(c41)c5c1c4c%14c9c5c%11c9c%11c5c4c4c1c6c(c73)c1[C@@]%12(c(c%11c41)c2c89)C(=C[C@H](N%13C(=O)O)C(=O)N)C(=O)O)N([C@H](/C=C\%10/C(=O)O)C(=O)N)C(=O)O | C72H68N4O10 | S | 5650,295 |
| 151 |  | c12c3c4[C@H]5[C@H]6[C@H]7[C@@H]3[C@H]3[C@@H]2[C@H]2c8c9c1c1[C@]%10%11c9c9c%12c8[C@@H]8[C@H]2[C@@H]2[C@H]3[C@H]3[C@H]%13[C@@H]2[C@H]2[C@@H]8[C@H]8[C@@H]%12c%12c9c9[C@@]%10(c%10c(c41)c5c1c4c%10c9c5c%12[C@H]9[C@H]%10[C@@H]5[C@@H]4[C@H]4[C@@H]1[C@@H]6[C@@H]([C@@H]73)[C@H]1[C@@H]%13[C@H]([C@@H]%10[C@@H]41)[C@@H]2[C@@H]89)C%11(C(=O)OCC)C(=O)OCC | C67H10O4 |  | 5171,237 |
| 152 |  | c12c3c4[C@H]5[C@H]6c7c3c3c2c2c8c9c1c1[C@]%10%11c9c9c%12c8c8c2c2c3c3[C@@]%13%14c2c2c8[C@H]8[C@@H]%12c%12c9c9[C@@]%10(c%10c(c41)c5c1c4c%10c9c5c%12c9c%10c5c4c4c1c6c(c73)c1[C@@]%13(c(c%10c41)c2c89)C(=C(C(=C%14C(=O)O)O)C(=C(O)O)O)C(=O)O)C(=C(C(=C%11C(=O)O)C(=C(O)O)O)O)C(=O)O | C76H70O16 | S | 5909,081 |
| 153 |  | c12c3c4[C@H]5[C@H]6c7c3c3c2c2c8c9c1c1[C@H]%10c9c9c%11c8c8c2c2c3c3[C@@]%12%13c2c2c8[C@H]8[C@@H]%11c%11c9c9[C@@H](c%14c(c41)c5c1c4c%14c9c5c%11c9c%11c5c4c4c1c6c(c73)c1[C@@]%12(c(c%11c41)c2c89)C(=C(C(=C%13C(=O)O)O)O)C(=O)O)/C(=C(\C(=C/%10\C(=O)O)\O)/O)/C(=O)O.[H] | C72H64O12 | S | 5500,136 |
| 154 |  | c1(nc(nc(n1)NC)NC)N/N=C/c1ccc([C@H]2[C@@]34c5c6c7c8c9c%10[C@H]%11[C@H]%12[C@H]%13[C@@H]9[C@H]9[C@@H]8[C@H]8c6c6c%14c5c5c%15[C@@]3(c3c(c%10c47)c%11c4c7c3c%15c3c%10c5[C@H]%14[C@@H]5[C@@H]%11[C@H]6[C@H]8[C@@H]6[C@H]9[C@H]8[C@H]9[C@@H]6[C@@H]%11[C@H]6[C@@H]5[C@@H]%10[C@H]5[C@@H]3[C@@H]7[C@H]3[C@@H]4[C@@H]%12[C@@H]([C@@H]%138)[C@H]4[C@@H]9[C@@H]6[C@@H]5[C@@H]34)CN2C)cc1 | C75H80N8 | S | 6650,541 |
| 155 |  | c1(nc(nc(n1)N(C)C)NC)N/N=C/c1ccc([C@H]2[C@@]34c5c6c7c8c9c%10[C@H]%11[C@H]%12[C@H]%13[C@@H]9[C@H]9[C@@H]8[C@H]8c6c6c%14c5c5c%15[C@@]3(c3c(c%10c47)c%11c4c7c3c%15c3c%10c5[C@H]%14[C@@H]5[C@@H]%11[C@H]6[C@H]8[C@@H]6[C@H]9[C@H]8[C@H]9[C@@H]6[C@@H]%11[C@H]6[C@@H]5[C@@H]%10[C@H]5[C@@H]3[C@@H]7[C@H]3[C@@H]4[C@@H]%12[C@@H]([C@@H]%138)[C@H]4[C@@H]9[C@@H]6[C@@H]5[C@@H]34)CN2C)cc1 | C76H82N8 | S | 6726,396 |
| 156 |  | c1(nc(nc(n1)N(C)C)N(C)C)N/N=C/c1ccc([C@H]2[C@@]34c5c6c7c8c9c%10[C@H]%11[C@H]%12[C@H]%13[C@@H]9[C@H]9[C@@H]8[C@H]8c6c6c%14c5c5c%15[C@@]3(c3c(c%10c47)c%11c4c7c3c%15c3c%10c5[C@H]%14[C@@H]5[C@@H]%11[C@H]6[C@H]8[C@@H]6[C@H]9[C@H]8[C@H]9[C@@H]6[C@@H]%11[C@H]6[C@@H]5[C@@H]%10[C@H]5[C@@H]3[C@@H]7[C@H]3[C@@H]4[C@@H]%12[C@@H]([C@@H]%138)[C@H]4[C@@H]9[C@@H]6[C@@H]5[C@@H]34)CN2C)cc1 | C77H24N8 |  | 6802,335 |
| 157 |  | c1(=[NH]/N=C/[C@@H]2C=C[C@@H]([C@H]3[C@@]45c6c7c8c9c%10c%11[C@H]%12[C@H]%13[C@H]%14[C@@H]%10[C@H]%10c9c9c7c7c%15c6c6c%16[C@@]4(c4c(c%11c58)c%12c5c8c4c%16c4c%11c6[C@H]%15[C@@H]6[C@@H]%12[C@H]7[C@H]9[C@@H]7[C@H]%10[C@H]9[C@H]%10[C@@H]7[C@@H]%12[C@H]7[C@@H]6[C@@H]%11[C@H]6c4c8[C@H]4[C@@H]5[C@@H]%13[C@@H]([C@@H]%149)[C@H]5[C@@H]%10[C@@H]7[C@@H]6[C@@H]45)CN3C)C=C2)nc([nH]c(n1)[NH3])[NH3] | C73H78N8 | S | 6230,439 |
| 158 |  | C12=C3[C@@H]4[C@@H]5C6=C2C2=C7[C@H]8[C@@H]6C6=C9[C@@]%10%11[C@]%12(C(=C56)[C@H]5[C@@H]4C4=C6[C@@H]%13[C@H]5C%12=C5[C@H]%12[C@@H]%13C%13=C%14[C@H]%15[C@@H]%16[C@@H]%17[C@H]%18[C@@H]([C@H]2[C@H]1[C@H]([C@@H]([C@@H]%16[C@H]6%13)[C@H]34)C%17(P(=O)(OCC)OCC)P(=O)(OCC)OCC)C1=C7C2=C3[C@@H]4[C@H]6[C@H](C%12=C%14[C@H]7C%15=C%18[C@@H]1[C@@H]3[C@@H]67)C5=C%10[C@@H]4[C@H]9[C@H]82)C%11(P(=O)(OCC)OCC)P(=O)(OCC)OCC | C78H42O12P4 |  | 6620,568 |
| 159 |  | c12C3=C4[C@H]5[C@@H]6[C@@H]7C8=C9c%10c(c1[C@@H]59)c1c5c2C2=C9[C@H]%11[C@@H]5C5=C%12[C@@]%13%14[C@]%15(c(c%10[C@H]%10[C@@H]8[C@@H]8[C@@H]%16C(=C%15%10)C%10=C%13[C@H]%13[C@H]%15[C@H]%17[C@@H]%10C%16=C%10C(=C78)[C@H]7[C@@H]6C(=C6[C@@H]([C@@H]32)[C@@H]2C3=C(C%15=C([C@@H]%11[C@@H]%12%13)[C@H]92)[C@@H]%17[C@@H]%10C7=C63)C4(C(=O)O)C(=O)O)c15)C%14(C(=O)O)C(=O)O | C66H4O8 |  | 4966,781 |
| 160 |  | C12=C3[C@@H]4[C@@H]5[C@@H]6[C@H]2c2c7=C1[C@]1([C@]8(C9=c7c7[C@H]%10[C@H]%11[C@@H]%12[C@@H]%13[C@H]%14[C@@H]%15C%16=C%17[C@H]%18[C@H]%19[C@@H]%20[C@@H]%21[C@H]%22[C@@H]([C@H]4[C@@H]4C%23=C5C5=C%24[C@]%25([C@@]%23(C(=C([C@H]%16%20)C%15=C%25[C@@H]%13[C@H]%24[C@@H]%13[C@H]([C@@H]65)c2c7[C@H]%12%13)[C@H]%214)O)O)C3=C1[C@H]([C@@H]%19%22)C8=C%18C(=C9%10)[C@H]%17[C@@H]%11%14)O)O | C60H4O4 |  | 4192,208 |
| 161 |  | C(CC[NH3])OC(=O)C1([C@@H]2[C@H]3C4=C5[C@@H]6[C@@H]7C8=C4C4=C9[C@H]%10[C@@H]8C8=C%11[C@@]%12%13[C@]%14(C(=C78)[C@H]7[C@@H]6C6=C8[C@@H]%15[C@H]7C%14=C7[C@H]%14[C@@H]%15C%15=C%16[C@H]%17[C@H]([C@@H]1[C@H]1[C@@H]([C@@H]34)[C@@H]3[C@H]4[C@H]%18C%19=C([C@@H]%10[C@@H]%11[C@H]%10C%12=C7[C@H]7C%14=C%16[C@@H](C%17=C14)[C@@H]%18[C@H]7[C@@H]%19%10)[C@H]93)[C@H]([C@H]2[C@H]56)[C@H]8%15)C%13(C(=O)OCCC[NH3])C(=O)OCCC[NH3])C(=O)OCCC[NH3] | C78H36N4O8 |  | 6765,457 |
| 162 |  | [C@H]12[C@@H]3[C@H]4C5=C6[C@H]7[C@@H]3C3=C8[C@@]9%10[C@]%11(C(=C23)[C@H]2[C@H]3[C@@H]1C1=C4C4=C%12[C@@]%13%14C%15=C%16C(=C3[C@@]1%13C%14(C(=O)OCCC[NH3])C(=O)OCCC[NH3])[C@@H]1[C@H]2C%11=C2[C@H]3[C@@H]1[C@@H]%16[C@H]1[C@H]%11[C@@H]%15[C@@H]%12[C@H]%12[C@@H]([C@H]54)[C@@H]4[C@@H]5C%12=C%11[C@H]%11[C@@H]%12[C@@H]%13[C@H](C3=C1%11)C2=C9[C@H]1[C@@H]%13[C@H]([C@H](C7=C81)[C@H]64)C([C@H]5%12)(C(=O)OCCC[NH3])C(=O)OCCC[NH3])C%10(C(=O)OCCC[NH3])C(=O)OCCC[NH3] | C87H110N6O12 | S | 7416,958 |
| 163 |  | C12=C3[C@@H]4[C@@H]5[C@@H]6[C@@H]([C@@H]7[C@@H]8[C@H]2C2=C9C%10=C8C8=C%11[C@@]%12%13[C@]%14(C(=C78)[C@@H]6C6=C7[C@@H]5[C@H]5[C@H]8[C@@H]4[C@H]4[C@@H]1[C@@H]2[C@@H]1[C@@H]2[C@@H]([C@H]8[C@@H]8C%15=C5[C@H]7[C@@H]5C(=C%146)C6=C%12C7=C%12[C@@]%14%16[C@@]6(C5=C%15C5=C%14[C@@H]6[C@H]%12[C@H]([C@@H]%10[C@@H]%117)C9=C1[C@@H]6[C@H]2[C@H]85)C%16(C(=O)OCCC[NH3])C(=O)OCCC[NH3])C4(C(=O)OCCC[NH3])C(=O)OCCC[NH3])C%13(C(=O)OCCC[NH3])C(=O)OCCC[NH3])C3(C(=O)OCCC[NH3])C(=O)OCCC[NH3] | C96H**128**N8O16 | S | 7885,207 |
| 164 |  | c12c3c4c5c6c7c3c3c2c2c8c9c1c1[C@]%10%11c9c9c%12c8c8[C@]%132[C@@]2(c3c3[C@H]%14C2=C2c8c8c%12c%12c9c9[C@@]%10(c%10c(c41)c5c1c4c%10c9c5c%12c9[C@H]%10c5c4C4=C5[C@H]%10[C@H]([C@@H]2c89)[C@H]%14C(=C(c73)[C@H]6[C@H]14)C5([C](=O)=O)[C](=O)=O)C%11([C](=O)=O)[C](=O)=O)C%13([C](=O)=O)[C](=O)=O | C69H6O12 |  | 5267,719 |
| 165 |  | C12=C3[C@@]45[C@]6(C7=C8[C@]9([C@]%10(C7=C3[C@@]37[C@@]2(C2=C%11[C@]%12([C@@]1(C1=C4[C@]4%13[C@@]%14(C%15=C1[C@]1%12[C@@]%12(C%16=C%11C%11=C%17[C@@]%18%19[C@@]%16(C%16=C%20[C@@]%21%22[C@@]([C@]%15([C@]%12%16O)O)(C%14=C%12[C@@]([C@]64O)([C@@]48[C@@]6([C@]8%12[C@]%21(C%12=C%14C%15=C([C@@]%18([C@@]%20%12O)O)C%17=C%12[C@]([C@@]2%11O)(C3=C%10[C@@]2%12[C@@]%15(C9=C6%14)O2)O)C8(P(=O)(OCC)OCC)P(=O)(OCC)OCC)O4)O)O%22)O%19)O1)O%13)O)O)O7)O)O)O5 | C69H62O26P2 | S | 5974,526 |
| 166 |  | [C@H]12[C@@H]3[C@H]4c5c6c7c3c3c2c2c8c9[C@@H]1[C@@H]1c%10c9c9c%11c8c8c2c2c3c3[C@@]%12%13c2c2c8[C@@]8%14[C@@]%11(c%11c9c9c%10c%10c([C@H]41)c5c1c4c%10c9c5c%11c9c%10[C@@]%115[C@@]4(c4c1c6c(c73)c1[C@@]%12(c(c%10c41)c2c89)C%13(C(=O)O)C(=O)O)C%11(C(=O)O)C(=O)O)C%14(C(=O)O)C(=O)O | C69H6O12 |  | 5174,908 |
| 167 |  | c12c3c4c5c6c7c3c3c2c2c8c9c1c1[C@]%10%11c9c9c%12c8c8[C@]%132[C@@]2(c3c3[C@H]%14C2=C2c8c8c%12c%12c9c9[C@@]%10(C%10=C(c41)[C@@H]5[C@@H]1[C@@H]4[C@H]%10c9c5c%12c9[C@H]%10C5=C4[C@H]4[C@@H]1[C@@H]6C(=C1[C@@H]%14[C@H]([C@@H]%10[C@@H]4C1(C(=O)O)C(=O)O)[C@@H]2c89)c73)C%11(C(=O)O)C(=O)O)C%13(C(=O)O)C(=O)O | C69H**62**O12 | S | 5332,573 |
| 168 |  | c12c3c4c5c6c7c8c9c%10c%11c%12c%13c%14c%15c%16c%17c%18c(c%19c2c2c%20c%21c(c6c32)c2c3c%21c6c%21c%22c(c%18c%19c%20%21)c%18c%17c%15c%15c%17c%18c%18c%19c%17c%17c(c%10c%10c%17c%17c%19c(c6c%22%18)c3c%17c(c8%10)c72)c%15c%11%14)c2c1c(c%13c%162)c4c%12c59 | **C60** |  | 3938,286 |
| 169 |  | [C@@H]12[C@@H]3[C@H]4[C@H]5[C@H]6[C@H]7[C@@H]3[C@H]3[C@H]8[C@@H]1C1=C9[C@@H]%10[C@H]8[C@@H]8C%11=C3[C@H]7[C@@H]3[C@@H]7[C@H]6[C@@H]6[C@@H]%12[C@H]5[C@H]5[C@H]%13[C@H]%14[C@@H]%12[C@H]%12[C@H]%15[C@@H]6[C@@H]7[C@H]6[C@H]7[C@@H]3[C@@H]%11[C@@H]3[C@H]%11[C@@H]%16[C@H]%17[C@H]%18[C@@H]%19[C@@H]%20[C@H]%21[C@@H]%22C%23=C%24[C@@H]([C@H]%13[C@@H]%13[C@H]([C@@H]45)[C@H]2[C@H]1[C@@H]([C@H]%24%13)[C@@H]%22[C@H]9[C@@H]%20[C@H]%10[C@@H]([C@H]%16%19)[C@H]83)[C@H]1[C@@H]%14[C@@H]%12[C@H]2[C@H]3[C@@H]%15[C@@H]6[C@H]([C@@H]%17[C@H]3[C@@H]3[C@H]2[C@H]1[C@H]%23[C@H]%21[C@H]%183)[C@@H]7%11 | **C80H2** |  | 4398,496 |

**Tables S2-S8.** The classification of FDs dependent on type of bond of substituent groups attached to the fullerene C60 core.

**Table S2.** **GROUP 1:** FDs with functional groups attached to the C60 core with single bond with indication the level of binding activity.

| **Attachment to the C60 core with** | Schematic illustration | Bscores range, activity level |
| --- | --- | --- |
|  | **GROUP 1 Alkyl substituent** |  |
| **(1).** Alkyl substituent-single bond containing alkyl groups |  | 6923-5422,  **A-active**  The most active **FD6** has a longest alkyl chain with alkenyl group (double unsaturated -C=C- ) in the middle of the chain (Bscores= 6922.5)  The **FD5** (5421.7) has the same long chain as **FD7** (5585.3), but without amino group -NH_2_ and has smaller activity by 163.6.  The most of compounds in this group belong to amino acids and can play significant role in processes of metabolism. |
|  |  |  |

**Table S3.** **GROUP 2**: FDs with functional groups attached to the C60 core with cyclopropane 3-membered ring with indication the level of binding activity.

| Attachment to the C60 core with | Schematic illustration | Bscores range, activity level | |
| --- | --- | --- | --- |
| **GROUP 2 Cyclopropane ring** | | | |
| **(2).** Cyclopropane 3-membered ring | Group 2a- attached to the C60 core with cyclopropane 3-membered ring and containing two benzene rings | 7257-6164  **A-High active**  The most active **FD4** contains two benzene rings and 8-CH2-, 2carboxyl-COOH and 2amide groups (Bscores=7257.4)  Then follows **FD10** (6779.5) that has substituent from benzene ring only from one side and containing 3-CH2-; 3-hydroxyl groups-OH and 1amide group. This caused the reduction of Bscores value by 477.9 in comparison with **FD4**. The Crown-6 ether bonded to benzene ring (**FD17**) occupied the next place by activity (Bscore=6766.0). **FD55** and **FD2** are different by length of side chain (-CH2-). **FD55** has 6 -CH2-, while **FD2** has 4-CH2- which caused the reduction of Bscores value from 6456.1 to 6163.5 (by 292.6). | |
|  | Group 2b attached to the C60 core with cyclopropane 3-membered ring and containing phenyl group | | 6048-5165  **A-active-**  **FDs 52,51, 53, 1,** containing two phenyl groups  **M- moderate active-**  **FDs 60, 59, 54,** containing only one phenyl group in side chain of substituent.  Increase of activity by presence of more phenyl groups and –CH3.  It is evident that presence of phenyl groups and additionally some others groups like –CH_3_ increases the activity of FD by approximately 900 Bscores.  The fused 6-membered ring here contributed to more high activity than phenyl group.  The structure of **FD 60** differs from the structure of **FD59** by presence of -OH in benzene ring that resulted the increase of binding activity of **FD60** by 83 Bscores. |
|  | Group 2c attached to the C60 core with cyclopropane 3-membered ring and containing ammonium groups | 7885-6766  **A-High active**  Increase of activity with growing the number of ammonium groups  from 4 to 8 NH_3_^+^ by 1120 BScores | |
|  | Group 2d attached to the C60 core with cyclopropane 3-membered ring and containing phosphonate groups | 6621-5975  **A-active**  2 more phosphonate groups and 4 more ethyl groups-CH2-CH3 contributed to increase of activity by 646 | |
|  | Group 2e attached to the C60 core with cyclopropane 3-membered ring and containing carboxyl groups and fluorine | 5333-4413  Between  **M-moderate and L- low active**  Increasing number of –COOH from 1 to 6 resulted in increasing of Bscores by 920.  The saturated **FD167** (C69**H62**O12) in this group was more active by 157.7 Bscore than unsaturated **FD166 (**C69**H6**O12).  The activity of FDs was reduced from 5333 to 4212 with the change of substituents in the following order: 6 carboxyl groups (-COOH) in saturated **FD167**, 6 carboxyl groups (-COOH) in unsaturated **FD166**, 4 carboxyl groups (-COOH) in **FD159**, 2 carboxyl groups in **FD 62** and 1 carboxyl group in **FD 57**.  It should be noted that in comparison with pristine fullerene C60 (**FD168**=3938.3) the addition of carboxyl group –COOH in **FD57** (4412.7) caused the increase of binding activity by 474.4. | |
|  | Group 2f attached to the C60 core with cyclopropane 3-membered ring and containing ethyl, amide, amine, methyl and carboxyl groups | 5171-4954  **M-moderate and L- low active- (on the border of low active)**  The reduction of Bscores by 217 was obtained with the change of substituents in the following order: ethyl (-CH2-CH3), amide (-CONH2), amine (-NH2), methyl (–CH3) and carboxyl (-COOH).  There was small differences in binding activity between **FD151** (5171.2) with ethyl -CH2-CH3 group and **FD149** (5160.9) with amide-CONH2 group equal only 10. The differences between **FD149** (5160.9) with amide-CONH2 group and **FD148** (5113.1) with amine-NH2 group was 47.8 and the differences in binding activity of methyl-CH3 group containing **FD56** (5084.9) and **FD148** (5113.1) with amine-NH2 group was 28.2. The less active in this row was **FD147** (4954.1) containing carboxyl groups -COOH. The difference between **FD56** and **FD147** was 130. | |

**Table S4.** **GROUP 3:** FDs contained the substituent groups attached to the C60 core with pyrrolidine (5-membered ring) or pyridine (6-membered) heterocycles containing nitrogen with indication the level of binding activity.

| Attachment to the C60 core with | Schematic illustration | Bscores range, activity level |
| --- | --- | --- |
| **GROUP 3 Pyrrolidine ring** | | |
| **(3).** Pyrrolidine (5-membered ring) | Group 3a attached to the C60 core with pyrrolidine (5-membered ring) and containing aromatic nitrogen | 6802-6230  **A-High active**  The activity of FDs here decreases dependent on substituent in the following order: 4-CH3; 3-CH3; 2-CH3; 1-CH3+1-NH2; 2-NH2; 2-NH3^+^.  The FDs with the largest number of -CH3 groups are the most active. The binding activity was dropped down by reduction from 4 -CH3 groups in **FD156** (6802.3) to 1-CH3 in **FD113** (6414.5) by 387.8. |
|  | Group 3b attached to the C60 core with pyrrolidine ring | 5490-4782  **M-moderate and L- low active**  The activity of FDs here decreases dependent on substituent in the following order: 6-COOH; 3 -NO_2_ and 3 ketone groups; 1-CH_3_, 2 -NO_2_ and 2 ketone groups; 1 -NH_2_ and 2 amide groups.  The most active in this group of FDs is **FD77** (5490.0) containing 2 symmetrical groups with total 6 carboxyl groups. Then follow **FD129** (5221.9) containing 3 nitro groups -NO2 and 2 ketone groups. The **FD127**(5079.2) containing 3 nitro groups -NO2 and 3 ketone groups and **FD125** (5075.2) containing 1 methyl group -CH3, 2 nitro groups -NO2 and 2 ketone groups have similar binding activity. The close binding activity have **FD28** (4936.0) containing 1 amine group -NH2 and 2 amide groups and **FD19** (4934.5) containing 2 symmetrical groups with total 2 methyl groups. The **FD128** (4807.8) with 1 methyl group -CH3 and 2 ketone group an **FD126** (4782.2) containing 1 methyl group -CH3 and 2 nitro groups NO2 also have the similar binding activity |
| Pyridine (6-membered) heterocycles contaning nitrogen | Group 3c attached to the C60 core with 6-membered cycle and containing in the side chain pyridine ring or attached to the C60 core with pyridine ring or 6-membered heterocycle containing nitrogen | 6709-5650  **A-High active**  Pyridine rings as well as benzene rings, probably, contribute to the high binding activity.  Pyridine containing FDs appeared to be at the highest level of binding activity (6709.4 (**FD36**)- 6356.6 (**FD35**)).  **FD36** contains 4-CH3 methyl groups; 2 ether groups -O-; 2 ketone groups, while **FD35** contains 4 amide groups. The activity of **FD36** was higher by 352. 8 then **FD 35**. We suggest that methyl groups –CH3 in this case can cause the higher binding activity.  The saturated **FD 146** has also the high level of binding activity (6555.7). |

**Table S5.** **GROUP 4:** FDs contained the substituent groups attached to the C60 core with 6-membered (cyclohexan) ring) with indication the level of binding activity.

| Attachment to the C60 core with | Schematic illustration | Bscores range, activity level |
| --- | --- | --- |
| **GROUP 4 Six membered ring** | | |
| **(4).** 6-membered (cyclohexane) ring | Group 4a attached to the C60 core with 6-membered (cyclohexane) ring | 5704-4671  **L- low active and M-moderate active**  The increasing of binding activity goes in the following order of presence of functional groups: -NO_2_, -NH_2_, -OH, -COOH, amide groups. The most active FDs contains ether –O-, -CH_3_ groups. The longest chains possess the highest activity.  The **FD87** (4670.9) containing one nitro group- NO2 and one amine group-NH2 is less active than **FD90** (4703.7) containing 2 nitro groups- NO2 by 32.8 binding scores. Then follows the **FD74** (4750.9) containing 2 amino groups and **FD73** (4777.5) containing 4 amino groups. These FDs have binding score activity approximately less than 5000 (low active).  The most active in this group of FDs appeared to be **FD70** (5431.2) containing the long chain 2 groups: -O-CH2(CH3)_2._  The **FD85** (5249.2) in comparison with **FD86** (4896.1) has two more hydroxyl groups (-OH). As a result, the binding activity in this case was increased by 353.1. The **FD63** (5334.3) in comparison with **FD72** (4901.0) has two more groups –CH2(CH3)_2_. As a result, the binding activity here was increased by 433.3. **FD66** (4972.6) has two carboxyl groups –COOH in comparison with **FD64** (5280.3) containing two groups –CH2(CH3)_2_. As a result, the binding activity here was increased by 307.7. For this groups of FDs it is evident that groups –CH2(CH3)_2_ plays a significant role in increase of binding activity of FDs. The presence of –COOH groups in **FD66** (4972.6) instead of –OH groups in **FD47** (4787.1) caused the increase of binding activity by 185.5. The increasing of binding activity goes in the following order of presence of functional groups: -NO2, -NH2, -OH, -COOH, amide groups. The most active FDs contains ether –O-, -CH3 groups. The longest chains possess the highest activity. |
|  | Group 4b connected to C60 core with six membered (cyclohexane) ring and containing =N group connected to 6-membered cycle | 5488-5292  **M-moderate active**  The presence of -CH_3_ or -O-CH_3_ increases the binding activity approximately by 192 (**FD11** vs.**FD15**). |

**Table S6.** **GROUP 5:** FDs contained the substituent groups attached to the C60 core with benzene (aromatic 6-membered) ring with indication the level of binding activity.

| Attachment to the C60 core with | Schematic illustration | Bscores range, activity level |
| --- | --- | --- |
| **GROUP 5 Benzene ring** | | |
| **(5).** Benzene (aromatic 6-membered) ring | Group 5a containing amide -NH-(C=O)-, amine –NH2-, carboxyl –COOH, hydroxyl –OH, methyl –CH3 groups | 5471-4765  **M-moderate and**  **L- low active**  Addition of 8-OH increases binding activity by 188.5; 8-OH is more active than 4-OH by 409.2; 2 amides are more active than 2 amine by 257.3; 4-COOH is more active than 4-OH by 495; 4-OH was more active than 4-F by 443.9; 2-OH is more active than 1-OH by 154.2 and 8NH2 is more active than 8 –OH by 136.2  **FD13** (5704.2) contains the longest aside chain with ester group, -CH2-, -CH2-CH2-, 1-COOH and experienced the highest binding activity in this group of FDs. Then follows **FD48** (saturated) (5466.8) containing two groups with 4-COOH, 4-OH and **FD81 (**5470.9) containing four amide groups and 2-CH2-. |
|  | Group 5b1- the most active FDs connected to C60 core with benzene ring and containing 8-14 nitro groups –NO2. | 7120-5886  **A-High active**  FDs containing 8-14  –NO_2_  The reduction of nitro groups from 14 to 12 resulted in reduction of Bscores by 291.8, while the reduction of nitro groups from 14 to 8 resulted in reduction of Bscores by 1111.9.  **FD123** as well as **FD122** are symmetrical molecules. Both of them contain eight nitro groups. The **FD123** in comparison with **FD122** has additional four ketone groups. This results in increasing the Bscores by 285.9 (from 5885.5 in **FD122** to 6171.4 in **FD123** |
|  | Group 5b2 moderate and low active FDs containing –NO2 | 5580-4765  **M-moderate active and**  **L- low active**  In the most cases the most active are FDs with the greater number of –NO_2_ equal to 6 or 4.  1-NO_2_ is more active than 1-NH_2_ by 144;  4-NO_2_ is more active than-2 -NO_2_ by 175.2;  2-COOH is more active than 2-NO_2_ by 146.2 and saturated was greater than unsaturated by 148.1  **FD 116, 117** (containing 6 –NO2), **FD 115** (containing 4-NO2) have a Bscores greater than 5500. They are on the border between moderate and active FDs. Then follow FDs containing 4-NO2 (**FD 91, 104, 103, 102**) with Bscores, approximately, between 5300 and 5400. If we compare **FD88** vs. **FD89** we can find that 2-NO2 in comparison with presence of 1-NO2 and 1 -NH2 caused the increase of Bscores by 114. Comparing **FD 99** vs. **FD 89**, one can see that the presence of four -NO2 in comparison with two -NO2 caused the increasing activity by 175.2. In **FD99** vs. **FD100** the presence of 2 -NO2 instead of 2 -NH2 caused the increasing Bscores by 92.7. The addition of 2-COOH in **FD 105** instead of two -NO2 in **FD99** caused the increasing activity by 146.2 Bscores. Saturated **FD 101** is more active in comparison with unsaturated **FD 99** by 148.1 |
|  | Group 5c containing nitrile ≡N and fluorine –F groups | 6100-5036  **A-High active and M-moderate active**  Nitrile was more active than fluorine-F. Amide is more active than amine. –NO_2_ is more active than -F |
|  | Group 5d containing phenyl group bonded to benzene ring attached to C60 core | 5809-5413  **The boarder of moderate active and active.**  The most active FDs containing -NO_2_, then follows –CH_3_ and –NH_2_.  The small decrease of activity from 3-NH_2_ to 3-F and 3-OH.  The activity of –NO_2_ in para position is higher than in orto- position in benzene ring.  If we compare the simplest structures in this group (**FD39**, **FD133** and **FD 38**) we can notice that activity drops down from **FD39** containing 3-NH2 to **FD133** containing 3-F by 39.3 and from **FD133** to **FD38** containing 3-OH groups by 59.9. Comparing **FD138** and **FD145**, having the same functional groups connected to benzene ring one can notice that activity of **FD145** with –NO2 in para-position is more active then **FD138** with –NO2 in orto- position by 75.5 Bscores. |
|  | **Group 5e** | 6709- 5673  **A-High active**  The most active are pyridine containing FDs. Nitro groups -NO_2_ are more active than –OH.  The presence of cycle groups supported the high activity.  The most active are pyridine containing FDs : **FD36** (Bscores=6709.4) and **FD35** (Bscores=6356.6). If we compare **FD140** and **FD46** containing bridged bicycle ring we can notice than FD with nitro groups –NO2 (**FD140**) is more active then FD with –OH group (**FD46**) by 157 BScores. |

**Table S7.** **GROUP 6**: FDs contained the FDs substituent s attached to the C60 core with fused pair of 6-membered rings with indication the level of binding activity.

| Attachment to the C60 core with | Schematic illustration | Bscores range, activity level |
| --- | --- | --- |
| **GROUP 6 Fuzed pair of 6-membered rings** | | |
| **(6).** Fused pair of 6-membered rings  **(GROUP 6)**, | Group 6 connected to C60 core with double 6-membered rings | 5754-5326  **Moderate to active FDs**  The activity of FD45 containing fuzzed 6 membered rings is higher than activity of FD40 with benzene ring by 735.2.  The most active were FDs- with –NO_2_, then follows -CH_3_ and –OH  **FD40** with two benzene rings added to the **group 6** in order to compare the activity of FD containing fuzed 6 membered rings with FD containing two benzene rings. Thus, the activity of **FD45** containing fuzed 6 membered rings is higher than activity of **FD40** by 735.2. This group 6 of FDs demonstrated the most active **FD114** with 4 nitro –NO2 groups (5754), then follows FDs containing –CH3 and –OH groups. |

**Table S8.** **GROUP 7:** FDs contained the FDs substituent groups attached to the C60 core with bridged bicycle rings with indication the level of binding activity.

| Attachment to the C60 core with | Schematic illustration | Bscores range, activity level |
| --- | --- | --- |
| **GROUP 7 Bridged bicycle rings** | | |
| **(7).** Bridged bicycle rings (group **7**) | Group 7 connected to C60 core with bridged bicycle groups | 5439-5065  **M-Moderate active**  The activity here, probably, mostly, dependent on presence of double bonds in bridge. |

**Figure 1S.** The differences in binding activity (∆Bscores) between the most active FDs and pristine fullerene C_60_.

# The list of considered proteins:

1. ***Enzymes:***

**OXIDOREDUCTASE** is an enzyme that catalyzes the transfer of electrons from one molecule, the reductant, also called the electron donor, to another, the oxidant, also called the electron acceptor.

**HYDROLASE** is a class of enzyme that commonly perform as biochemical catalysts that use water to break a chemical bond, which typically results in dividing a larger molecule to smaller molecules.

**ISOMERASES** are a general class of enzymes that convert a molecule from one isomer to another. Isomerases facilitate intramolecular rearrangements in which bonds are broken and formed.

**TRANSFERASE** is any one of a class of enzymes that enact the transfer of specific functional groups (e.g. a methyl or glycosylic group) from one molecule (called the donor) to another (called the acceptor).

**AMINOTRANSFERASES** are enzymes that catalyze a transamination reaction between an amino acid and an α-keto acid. They are important in the synthesis of amino acids, which form proteins.

**LYASE** is an enzyme that catalyzes the breaking (an "elimination" reaction) of various chemical bonds by means other than hydrolysis (a "substitution" reaction) and oxidation, often forming a new double bond or a new ring structure.

**LIGASE** is an enzyme that can catalyze the joining of two large molecules by forming a new chemical bond, usually with accompanying hydrolysis of a small pendant chemical group on one of the larger molecules or the enzyme catalyzing the linking together of two compounds.

1. **DNA replication**

**GENE REGULATION** refers to the mechanisms that act to induce or repress the expression of a gene. These include structural and chemical changes to the genetic material, binding of proteins to specific DNA elements to regulate transcription, or mechanisms that modulate translation of mRNA.

**TRANSCRIPTION** is the first step in gene expression. It involves copying a gene's DNA sequence to make an RNA molecule. Transcription is performed by enzymes called RNA polymerases, which link nucleotides to form an RNA strand (using a DNA strand as a template).

1. **Receptor- respondent to stimuli**

**MEMBRANE RECEPTOR PROTEIN** relay signals between the cell's internal and external environments.

**HORMONE RECEPTOR PROTEIN** is a receptor molecule that binds to a specific hormone. Hormone receptors are a wide family of proteins made up of receptors for thyroid and steroid hormones, retinoid and Vitamin D, and a variety of other receptors for various ligands, such as fatty acids and prostaglandins

**IMMUNE SYSTEM PROTEINS** are predominantly signaling proteins (often called cytokines), antibodies, and complement proteins.

**SIGNALLING PROTEINS** enable signals in the form of second messengers, hormones, proteins and other biological molecules which are recognized by cell surface receptors, which mediate their responses to other intracellular signaling peptides and proteins, such as transcription factors.

GROWTH FACTORS, which generally considered as a subset of cytokines, refer to the diffusible signaling proteins that stimulate cell growth, differentiation, survival, inflammation, and tissue repair.

**ANTIMICROBIAL ANTITUMOR PROTEINS** or peptides are part of the innate immune response found among all classes of life.

**CELL ADHESION** molecules (CAMs) are a subset of cell adhesion proteins located on the cell surface involved in binding with other cells or with the extracellular matrix (ECM) in the process called cell adhesion. In essence, cell adhesion molecules help cells stick to each other and to their surroundings.

**BIOTIN-BINDING PROTEIN** includes streptavidin, avidin, and NeutrAvidin® protein. Each protein binds four biotins per molecule with high affinity and selectivity. Streptavidin is most commonly used—it is non-glycosylated and exhibits low levels of nonspecific binding.

1. **Transporting molecules**

**TRANSPORT PROTEIN** is a protein that serves the function of moving other materials within an organism.

**LIPID TRASFER PROTEIN** primary function appeared to be in facilitation of long chain fatty acid transport from plasma membrane to intracellular site for β-oxidation.

# The detailed description of neural networks used in the study.

The architecture of CP ANN employed in current study is presented in Figure 2S.

**Figure 2S.** The architecture of CP ANN.

The CP ANN method belongs to self-organizing map technique that often used to analyze the data in multi-dimensional space. The basis of this technique is a non-linear projection from multi-dimensional space onto a two-dimensional map. The topology preserving projection achieved *via* non-linear algorithm known as training. The trained network enables the arrangement of similar objects close to each other. Therefore, it is expected that chemicals with similar structure or properties will form the clusters, which is the case of examination [1S].

The architecture of CP ANN shown in Figure 1S constructed of neurons composed of two layers: input layer (Kohonen layer) containing encoded information of structure expressed as descriptors values and output layer (response). Both layers of neurons are placed exactly one above the other and the output layer has exactly the same layout of neurons as the input one [1S-4S].

Kohonen maps allow us to perform a visualization of the distribution of chemicals in the top map, the distribution of descriptors values in weight levels maps as well as distribution of Bscores values in output layer. The training algorithm of Kohonen’s Self-Organizing Maps (SOM) guarantees that similar molecules activate topologically close neurons in the competitive layer. Projection of molecules to the location of the corresponding winning neurons produces a map, in which neighborhood relations between molecules are largely preserved. As structurally similar molecules tend to have similar activities, then molecules belonging to the same activity class mapped either to the same neuron or to several neighboring neurons [5S]. Moreover, the Kohonen self-organizing map is a very promising method for data partition due to creating a mapping of multidimensional information onto a layer of neurons that preserves the essential content of the information (relationships).

CP ANN, in turn, is a generalization of self-organizing map. Additionally, it takes into account the property (output) values [4S]. Thus, the learning in the input layer in the CP ANN is the same as in Kohonen neural network, i.e., the similarity among input variables determines the arrangement of objects in the input layer map (unsupervised step). When the arrangement is set, the positions of objects projected to the output where the weights are modified in a way that the weights on projected positions are getting similar to the values of corresponding objects (supervised step).

In Figure 2S the inputs x_1_- x_m_ are vector components, which corresponds to m descriptors calculated for all FDs used in the dataset intended for training. In our study we have applied 27 and 10 descriptors (m=27, 10). The distribution of chemicals and their clusters in 2D space examined in the Kohonen top map. Weight levels from one to m are the maps with distribution of particular descriptors from one to m. The output variables expressed in the output layer as values of *BScores.*

The following 2D maps used in the study to analyze the similarities in set of FDs: Kohonen *top map* (with distribution of FDs) and *output layer* *maps* with indication of value of BScores. Additionally, the 27 and 10 weight maps with distribution of values for 27 and 10 descriptors were explored to see the similarities between applied descriptors as well as with output layer represented the distribution of Average BScored.

To perform a CP ANN modelling a special code TRACEANN for Matlab applied. The code was developed by Marjan Tušar (Lab of Chemometrics, National Institute of Chemistry, Slovenia). Further applications and code details reported by Groselj et al. [6S]. The ANN Toolbox performs the classification of multivariate data using the Kohonen mapping method and predictive modelling using CP ANN, which includes visualization (contour plots, 3D visualization, and colored neurons) of Kohonen levels.

Referenses:

1S. Zupan, J.; Novič, M.; Ruisainchez, I. Kohonen and Counterpropagation Artificial Neural Networks in Analytical Chemistry. *Chemometr. Intell. Lab. Syst.* **1997**, 38, 1-23.

2S. Zupan, J.; Gasteiger, J. *Neural Networks in Chemistry and Drug Design*. 2nd Edition. Wiley-VCH Verlag GmbH: Weinheim, 1999.

3S. Mazzatorta, P.; Vračko, M.; Jezierska ,A.; Benfenati, E. Modeling Toxicity by Using Supervised Kohonen Neural Networks, *J. Chem. Inf. Comput. Sci.* **2003**, 43, 485-492.

4S. Vračko, M.; Novič, M.; Zupan, J. Study of Structure-Toxicity Relationship by a Counter-propagation Neural Network. *Anal. Chim. acta* **1999**, 384(3), 319-332.

5S.Kohonen, T. *Self-organizing Maps*. Springer: Berlin, 2001.

6S.Grošelj, N.; Van der Veer, G.; Tušar, M.; Vračko, M.; Novič, M. 2010. Verification of the Geological Origin of Bottled Mineral Water Using Artificial Neural Networks. *Food Chem.* **2010**, 118, 941–947.

# Selection of the most significant proteins

The matrix contained Bscores for 1117 proteins related to 169 fullerene derivatives (FDs) was composed.

The neural network with dimension 5x5 was trained for 100 learning epochs. We have got a Kohonen map 5x5 with distribution of 1117 proteins. The proteins are distributed according with similarity caused by binding activity.

The similar objects fallen close to each other’s in Kohonen maps. It means that proteins located at the same or close neurons or possess a similarity. They were characterized by Euclidean distance.

*Firstly*, we considered proteins located at the same neuron for analyzing the similarity between them.

*Secondly*, we reduced the number of considered proteins located at the same neurons. The descriptors (proteins) with similar Euclidean distance can be omitted. This operation was done to use matrix with reduced numbers of proteins to determine the tendency in activity of proteins related to FDs. Reduction of data lead to the selection the most significant objects. We have got the global generalized view for future study.

*Thirdly*, we can consider the clusters of proteins located at different neurons. This help us to find our classes of proteins which differs for their behavior to considered FDs.

For more details see plots of descriptors (proteins ID) vs. Euclidean distance located on neurons: 1x1; 1x2......, 1x5; 2x1; 2x2....., 2x5;........, 5x1; 5x2;....; 5x5.

**Table A1**. The informative matrix n=5x5 of Kohonen map.

| 5 | 5x1 | 5x2 | 5x3 | 5x4 | 5x5 |
| --- | --- | --- | --- | --- | --- |
| 4 | 4x1 | .... | .... | ..... | 4x5 |
| 3 | .... | ...... | ..... | ..... | ...... |
| 2 | ..... | ..... | ....... | ...... | ...... |
| 1 | 1x1 | 1x2 | 1x3 | 1x4 | 1x5 |
| ny/nx | 1 | 2 | 3 | 4 | 5 |

**Figure 1**. Proteins (ID) located in neuron **n=1x1** dependent on Euclidean distance

The following descriptors were selected from neuron 1x1: 554 and 874.

554- 1M9M- OXIDOREDUCTASE

874- 1XFH- TRANSPORT PROTEIN

**Figure 2**. Proteins (ID) located in neuron **n=2x1** dependent on Euclidean distance

The following descriptors were selected from neuron 2x1: 707, 446, and 285:

707-1R0W-TRANSPORT PROTEIN

446-1ILH-GENE REGULATION

285-1FO4-OXIDOREDUCTASE

**Table B1.** Proteins (ID) located in neuron n=**3x1** with indication of Euclidean distance.

| N | Protein ID | nx | ny | Euclidean distance |
| --- | --- | --- | --- | --- |
| 1 | 951 | 3 | 1 | 669,7625 |
| 2 | 955 | 3 | 1 | 627,6441 |

The following descriptor was selected from neuron 3x1: 951

951-2BCE-HYDROLASE

**Table B2.** Proteins (ID) located in neuron n=**4x1** with indication of Euclidean distance.

| N | Protein number | nx | ny | Euclidean distance |
| --- | --- | --- | --- | --- |
| 1 | 506 | 4 | 1 | 577,2231 |
| 2 | 542 | 4 | 1 | 535,0911 |

The following descriptor was selected from neuron 4x1: 506

506-1JVM-MEMBRANE PROTEIN

**Figure 3**. Proteins (ID) located in neuron **n=5x1** dependent on Euclidean distance

The following descriptors were selected from neuron 5x1: 739, 991

739-1SM2-TRANSFERASE

991-2EU9-TRANSFERASE

**Figure 4**. Proteins (ID) located in neuron **n=1x2** dependent on Euclidean distance

The following descriptors were selected from neuron 1x2: 475, 967, and 730

475-1JBQ-LYASE;

967-2C6Q-OXIDOREDUCTASE;

730-1RTD-TRANSFERASE/DNA

**Figure 5**. Proteins (ID) located in neuron **n=2x2** dependent on Euclidean distance

The following descriptors were selected from neuron 2x2: 468, 347, 628, 151

468-1J59-GENE REGULATION/DNA;

628-1P93-HORMONE RECEPTOR;

151- 1CVI- HYDROLASE

**Figure 6**. Proteins (ID) located in neuron **n=3x2** dependent on Euclidean distance

The following descriptors were selected from neuron 3x2: 975, 1026, 954

975-2CKG-HYDROLASE

1026-2PAW-TRANSFERASE

954-2BH9-OXIDOREDUCTASE

**Figure 7**. Proteins (ID) located in neuron **n=4x2** dependent on Euclidean distance

The following descriptors were selected from neuron 4x2: 757, 191

757-1TC1-TRANSFERASE

191-1DHF-OXIDOREDUCTASE

**Figure 8**. Proteins (ID) located in neuron **n=5x2** dependent on Euclidean distance

The following descriptors were selected from neuron 5x2: 732, 907

732-1S50-MEMBRANE PROTEIN

907-1YVJ-TRANSFERASE

**Figure 9**. Proteins (ID) located in neuron **n=1x3** dependent on Euclidean distance

The following descriptors were selected from neuron 1x3: 246, 844, 603, 895

246- 1EEY- IMMUNE SYSTEM

844- 1W6K- ISOMERASE

603- 1OOQ- OXIDOREDUCTASE

895- 1YAA- AMINOTRANSFERASE

**Table B3.** Proteins (ID) located in neuron n=**2x3** with indication of Euclidean distance.

| 1 | 736 | 2 | 3 | 44,1276 |
| --- | --- | --- | --- | --- |

The following descriptors were selected from neuron 2x3: 736

736-1SFF-TRANSFERASE

**Figure 10**. Proteins (ID) located in neuron **n=3x3** dependent on Euclidean distance

The following descriptors were selected from neuron 3x3: 901, 211

901-1YPP-HYDROLASE

211-1DS6-SIGNALING PROTEIN

**Figure 11**. Proteins (ID) located in neuron **n=4x3** dependent on Euclidean distance

The following descriptors were selected from neuron 4x3: 13, 350

13-1AAQ-HYDROLASE/HYDROLASE INHIBITOR

350- 1HEF- HYDROLASE/HYDROLASE INHIBITOR

**Figure 12**. Proteins (ID) located in neuron **n=5x3** dependent on Euclidean distance

The following descriptors were selected from neuron 5x3: 178, 615, 200, and 953

178-1DB1-GENE REGULATION

615-1OYA-OXIDOREDUCTASE (FLAVOPROTEIN)

724-1RML-GROWTH FACTOR

953-2BFW-TRANSFERASE

**Table B4.** Proteins (ID) located in neuron n=**1x4** with indication of Euclidean distance.

| 1 | 952 | 1 | 4 | 679,8925 |
| --- | --- | --- | --- | --- |
| 2 | 519 | 1 | 4 | 737,2275 |
| 3 | 634 | 1 | 4 | 839,8246 |

The following descriptors were selected from neuron 1x4: 952, 634

952-2BE1-TRANSCRIPTION

634-1PGP-OXIDOREDUCTASE (CHOH(D)-NADP+(A))

**Table B5.** Proteins (ID) located in neuron n=**2x4** with indication of Euclidean distance.

| 1 | 395 | 2 | 4 | 168,9308 |
| --- | --- | --- | --- | --- |
| 2 | 393 | 2 | 4 | 193,5384 |
| 3 | 198 | 2 | 4 | 206,4799 |
| 4 | 394 | 2 | 4 | 209,3118 |
| 5 | 392 | 2 | 4 | 214,6364 |
| 6 | 257 | 2 | 4 | 271,1238 |

The following descriptors were selected from neuron 2x4: 395, 257

395- 1HVL- HYDROLASE (ACID PROTEASE)

257- 1ENT- HYDROLASE/HYDROLASE INHIBITOR

**Figure 13**. Proteins (ID) located in neuron **n=3x4** dependent on Euclidean distance

The following descriptors were selected from neuron 3x4: 430, 573

430- 1IDA- HYDROLASE/HYDROLASE INHIBITOR

573- 1NCO- ANTIBACTERIAL AND ANTITUMOR PROTEIN

**Figure 14**. Proteins (ID) located in neuron **n=4x4** dependent on Euclidean distance

The following descriptors were selected from neuron 4x4: 139, 1021

139- 1CPS- HYDROLASE (C-TERMINAL PEPTIDASE)

1021- 2NCM - CELL ADHESION

**Figure 15**. Proteins (ID) located in neuron **n=5x4** dependent on Euclidean distance

The following descriptors were selected from neuron 5x4: 179, 338

179-1DB4-HYDROLASE/HYDROLASE INHIBITOR

338-1H92- TRANSFERASE

**Figure 16**. Proteins (ID) located in neuron **n=1x5** dependent on Euclidean distance

The following descriptors were selected from neuron 1x5: 937, 613

937-2AEB-HYDROLASE/HYDROLASE INHIBITOR

613-1OW3-GENE REGULATION/SIGNALING PROTEIN

**Figure 17**. Proteins (ID) located in neuron **n=2x5** dependent on Euclidean distance

The following descriptors were selected from neuron 2x5: 512, 958, 930, 361

512-1K3U-LYASE

361-1HKN-GROWTH FACTOR

**Figure 18**. Proteins (ID) located in neuron **n=3x5** dependent on Euclidean distance

The following descriptors were selected from neuron 3x5: 745, 566

745-1SRE-BIOTIN-BINDING PROTEIN

566-1MQ0-HYDROLASE

**Figure 19.** Proteins (ID) located in neuron **n=4x5** dependent on Euclidean distance

The following descriptors were selected from neuron 4x5: 159, 293,890

159-1CZ2-LIPID TRASFER PROTEIN

293-1FYN-TRANSFERASE

890-1XY1-HORMONE-(CRYSTAL STRUCTURE ANALYSIS OF DEAMINO-OXYTOCIN. CONFORMATION 2 FLEXIBILITY AND RECEPTOR BINDING)

**Figure 20**. Proteins (ID) located in neuron **n=5x5** dependent on Euclidean distance

The following descriptors were selected from neuron 5x5: 249, 1017, 591

249-1EFR-HYDROLASE/ANTIBIOTIC

1017-2LGS-LIGASE (AMIDE SYNTHETASE)

591-1OF7-OXIDOREDUCTASE

**Table S9.** Correlation between 27 descriptors (25 drug like descriptors plus (Qpolrz+TD)) and Bscores with correlation coefficients greater than 0.6.

| **Descriptors** | (1) | (3) | (4) | (5) | (7) | (8) | (10) | (11) | (12) | (13) | (15) | (16) | (20) | (23) | (24) | (25) | (26) | (27) |
| --- | --- | --- | --- | --- | --- | --- | --- | --- | --- | --- | --- | --- | --- | --- | --- | --- | --- | --- |
| 1. H-Acceptors |  |  |  |  |  |  |  |  |  |  |  |  |  |  |  |  |  |  |
| 1. H-Donors |  |  |  |  |  |  |  |  |  |  |  |  |  |  |  |  |  |  |
| 1. Total Surface Area | 0,693 |  |  |  |  |  |  |  |  |  |  |  |  |  |  |  |  |  |
| 1. Relative PSA | 0,895 |  |  |  |  |  |  |  |  |  |  |  |  |  |  |  |  |  |
| 1. Polar Surface Area | 0,965 | 0,602 | 0,950 |  |  |  |  |  |  |  |  |  |  |  |  |  |  |  |
| 1. Druglikeness |  |  |  |  |  |  |  |  |  |  |  |  |  |  |  |  |  |  |
| 1. Molweight | 0,820 | 0,954 | 0,611 | 0,746 |  |  |  |  |  |  |  |  |  |  |  |  |  |  |
| 1. cLogP | -0,734 |  | -0,861 | -0,797 |  |  |  |  |  |  |  |  |  |  |  |  |  |  |
| 1. cLogS |  |  |  |  |  |  |  |  |  |  |  |  |  |  |  |  |  |  |
| 1. Electronegative Atoms | 0,982 | 0,704 | 0,873 | 0,946 | 0,843 | -0,703 |  |  |  |  |  |  |  |  |  |  |  |  |
| 1. Stereo Centers |  |  |  |  |  |  |  |  |  |  |  |  |  |  |  |  |  |  |
| 1. Rotatable Bonds |  |  |  |  | 0,739 |  |  |  |  |  |  |  |  |  |  |  |  |  |
| 1. Rings Closures |  |  |  |  |  |  |  |  |  |  |  |  |  |  |  |  |  |  |
| 1. Small Rings |  |  |  |  |  |  |  |  |  | 0,802 |  |  |  |  |  |  |  |  |
| 1. Aromatic Rings |  |  |  |  |  |  |  | -0,847 |  |  |  |  |  |  |  |  |  |  |
| 1. Aromatic Atoms |  |  |  |  |  |  |  | -0,848 |  |  | 0,996 |  |  |  |  |  |  |  |
| 1. sp3-Atoms |  |  |  |  |  |  |  | 0,979 |  |  | -0,850 | -0,852 |  |  |  |  |  |  |
| 1. Symmetric atoms |  |  |  |  |  |  |  |  |  |  |  |  |  |  |  |  |  |  |
| 1. Amides |  |  |  |  |  |  |  |  |  |  |  |  |  |  |  |  |  |  |
| 1. Amines |  |  |  |  |  |  |  |  |  |  |  |  |  |  |  |  |  |  |
| 1. Aromatic Nitrogens |  |  |  |  |  |  |  |  |  |  |  |  |  |  |  |  |  |  |
| 1. Basic Nitrogens |  |  |  |  |  |  |  |  |  |  |  |  | 0,738 |  |  |  |  |  |
| 1. Acidic Oxygens | 0,777 |  | 0,649 | 0,750 |  |  | 0,765 |  |  |  |  |  |  |  |  |  |  |  |
| 1. Non-H Atoms | 0,813 | 0,943 |  | 0,730 | 0,977 |  | 0,830 |  | 0,768 |  |  |  |  |  |  |  |  |  |
| 1. Non-C/H Atoms | 0,982 | 0,704 | 0,873 | 0,946 | 0,843 | -0,703 | 1,000 |  |  |  |  |  |  | 0,765 | 0,830 |  |  |  |
| 1. Polarizability Qp |  | 0,893 |  |  | 0,793 |  |  |  | 0,755 |  |  |  |  |  | 0,842 |  |  |  |
| 1. Top. diameter TD |  | 0,756 |  |  | 0,637 |  |  |  | 0,594 |  |  |  |  |  | 0,679 |  | 0,837 |  |
| BScores |  | 0,875 |  |  | 0,770 |  |  |  | 0,748 |  |  |  |  |  | 0,810 |  | 0,947 | 0,899 |

**Table S10.** The differences between saturated and non-saturated fullerene derivatives.

| **FD_ID** | **Molecule** | **Molecular Formula** | **Saturated (S)** | **QPolrz** | **TD** | **Av_Bscores** | **Functional groups 2D** |
| --- | --- | --- | --- | --- | --- | --- | --- |
| 166 |  | C69H**6**O12 |  | 65,89 | 11 | 5174,91 | 3 groups |
| 167 |  | C69H**62**O12  Saturated | S | 65,72 | 12 | 5332,57 | 3 groups  Saturated fullerene |
| 89 |  | C64H**60**N2O4  Saturated | S | 59,96 | 12 | 4879,47 | Saturated fullerene |
| 90 |  | C64H**6**N2O4 |  | 59,72 | 12 | 4703,68 |  |
| 87 |  | C64H**8**N2O2 |  | 58,42 | 12 | 4670,89 |  |
| 88 |  | C64H**62**N2O2  Saturated | S | 58,38 | 12 | 4764,88 | Saturated fullerene |

The saturated FD 167 is more active in comparison with unsaturated FD 166 by 157.66 Bscores.

The saturated FD 89 is more active in comparison with unsaturated FD 90 by 175.79 BScores.

The saturated FD 88 is more active in comparison with unsaturated FD 87 by 93.99 BScores.
